# Visible Light-Mediated Organoboron-Catalyzed Metal-Free Synthesis of Silanols from Silanes

**DOI:** 10.3390/molecules28104082

**Published:** 2023-05-13

**Authors:** Jinbo Yang, Xiangxue Cao, Lanfeng Wei, Jianshu Zhang, Jinli Zhang, Ping Liu, Liang Xu, Pengfei Li

**Affiliations:** 1 State Key Laboratory Incubation Base for Green Processing of Chemical Engineering, School of Chemistry and Chemical Engineering, Shihezi University, Beisi Road, Xinjiang Uygur Autonomous Region, Shihezi 832003, China; yangjinbo@stu.shzu.edu.cn (J.Y.); caoxiangxue@stu.shzu.edu.cn (X.C.); weilanfeng@stu.shzu.edu.cn (L.W.); zhangjs@shzu.edu.cn (J.Z.); liuping1979112@aliyun.com (P.L.); 2 School of Chemical Engineering and Technology, Tianjin University, Tianjin 300072, China; zhangjinli@tju.edu.cn; 3 State Key Laboratory for Mechanical Behavior of Materials, Frontier Institute of Science and Technology, Xi’an Jiaotong University, Xi’an 710054, China

**Keywords:** photocatalytic oxidation, silane, silanol, aminoquinoline diarylboron

## Abstract

Herein, a four-coordinated organoboron compound, aminoquinoline diarylboron (AQDAB), is utilized as the photocatalyst in the oxidation of silane to silanol. This strategy effectively oxidizes Si–H bonds, affording Si–O bonds. Generally, the corresponding silanols can be obtained in moderate to good yields at room temperature under oxygen atmospheres, representing a green protocol to complement the existing preparation methods for silanols.

## 1. Introduction

**Scheme 1:**
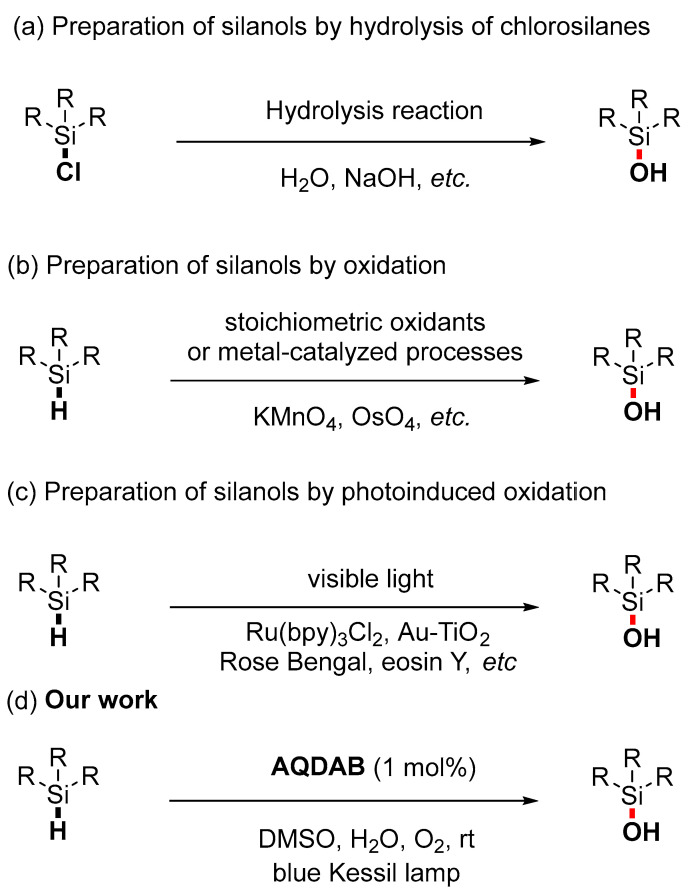
Different strategies for the synthesis of silanols. (**a**) Preparation of silanols by hydrolysis of chlorosilanes. (**b**) Preparation of silanols by oxidation. (**c**) Preparation of silanols by photoinduced oxidation. (**d**) Our developed one-pot synthesis of Silanols from Silanes.

Silanols are widely used in the silicone industry [1,2]. Furthermore, in organic synthesis [2,3], silanols also play an important role as nucleophiles in cross-coupling reactions [3,4], as directing groups to guide C–H bond activation [5,6], or as catalysts to activate the carbonyl moiety [7,8]. In the field of pharmaceutical chemistry, compounds containing the Si–OH moiety are widely used in enzyme inhibitors [9] and isosteres of pheromones [10,11]. Because of these important applications of silanol compounds, their synthesis has become the focus of continuous attention for the organic community. In the past half-century, silanol has been usually prepared by hydrolysis of chlorosilanes (Scheme 1a) [12,13], nucleophilic substitution [14] of siloxanes, or oxidation of silanes (Scheme 1b) [15,16,17,18]. However, these synthetical strategies generally require strict buffer conditions to avoid the production of siloxane, transition metal catalysts, and/or strong oxidants such as permanganates, silver salts, and osmium tetroxides. This damages the atomic economy of such strategies and limits their substrate scope and practical application [15,16,17,18,19,20,21]. Specifically, the use of transition metals can lead to products containing metal residues, which are difficult to clear away and seriously influence the bioactive application of the obtained silanols [22,23,24,25,26,27,28,29,30,31,32,33]. In this regard, the development of new strategies for silanols is highly desired, especially metal-free and more atom-economic and sustainable strategies.

With the development of photoredox catalysis in the last decade, the photo-induced oxidation of silane has also progressed significantly (Scheme 1c) [34,35,36,37]. In 2021, Chen, Fan, and their colleagues reported that under the irradiation of white light, Ru(bpy)_3_Cl_2_ could catalyze the formal dehydrogenative reactions between silanes and water to produce silanols [38]. In 2022, Zhang, Li, and their colleagues reported that silanes could be oxidized to silanols using Au-TiO_2_ as a photocatalyst [35]. In 2018, Wang reported that the conversion of silanes to silanols could be accomplished using Rose Bengal as a photocatalyst, oxygen as an oxidant, and water as an additive [37]. Subsequently, He and Zhang reported the conversion of silane to silanol using eosin Y as a photocatalyst [34]. The study of photoinduced synthesis of silanol inspired us to consider whether the photocatalyst aminoquinolate B,B-diphenyl complex AQDAB [39,40], which was developed by our group and applied in photooxidation reactions [40,41,42], could induce such transformations. Herein, the success of this hypothesis is reported. A range of diverse silanols can be obtained via the catalysis of this boron-based photocatalyst in the absence of metals and additives like strong bases, acids, and oxidants (Scheme 1d).

## 2. Results and Discussion

We initiated the study by investigating the hydroxylation reaction of triphenylsilane **1a** (Table 1). Through screening different reaction conditions, the optimal reaction conditions are obtained as follows: aminoquinolate B,B-diphenyl complex AQDAB as the photocatalyst (1.0 mol%), O_2_ atmosphere, irradiation by a 456 nm blue Kessil lamp, in DMSO/H_2_O (1 mL/50 µL) at room temperature for 36 h (Entry 1). Under optimal conditions, triphenylsilanol **2a** can be isolated with a yield of 88%. Then, the effect of each factor under these conditions was explored through control experiments. In the absence of AQDAB, O_2_, and light sources (Entries 2–4), the reaction will not take place. This indicates that these factors play an important role in the photocatalysis process. Using air instead of O_2_ caused the yield to drop to 17% (Entry 5). Then, we also explored the role of the solvent. Several other polar aprotic solvents, such as DMF and DMA, also afforded the product, albeit in lower yields (entries 6, 7). The use of DCM and MeCN as solvents results in very low reaction yields (Entries 8, 9). When the reaction time was reduced to 24 h, the yield dropped to 68% (Entry 10). This is because the triphenylsilane didn’t react completely. When white light is used as the light source, the reaction cannot proceed at all (Entry 11). Increasing or decreasing the amount of catalyst equivalent decreases the yield of product **2a** (Entry 12, 13).

After obtaining the optimal reaction conditions, we began to explore the substrate scope of this transformation. As summarized in Table 2, generally, the reaction conditions showed good compatibility with diverse silanes and led to the corresponding silanols in moderate to excellent yields. In the beginning, triaryl silanes were explored. For triphenyl silanes, when one phenyl was substituted at para-positions, the corresponding silanols could be obtained in good to excellent yields (2b–2i, 75–94%), regardless of the electron-rich (-Me, -OMe) or electron-deficient (-CF_3_, -CN, -COOEt) properties of the attached substituents. In addition, the meta-substituted triphenyl silanes could also be converted into desired products with good yields (2j–2m, 78–85%). The compatibility with chloride and cyano groups also provided powerful scaffolds to enable further decoration of the obtained silanols. In addition, diphenyl(o-tolyl)silanol 2n was obtained in 82% yield, and [1,1′-biphenyl]-2-yldiphenylsilanol 2o with a sterically bulky group was obtained in 70% yield, demonstrating this protocol was not sensitive to the steric environment of the silicon-centers. In addition to phenyl-substituted silanes, naphthyl substrate also led to high yield (2p, 94%). Heteroaromatic substituents, such as thiophene, dibenzothiophene, and dibenzofuran cycles, had also been found to be compatible with this photooxidation process, resulting in products 2q–2s in 78–90% yields. Finally, methyldiphenyl silane and tert-butyldiphenyl silane were also effective substrates to generate silanols 2t and 2u in 60% and 52% yields, respectively. Dimethyl(phenyl)silanol 2v could also be obtained in a 57% yield.

**Scheme 2:**
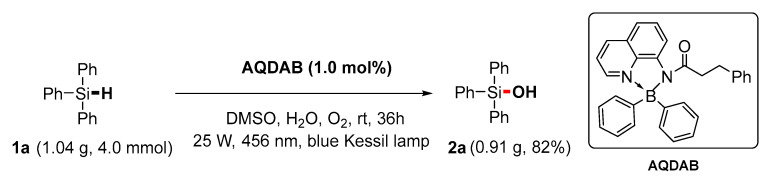
Gram-scale photocatalytic oxidation reaction.

The success of this photooxidation process prompted us to investigate the possibility of a larger-scale synthesis. Delightedly, taking triphenylsilane (1a) as a prototype, the yield of triphenylsilanol (2a) was 82% when the oxidation was performed using gram-scale starting materials (Scheme 2). This could prove the efficiency and demonstrate the application potential of this protocol.

**Scheme 3:**
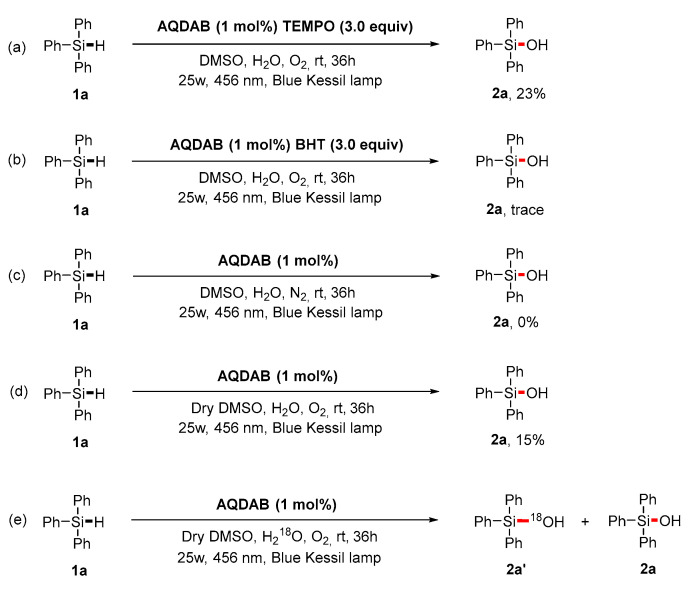
Mechanistic studies. (**a**,**b**) Radical trap experiments. (**c**,**d**) Control experiment. (**e**) ^18^O labeling experiment.

**Figure 1:**
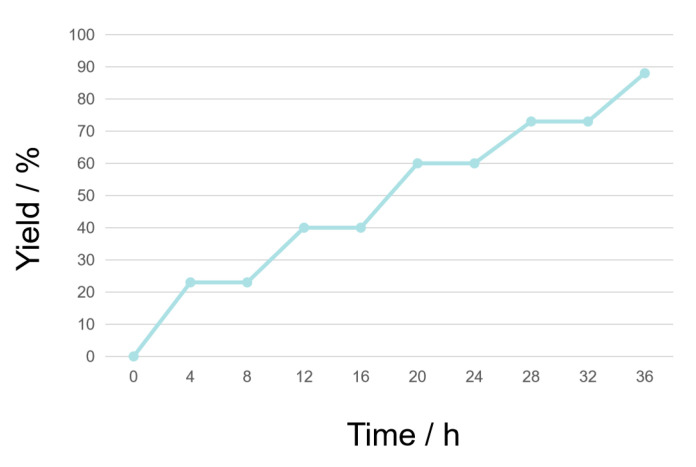
Summary of the on and off reaction of light.

Subsequently, we conducted a series of controlled experiments to elucidate the mechanism of this transformation. First, when the radical quenchers TEMPO (2,2,6,6-tetramethyl-1-piperi-dinyloxy) or BHT (butylated hydroxytoluene) were present in the mixture, the target product **2a** could be obtained in only 23% isolated yield, implying that free radical species might be involved in the reaction pathway (Scheme 3a,b). When the reaction was performed under N_2_, the reaction could not proceed at all (Scheme 3c), indicating oxygen could participate in the reaction. When newly-opened dry DMSO was used as the solvent, the isolated yield decreased to 15% (Scheme 3d), showing H_2_O might also play an important role in the conversion from silanes to silanols. Furthermore, an ^18^O labeling experiment was carried out using triphenylsilane (**1a**) as the substrate with H_2_^18^O under standard conditions (Scheme 3e). HRMS (ESI) analysis [see Supplementary Materials; *m*/*z* calcd for C_18_H_15_^18^OSi^−^ (M − H)^−^ 277.0940, found 277.0938; *m*/*z* calcd for C_18_H_15_OSi^−^ (M − H)^−^ 275.0898, found 275.0900] clearly verified H_2_O and O_2_ as the oxygen sources. Moreover, the on and off reaction of light showed that light was always required to promote the formation of the product during the reaction process (Figure 1).

**Scheme 4:**
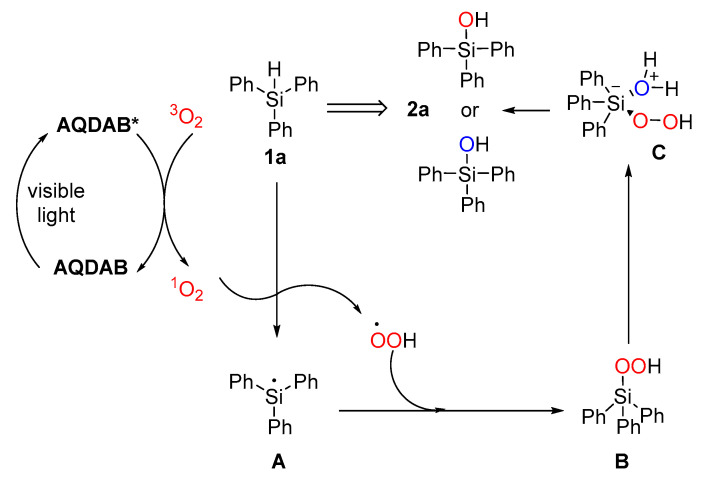
A plausible reaction mechanism.

Based on the above observations and previous reports [43,44], a plausible mechanism for this photooxidation process was proposed, which is shown in Scheme 4. First, **AQADB** was excited to generate AQADB* species under visible light irradiation. Then, AQADB* interacted with ^3^O_2_ to generate ^1^O_2_ through the energy transfer (ET) process. Through this pathway, the excited state of the used photocatalyst AQADB* returned to its ground state. Subsequently, the generated ^1^O_2_ would react directly with silanes **1a**, abstracting a hydrogen atom and forming a transient silil radical **A** plus a hydroperoxy radical HOO^•^. These two radical species would recombine to generate the Si–O bond, leading to the production of silylperoxide **B**. H_2_O might act as a nucleophile to attack silylperoxide **B**, thus forming a pentavalent ate complex **C**, which could decompose into silanol **2**. The proposal that C was involved in was based on the observed different yields between the reactions with or without external water.

## 3. Materials and Methods

### 3.1. Materials and Instruments

Unless otherwise noted, all the reactions of silanes to silanes were carried out under an oxygen atmosphere and a 25 W blue kessil lamp, as well as room temperature. Analytical thin layer chromatography (TLC) was performed on a glass plate uniformly coated with 0.25 mm 230–400 mesh silica gel containing a fluorescence indicator. Visualization was accomplished by exposure to a UV lamp. All the products in this article are compatible with standard silica gel chromatography. Column chromatography was performed on silica gel (200–300 mesh) using standard methods. NMR spectra were measured on a Bruker Ascend 400 spectrometer, and chemical shifts (δ) are reported in parts per million (ppm). ^1^H NMR spectra were recorded at 400 MHz in NMR solvents and referenced internally to the corresponding solvent resonance; ^13^C NMR spectra were recorded at 101 MHz and referenced to the corresponding solvent resonance; ^19^F NMR spectra were recorded at 376 MHz and referenced to corresponding solvent resonance. Coupling constants are reported in Hz, with multiplicities denoted as s (singlet), d (doublet), t (triplet), q (quartet), m (multiplet), and br (broad). Commercial reagents and solvents were purchased from Adamas, J&K, Energy, Sigma-Aldrich, Alfa Aesar, Acros Organics, Innochem, Matrix, Trc, Apinno, Macklin, Ark, Aladdin, Achem-block, Acmec, Coolpharm, Key Organics, and TCI and used as received unless otherwise stated.

### 3.2. General Procedure for the Synthesis of Silanols

A flame-dried 25-mL quartz reaction tube was placed on a magnetic stir bar. Then, silane 1 (0.2 mmol, 1.0 equiv.) were added to the flame-dried 25 mL quartz reaction tube, A triple oxygen replacement process was then performed using a double row of tubes. After that, a mixture of AQDAB (0.9 mg, 0.002 mmol, 1.0 mol%), DMSO (1 mL) and H_2_O (50 μL) was rapidly added into the flame-dried 25 mL quartz reaction tube. The reaction tube was placed on a 25 w blue Kessil reactor. Then the reaction mixture was stirred at 400–500 RPM and exposed to a blue case lamp at room temperature for 36 h. After taking out the reaction tube, transfer the reaction mixture to the separator funnel and add 10 mL of water to the separator funnel. Then, the reaction mixture was extracted with ethyl acetate (3 × 10 mL). The combined organic phase was washed with brine (2 × 5.0 mL) and then dried over anhydrous Na_2_SO_4_. After concentration, the silanol crude product was purified by column chromatography (silica gel) to give silanol **2**, using petroleum ether/ethyl acetate (20:1) as the eluent.

### 3.3. General Procedure for the Synthesis of AQDAB

The preparation methods of photocatalyst AQDAB used in this paper are methods disclosed by us in the previous literature [39,41]. In order to facilitate the synthesis of AQDAB, the preparation process of AQDAB was recorded in detail in this paper. In addition, UV-vis, CV, and fluorescence data are disclosed in the references [39].

#### 3.3.1. Method A for the Synthesis of AQDAB

A flame-dried 25-mL quartz reaction tube was placed on a magnetic stir bar. After that, 3-phenyl-N-(quinolin-8-yl)propanamide (41.4 mg, 0.15 mmol, 1.0 equiv), phenyl trifluoroborate (138.0 mg, 0.75 mmol, 5.0 equiv), Mn (24.7 mg, 0.45 mmol, 3.0 equiv), 4-toluenesulfonyl chloride (71.5 mg, 0.375 mmol, 2.5 equiv.), Na_2_CO_3_ (7.9 mg, 0.075 mmol, 0.5 equiv) and CH_3_CN (1.5 mL) were added. Then the reaction mixture was stirred at 400–500 RPM at 130 °C for 24 h. After concentration, the AQDAB crude product was purified by column chromatography (silica gel) to give 62.7 mg of the photocatalyst in 95% yield, using petroleum ether/ethyl acetate (3:1) as the eluent.

#### 3.3.2. Method B for the Synthesis of AQDAB

A flame-dried 125-mL quartz reaction tube was placed on a magnetic stir bar. Then, 3-phenyl-*N*-(quinolin-8-yl)propanamide (276.3 mg, 1.0 mmol, 1.0 equiv.), phenylboronic acid (1100.0 mg, 9.0 mmol, 9.0 equiv.), K_3_PO_4_ (636.8 mg, 3.0 mmol, 3.0 equiv.) and 1,4-dioxane (15 mL) were added. After that, the reaction mixture was stirred at 300–400 RPM at 130 °C for 36 h. After concentration, the AQDAB crude product was purified by column chromatography (silica gel) to give 286.2 mg of the photocatalyst in 65% yield, using petroleum ether/ethyl acetate (3:1) as the eluent.

#### 3.3.3. Characterization Data of the AQDAB

^1^H NMR (400 MHz, CDCl_3_) δ 8.99 (d, J = 7.6 Hz, 1H), 8.43 (dd, J = 5.2, 0.8 Hz, 1H), 8.38 (d, J = 8.4 Hz, 1H), 7.80 (t, J = 8.4 Hz, 1H), 7.56−7.52 (m, 1H), 7.52−7.46 (m, 5H), 7.30−7.24 (m, 6H), 7.13 (t, J = 7.2 Hz, 2H), 7.10−7.03 (m, 1H), 6.83 (d, J = 6.8 Hz, 2H), 2.60 (dd, J = 9.5, 4.9 Hz, 2H), 2.57−2.49 (m, 2H). ^13^C NMR (101 MHz, CDCl_3_) δ 176.2, 142.0, 141.5, 139.5, 139.1, 137.7, 133.5, 132.6, 128.5, 128.1, 127.9, 127.6, 127.2, 125.5, 122.5, 119.0, 117.2, 39.9, 31.5.

### 3.4. General Procedure for the Synthesis of Starting Materials [45]

#### 3.4.1. The Synthesis of the Starting Materials of **2a–2f**, **2j**, **2k**, **2m–2r**, **2v**

A flame-dried 100-mL round-bottom flask was placed on a magnetic stir bar. Three nitrogen replacement operations were performed on the round-bottomed flask. Then aryl bromide (5.0 mmol, 1.0 equiv.) was dissolved in THF (10 mL) and injected into a round-bottomed flask. After that, the round-bottomed flask is placed on the cryogenic reactor and cooled to −78 °C. (-BuLi (3.2 mL, 1.6 M THF solution, 6.0 mmol, 1.2 equiv.) was slowly injected into a round-bottomed flask over 30 min. The reaction mixture was stirred at 400–500 RPM at −78 °C for 2 h. Then slow injection of chlorodiphenylsilane (6.0 mmol, 1.2 equiv.) into a round-bottomed flask. Heat the reactor to room temperature and stir overnight. The reaction mixture was quenched with NH_4_Cl (15 mL, saturated aqueous solution), and the mixture was extracted with ethyl acetate (3 × 10 mL). The combined organic phase was washed with brine (2 × 5.0 mL) and then dried over anhydrous Na_2_SO_4_. After concentration, the crude product was purified by column chromatography (silica gel) to give silane, using petroleum ether/ethyl acetate (200:1) as the eluent.

#### 3.4.2. The Synthesis of the Starting Materials of **2g–2i**, **2l**

A flame-dried 100-mL round-bottom flask was placed on a magnetic stir bar. Three nitrogen replacement operations were performed on the round-bottomed flask. Then aryl iodide (5.0 mmol, 1.0 equivalent) was dissolved in THF (10 mL) and injected into a round-bottomed flask. After that, the round-bottomed flask is placed on the cryogenic reactor and cooled to −78 °C. i-PrMgCl (3 mL, 2.0 M THF solution, 6.0 mmol, 1.2 equiv.) was slowly injected into a round-bottomed flask over 15 min. The resulting mixture was heated to −40 °C within 2 h and held at −40 °C for another 2 h. Then, slow injection of chlorodiphenylsilane (6.0 mmol, 1.2 equiv.) into a round-bottomed flask. Heat the reactor to room temperature and stir overnight. The reaction mixture was quenched with NH_4_Cl (15 mL, saturated aqueous solution), and the mixture was extracted with CH_2_Cl_2_ (3 × 10 mL). The combined organic phase was washed with brine (2 × 5.0 mL) and then dried over anhydrous Na_2_SO_4_. After concentration, the crude product was purified by column chromatography (silica gel) to give silane, using petroleum ether/ethyl acetate (200:1) as the eluent.

### 3.5. Characterization Data of Products

Triphenylsilanol (**2a**) [45]: Following the General Procedure with triphenylsilane (52.0 mg, 0.2 mmol), **2a** was obtained as colorless oil (48.6 mg, 88%).^1^H NMR (400 MHz, CDCl_3_) *δ* 7.71–7.61 (m, 6H), 7.49–7.44 (m, 3H), 7.43–7.36 (m, 6H), 2.63 (s, 1H).^13^C NMR (101 MHz, CDCl_3_) *δ* 135.1, 135.0, 130.1, 127.9.

Diphenyl(*p*-tolyl)silano (**2b**) [45]: Following the General Procedure with diphenyl(*p*-tolyl)silane (54.8 mg, 0.2 mmol), **2b** was obtained as pale yellow oil (54.5 mg, 94%). ^1^H NMR (400 MHz, CDCl_3_) *δ* 7.62–7.55 (m, 4H), 7.52*–*7.47 (m, 2H), 7.43*–*7.37 (m, 2H), 7.36*–*7.30 (m, 4H), 7.17 (d, *J* = 7.6 Hz, 2H), 2.85 (s, 1H), 2.34 (s, 3H). ^13^C NMR (101 MHz, CDCl_3_) *δ* 140.0, 135.3, 135.0, 134.9, 131.4, 129.9, 128.6, 127.7, 21.5.

(4-(*tert*-butyl)phenyl)diphenylsilanol (**2c**) [45]: Following the General Procedure with (4-(*tert*-butyl)phenyl)diphenylsilane (62.2 mg, 0.2 mmol), **2c** was obtained as colorless oil (59.8 mg, 90%). ^1^H NMR (400 MHz, CDCl_3_) *δ* 7.69*–*7.63 (m, 4H), 7.61*–*7.57 (m, 2H), 7.49*–*7.36 (m, 8H), 2.69 (s, 1H), 1.34 (s, 9H). ^13^C NMR (101 MHz, CDCl_3_) *δ* 153.1, 135.4, 135.0, 134.9, 131.6, 130.0, 127.9, 124.9, 34.8, 31.2.

(4-methoxyphenyl)diphenylsilanol (**2d**) [45]: Following the General Procedure with (4-methoxyphenyl)diphenylsilane (58.0 mg, 0.2 mmol), **2d** was obtained as pale yellow oil (50.0 mg, 82%). ^1^H NMR (400 MHz, CDCl_3_) *δ* 7.63*–*7.58 (m, 4H), 7.54*–*7.50 (m, 2H), 7.39*–*7.32 (m, 6H), 6.93*–*6.87 (m, 2H), 3.79 (s, 3H), 2.66 (s, 1H).^13^C NMR (101 MHz, CDCl_3_) *δ* 161.2, 136.6, 135.5, 134.9, 130.0, 127.9, 126.1, 113.7, 55.0.

[1,1’-biphenyl]-4-yldiphenylsilanol (**2e**) [45]: Following the General Procedure with [1,1*’*-biphenyl]-4-yldiphenylsilane (67.2 mg, 0.2 mmol), **2e** was obtained as white solid (62.7 mg, 89%). ^1^H NMR (400 MHz, CDCl_3_) *δ* 7.76*–*7.68 (m, 6H), 7.67*–*7.61 (m, 4H), 7.53*–*7.46 (m, 4H), 7.46*–*7.36 (m, 5H), 3.08 (s, 1H).^13^C NMR (101 MHz, CDCl_3_) *δ* 142.7, 140.8, 135.5, 135.1, 135.0, 133.8, 130.1, 128.8, 127.9, 127.5, 127.1, 126.6.

2-methyl-4-phenylquinoline (**2f**) [45]: Following the General Procedure with (4-chlorophenyl)diphenylsilane (58.8 mg, 0.2 mmol), **2f** was obtained as pale yellow oil (52.7 mg, 85%). ^1^H NMR (400 MHz, CDCl_3_) δ 7.64–7.57 (m, 4H), 7.56–7.44 (m, 4H), 7.42–7.33 (m, 6H), 3.16 (s, 1H).^13^C NMR (101 MHz, CDCl_3_) δ 136.5, 136.3, 134.9, 134.6, 133.5, 130.2, 128.1, 128.0.

Diphenyl(4-(trifluoromethyl)phenyl)silanol (**2g**) [45]: Following the General Procedure with diphenyl(4-(trifluoromethyl)phenyl)silane (65.6 mg, 0.2 mmol), **2g** was obtained as pale yellow oil (51.6 mg, 75%). ^1^H NMR (400 MHz, CDCl_3_) δ 7.75 (d, J = 7.6 Hz, 2H), 7.65–7.57 (m, 6H), 7.51–7.45 (m, 2H), 7.44–7.36 (m, 4H), 2.92 (s, 1H).^13^C NMR (101 MHz, CDCl3) δ 140.1, 135.2, 134.9, 134.2, 131.9 (q, J = 32.2 Hz), 130.5, 128.1, 124.4 (q, J = 3.7 Hz), 124.1 (q, J = 273.7 Hz). ^19^F NMR (376 MHz, CDCl_3_) δ −62.91.

Ethyl 4-(hydroxydiphenylsilyl)benzoate (**2h**) [45]: Following the General Procedure with ethyl 4-(diphenylsilyl)benzoate (66.4 mg, 0.2 mmol), **2h** was obtained as colorless oil (59.2 mg, 85%). ^1^H NMR (400 MHz, CDCl_3_) δ 8.04–7.96 (m, 2H), 7.73–7.68 (m, 2H), 7.65–7.58 (m, 4H), 7.48–7.35 (m, 6H), 4.36 (q, J = 7.2 Hz, 2H), 3.59 (s, 1H), 1.38 (t, J = 6.8 Hz, 3H). ^13^C NMR (101 MHz, CDCl_3_) δ 166.8, 141.3, 134.90, 134.88, 134.6, 131.5, 130.2, 128.5, 127.9, 61.1, 14.2.

4-(hydroxydiphenylsilyl)benzonitrile (**2i**) [45]: Following the General Procedure with methyl 4-(diphenylsilyl)benzonitrile (57.0 mg, 0.2 mmol), **2i** was obtained as colorless oil (51.78 mg, 86%). ^1^H NMR (400 MHz, CDCl_3_) δ 7.70–7.69 (m, 2H), 7.64–7.54 (m, 6H), 7.51–7.25 (m, 2H), 7.43–7.36 (m, 4H), 3.38 (s, 1H). ^13^C NMR (101 MHz, CDCl_3_) δ 142.1, 135.3, 134.8, 133.8, 131.0, 130.5, 128.1, 118.7, 113.3.

Diphenyl(m-tolyl)silanol (**2j**) [45]: Following the General Procedure with diphenyl(m-tolyl)silane (54.8 mg, 0.2 mmol), **2j** was obtained as white solid (45.3 mg, 78%). ^1^H NMR (400 MHz, CDCl_3_) δ 7.63–7.58 (m, 4H), 7.46–7.39 (m, 4H), 7.38–7.32 (m, 4H), 7.28–7.22 (m, 2H), 2.76 (s, 1H), 2.31 (s, 3H). ^13^C NMR (101 MHz, CDCl_3_) δ 137.3, 135.4, 135.3, 135.0, 132.1, 130.9, 130.0, 127.9, 127.8, 21.5.

(4-methoxyphenyl)diphenylsilanol (**2k**) [34]: Following the General Procedure with (3-methoxyphenyl)diphenylsilane (58.0 mg, 0.2 mmol), **2k** was obtained as white solid (52.0 mg, 85%). ^1^H NMR (400 MHz, CDCl_3_) δ 7.67–7.58 (m, 4H), 7.45–7.40 (m, 2H), 7.39–7.34 (m, 4H), 7.33–7.28 (m, 1H), 7.21–7.12 (m, 2H), 7.00–6.93 (m, 1H), 3.75 (s, 3H), 2.61 (s, 1H). ^13^C NMR (101 MHz, CDCl_3_) δ 159.0, 136.7, 135.00, 134.95, 130.1, 129.2, 127.9, 127.3, 120.1, 115.7, 55.1.

(3-chlorophenyl)diphenylsilanol (**2l**) [34]: Following the General Procedure with (3-chlorophenyl)diphenylsilane (58.8 mg, 0.2 mmol), **2l** was obtained as colorless oil (50.2 mg, 81%). ^1^H NMR (400 MHz, CDCl_3_) δ 7.65–7.58 (m, 5H), 7.52–7.44 (m, 3H), 7.44–7.38 (m, 5H), 7.35–7.30 (m, 1H), 2.62 (s, 1H). ^13^C NMR (101 MHz, CDCl_3_) δ 137.9, 134.9, 134.5, 134.4, 134.3, 134.2, 132.9, 130.4, 130.2, 129.4, 128.1.

(3-fluorophenyl)diphenylsilanol (**2m**) [45]: Following the General Procedure with (3-fluorophenyl)diphenylsilane (55.6 mg, 0.2 mmol), **2m** was obtained as white solid (50.0 mg, 85%). ^1^H NMR (400 MHz, CDCl_3_) δ 7.66–7.59 (m, 4H), 7.50–7.44 (m, 2H), 7.43–7.31 (m, 7H), 7.17–7.10 (m, 1H), 2.97 (s, 1H).^13^C NMR (101 MHz, CDCl_3_) δ 162.5 (d, J = 248.6 Hz), 138.3 (d, J = 4.2 Hz), 134.9, 134.4, 130.5 (d, J = 3.1 Hz), 130.3, 129.8 (d, J = 6.9 Hz), 128.0, 121.3 (d, J = 19.1 Hz), 117.1 (d, J = 21.1 Hz). ^19^F NMR (376 MHz, CDCl_3_) δ −113.04.

Diphenyl(o-tolyl)silanol (**2n**) [45]: Following the General Procedure with diphenyl(o-tolyl)silane (54.8 mg, 0.2 mmol), **2n** was obtained as pale yellow oil (47.6 mg, 82%). ^1^H NMR (400 MHz, CDCl_3_) δ 7.61–7.54 (m, 4H), 7.46–7.39 (m, 3H), 7.38–7.31 (m, 5H), 7.20–7.11 (m, 2H), 2.65 (s, 1H), 2.30 (s, 3H). ^13^C NMR (101 MHz, CDCl_3_) δ 144.6, 136.6, 135.6, 134.9, 133.5, 130.4, 130.0, 129.9 127.9, 124.8, 23.3.

[1,1′-biphenyl]-2-yldiphenylsilanol (**2o**) [37]: Following the General Procedure with [1,1’-biphenyl]-2-yldiphenylsilane (67.2 mg, 0.2 mmol), **2o** was obtained as white solid (49.3 mg, 70%).^1^H NMR (400 MHz, CDCl_3_) δ 7.51–7.44 (m, 6H), 7.42–7.37 (m, 2H), 7.36–7.30 (m, 6H), 7.28–7.20 (m, 3H), 7.19–7.14 (m, 2H).^13^C NMR (101 MHz, CDCl_3_) δ 149.0, 143.6, 136.6, 136.4, 134.8, 134.1, 129.8, 129.72, 129.69, 129.0, 128.2, 127.8, 127.5, 126.3.

Naphthalen-1-yldiphenylsilanol (**2p**) [45]: Following the General Procedure with naphthalen-1-yldiphenylsilane (62.0 mg, 0.2 mmol), **2p** was obtained as pale yellow oil (61.3 mg, 94%). ^1^H NMR (400 MHz, CDCl_3_) δ 8.17 (d, J = 8.4 Hz, 1H), 7.97 (d, J = 8.0 Hz, 1H), 7.90 (d, J = 8.0 Hz, 1H), 7.72–7.63 (m, 5H), 7.49–7.36 (m, 9H), 2.84 (s, 1H). ^13^C NMR (101 MHz, CDCl_3_) δ 137.1, 136.5, 135.7, 135.0, 133.4, 132.9, 131.1, 130.1, 128.9, 128.8, 128.0, 126.1, 125.6, 125.0.

Diphenyl(thiophen-2-yl)silanol (**2q**) [45]: Following the General Procedure with diphenyl(thiophen-2-yl)silane (53.2 mg, 0.2 mmol), **2q** was obtained as colorless oil (44.0 mg, 78%). ^1^H NMR (400 MHz, CDCl_3_) δ 7.71–7.54 (m, 5H), 7.44–7.24 (m, 7H), 7.22–7.14 (m, 1H), 3.06 (s, 1H). ^13^C NMR (101 MHz, CDCl_3_) δ 137.6, 134.9, 134.7, 134.4, 132.5, 130.3, 128.3, 127.9.

Dibenzo[b,d]thiophen-4-yldiphenylsilanol (**2r**) [45]: Following the General Procedure with dibenzo[b,d]thiophen-4-yldiphenylsilane (73.2 mg, 0.2 mmol), **2r** was obtained as white solid (62.7 mg, 82%). ^1^H NMR (400 MHz, CDCl_3_) δ 8.25 (d, J = 8.0 Hz, 1H), 8.20–8.15 (m, 1H), 7.77–7.69 (m, 5H), 7.65 (d, J = 7.2 Hz, 1H), 7.52–7.39 (m, 9H), 3.07 (s, 1H). ^13^C NMR (101 MHz, CDCl_3_) δ 145.8, 139.7, 135.2, 134.9, 134.8 134.0, 130.4, 129.4, 128.0, 126.6, 124.2, 123.8, 123.5, 122.6, 121.4.

Dibenzo[b,d]furan-4-yldiphenylsilanol (**2s**) [45]: Following the General Procedure with dibenzo[b,d]furan-4-yldiphenylsilane (70.0 mg, 0.2 mmol), **2s** was obtained as white solid (65.9 mg, 90%). ^1^H NMR (400 MHz, CDCl_3_) δ 8.07 (d, J = 7.6 Hz, 1H), 7.99 (d, J = 7.6 Hz, 1H), 7.74 (d, J = 8.0 Hz, 4H), 7.56–7.35 (m, 11H), 3.41 (s, 1H).^13^C NMR (101 MHz, CDCl_3_) δ 160.8, 155.9, 135.0, 134.7, 134.2, 130.3, 127.9, 127.1, 123.9, 123.1, 122.8, 122.77, 122.69, 120.6, 118.2, 111.8.

Methyldiphenylsilanol (**2t**) [45]: Following the General Procedure with methyldiphenylsilane (39.6 mg, 0.2 mmol), **2t** was obtained as colorless oil (25.7 mg, 60%). ^1^H NMR (400 MHz, CDCl_3_) δ 7.64–7.60 (m, 4H), 7.44–7.38 (m, 6H), 2.40 (s, 1H), 0.68 (s, 3H). ^13^C NMR (101 MHz, CDCl_3_) δ 137.0, 133.9, 129.9, 127.9, −1.3.

Tert-butyldiphenylsilanol (**2u**) [37]: Following the General Procedure with tert-butyldiphenylsilane (48.0 mg, 0.2 mmol), **2t** was obtained as colorless oil (26.6 mg, 52%). ^1^H NMR (400 MHz, CDCl_3_) δ 7.77–7.70 (m, 4H), 7.44–7.36 (m, 6H), 2.17 (s, 1H), 1.08 (s, 9H). ^13^C NMR (101 MHz, CDCl_3_) δ 135.1, 134.8, 129.6, 127.7, 26.5, 19.0.

Dimethyl(phenyl)silanol (**2v**) [45]: Following the General Procedure with dimethyl(phenyl)silane (27.2 mg, 0.2 mmol), **2u** was obtained as colorless oil (17.3 mg, 57%). ^1^H NMR (400 MHz, CDCl_3_) δ 7.63–7.58 (m, 2H), 7.42–7.36 (m, 3H), 1.90 (s, 1H), 0.41 (s, 6H). ^13^C NMR (101 MHz, CDCl_3_) δ 139.1, 133.0, 129.6, 127.9, 0.0.

## 4. Conclusions

In conclusion, we have developed a photocatalytic oxidation strategy to achieve silanol synthesis. Four-coordinate aminoquinolate diarylboron compounds are used as photocatalysts for this conversion, which can produce ^1^O under visible light irradiation. This transformation bypasses the use of noble metal-based photocatalysts or oxidants. The boron-based photocatalyst is demonstrated herein to be a sustainable supplement to the noble metal-based photocatalysts. Research on its further application is also underway in our laboratory.

## Figures and Tables

**Table 1:** Optimization of the reaction conditions ^(a)^.

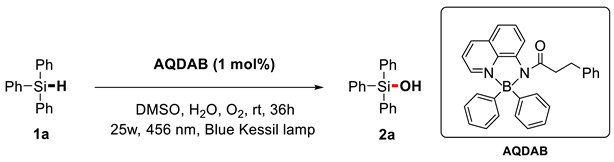		
Entry	Variations from the Standard Conditions	Yield ^(b)^
1	None	88%
2	without AQDAB	N.R.
3	without O_2_ (under N_2_)	N.R.
4	without Kessil lamp	N.R.
5	air instead of O_2_	17%
6	DMA instead of DMSO	20%
7	DMF instead of DMSO	28%
8	MeCN instead of DMSO	trace
9	DCM instead of DMSO	trace
10	reaction time: 24 h	68%
11	white Kessil lamp	N.R.
12	AQDAB (0.5 mol%)	85%
13	AQDAB (2 mol%)	87%

^(a)^ Standard conditions: **1a** (0.20 mmol), AQDAB (1.0% mol), O_2_ (1 atm), light source, DMSO/H_2_O (1.0 mL/50 μL), rt, 36 h; ^(b)^ Isolated yields. N.R.—no reaction.

**Table 2:** Substrate scope of silanes.

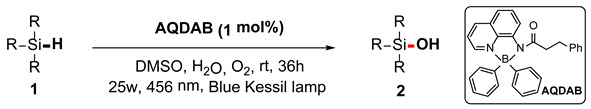		
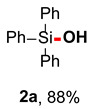	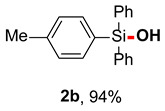	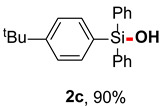
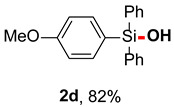	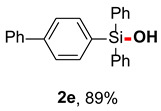	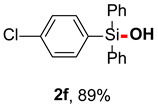
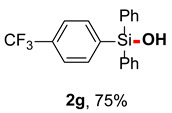	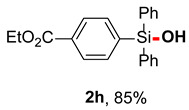	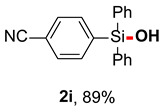
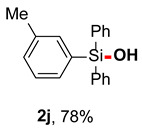	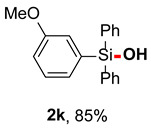	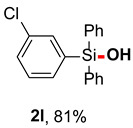
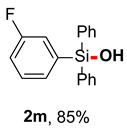	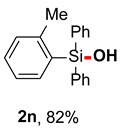	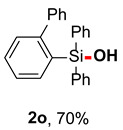
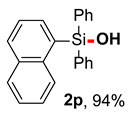	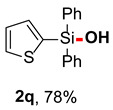	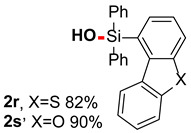
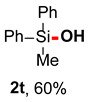	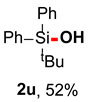	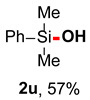

Reaction conditions: **1a** (0.20 mmol), AQDAB (1.0% mol), O_2_ (1 atm), light source, DMSO/H_2_O (1.0 mL/50 μL), rt, 36 h, isolated yields.

## Data Availability

The data presented in this study are available in the article and Supplementary Materials.

## Supplementary Materials

The following supporting information can be downloaded at: https://www.mdpi.com/article/10.3390/molecules28104082/s1, copies of 1H-NMR, 13C-NMR, and 19F-NMR spectra of the products are included in the Supplementary Materials.

## Sample Availability

Samples of the compounds **2a**–**2v** are available from the authors.
