# Supplementary material for: Visible Light-Mediated Organoboron-Catalyzed Metal-Free Synthesis of Silanols from Silanes
Source: Molecules. 2023 May 13;28(10):4082. doi: 10.3390/molecules28104082 (PMC10222655; doi:10.3390/molecules28104082)
Supplement: Supplementary file 1 [file molecules-28-04082-s001.zip › molecules-2370397-supplementary.pdf]

Supplementary Materials

Visible Light-Mediated Organoboron-Catalyzed Metal-Free Synthesis of Silanols from Silanes

Jinbo Yang [1], Xiangxue Cao [1], Lanfeng Wei [1], Jianshu Zhang [1], Jinli Zhang [2], Ping Liu [1], Liang Xu [1,*] and Pengfei Li [3,*]

[1] State Key Laboratory Incubation Base for Green Processing of Chemical Engineering, School of Chemistry and Chemical Engineering, Shihezi University, Beisi Road, Xinjiang Uygur Autonomous region, Shihezi 832003, China; yangjinbo@stu.shzu.edu.cn (J.Y.); caoxiangxue@stu.shzu.edu.cn (X.C.); weilanfeng@stu.shzu.edu.cn (L.W.); zhangjs@shzu.edu.cn (J.Z.); liuping1979112@aliyun.com (P.L.)

[2] School of Chemical Engineering and Technology, Tianjin University, Tianjin 300072, China; zhangjinli@tju.edu.cn

[3] State Key Laboratory for Mechanical Behavior of Materials, Frontier Institute of Science and Technology, Xi'an Jiaotong University, Xi'an 710054, China

* Correspondence: xuliang4423@shzu.edu.cn (L.X.); lipengfei@xjtu.edu.cn (P.L.)

Contents

1. ^{18}O Labelling experiments 3

2. Copies of NMR spectra 4

1. ^{18}O Labelling experiments

flame-dried 25 mL quartz reaction tube was placed with a magnetic stir bar. Then, silane 1 (0.2 mmol, 1.0 equiv)were added to the flame-dried 25 mL quartz reaction tube, A triple oxygen replacement process was then performed using a double row of tubes.. After that, A mixture of AQDAB (0.9 mg, 0.002 mmol, 1.0 mol%), Dry DMSO (1 ml) and $H_2{}^{18}O$ (50 μL) was rapidly added into the flame-dried 25 mL quartz reaction tube. The reaction tube was placed on a 25w blue Kessil reactor. Then the reaction mixture was stirred at 400-500 RPM and exposed to a blue case lamp at room temperature for 36 hours. After taking out the reaction tube, transfer the reaction mixture to the separator funnel and add 10 ml water to the separator funnel. Then, the reaction mixture was extracted with ethyl acetate (3 × 10 mL). The combined organic phase was washed with brine (2 × 5.0 mL) and then dried over anhydrous Na_2SO_4. After concentration, the silanol crude product was purified by column chromatography (silica gel) to give silanol 2, using petroleum ether/ethyl acetate (20 : 1) as the eluent.

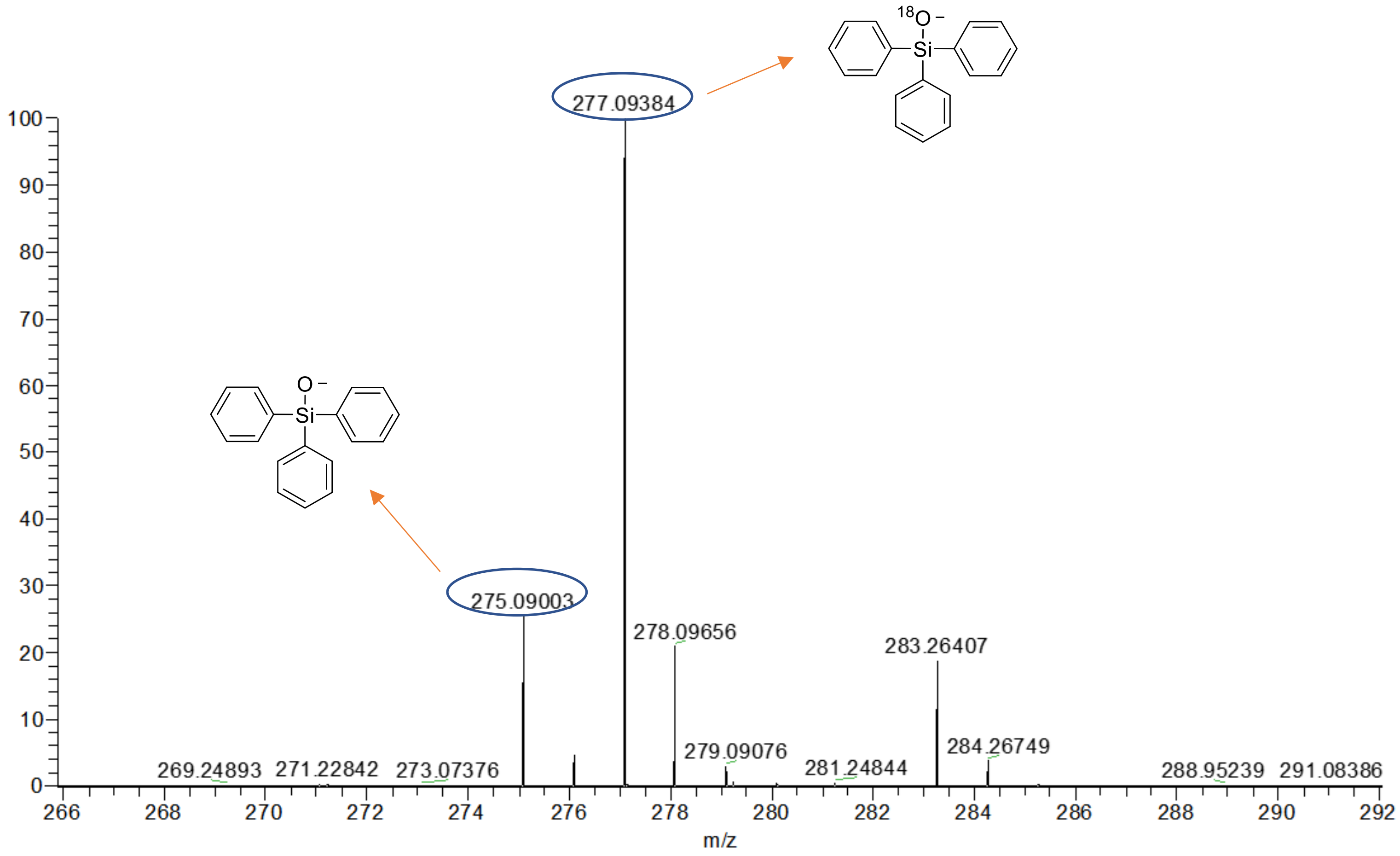

2. Copies of NMR spectra

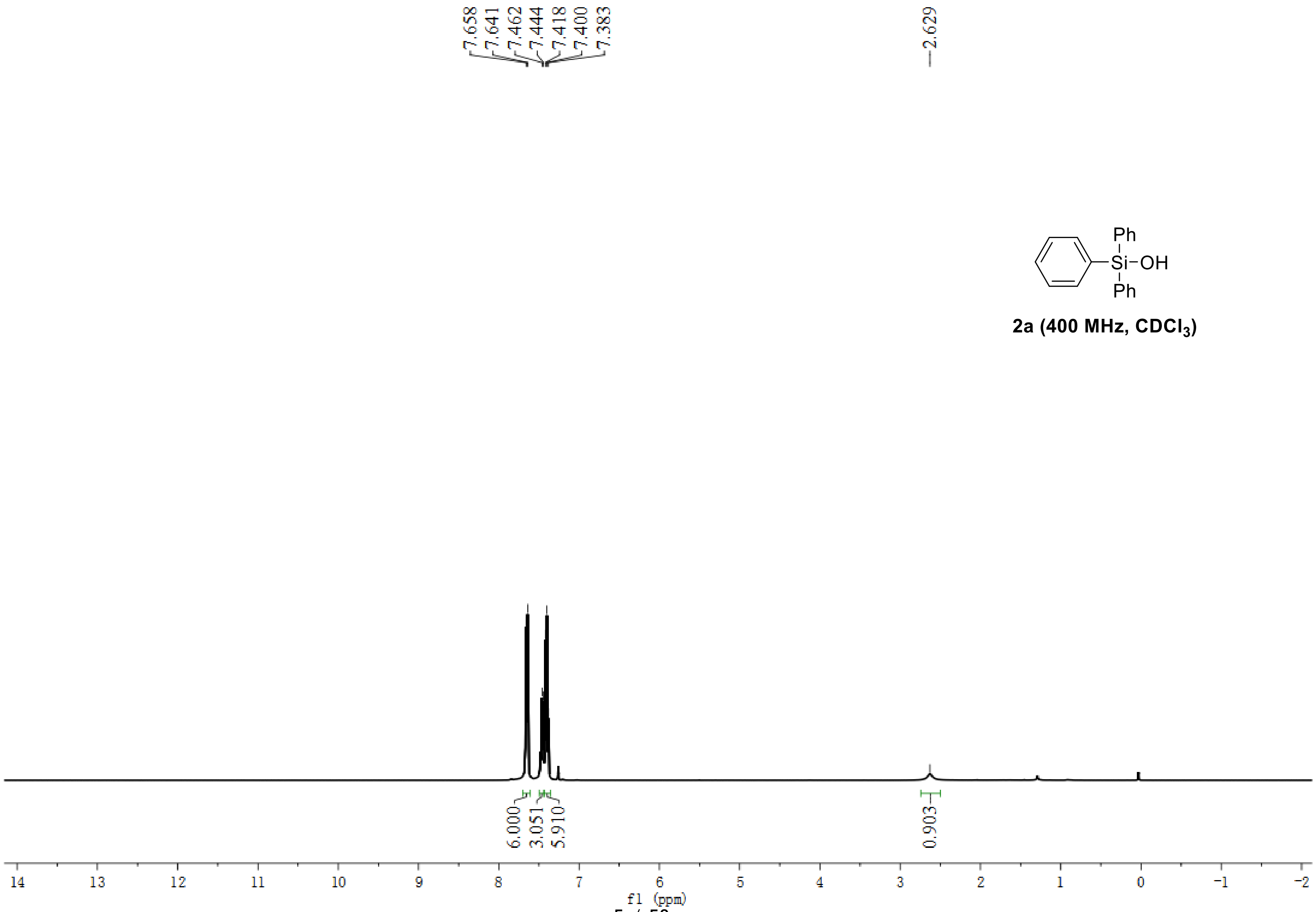
7.658
7.641
7.462
7.444
7.418
7.400
7.383
2.629
Ph
Si
OH
Ph
2a (400 MHz, CDCl3)
6.000
3.051
5.910
0.903
14
13
12
11
10
9
8
7
6
5
4
3
2
1
0
-1
-2
f1 (ppm)

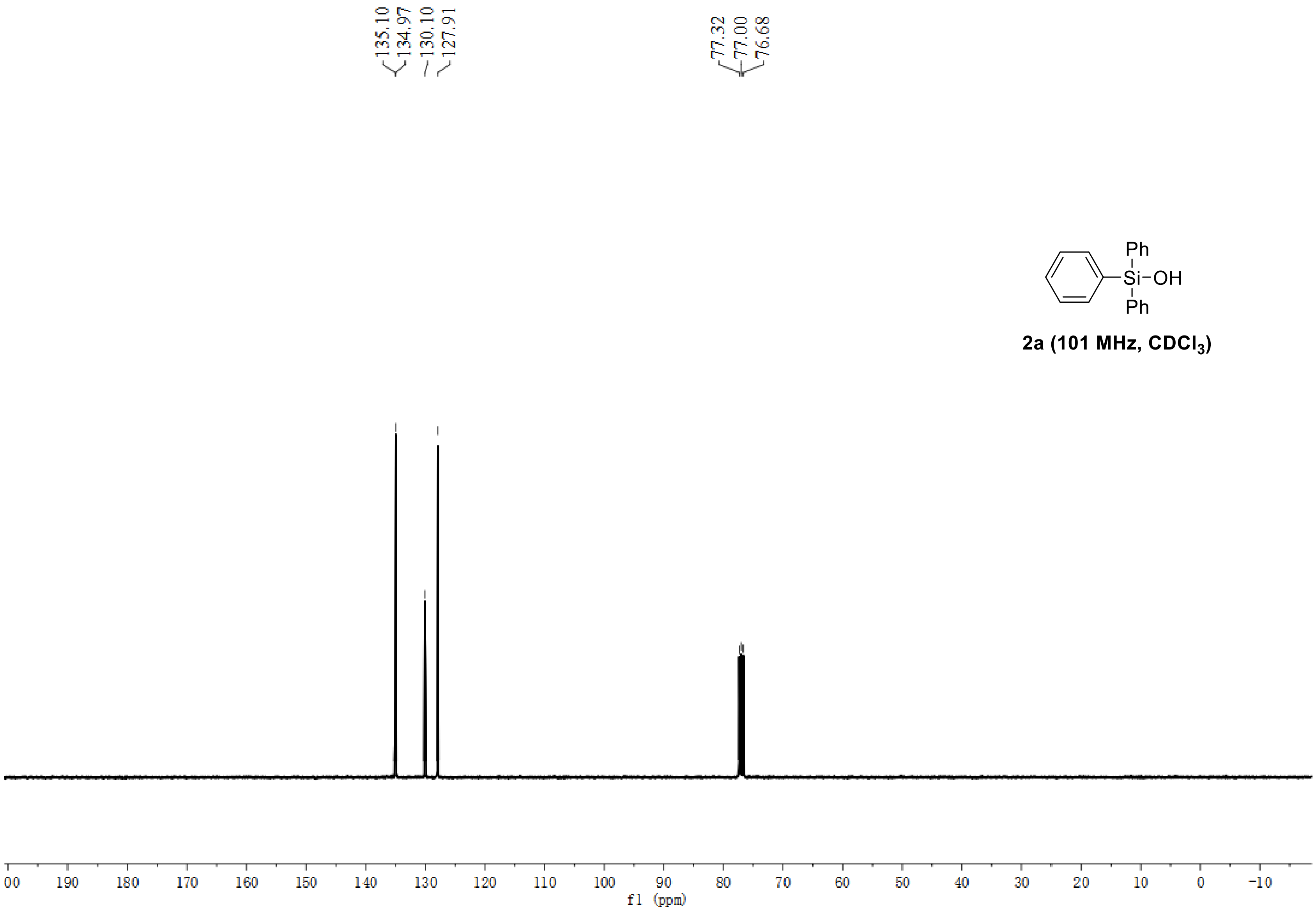

2a (101 MHz, $CDCl_3$)

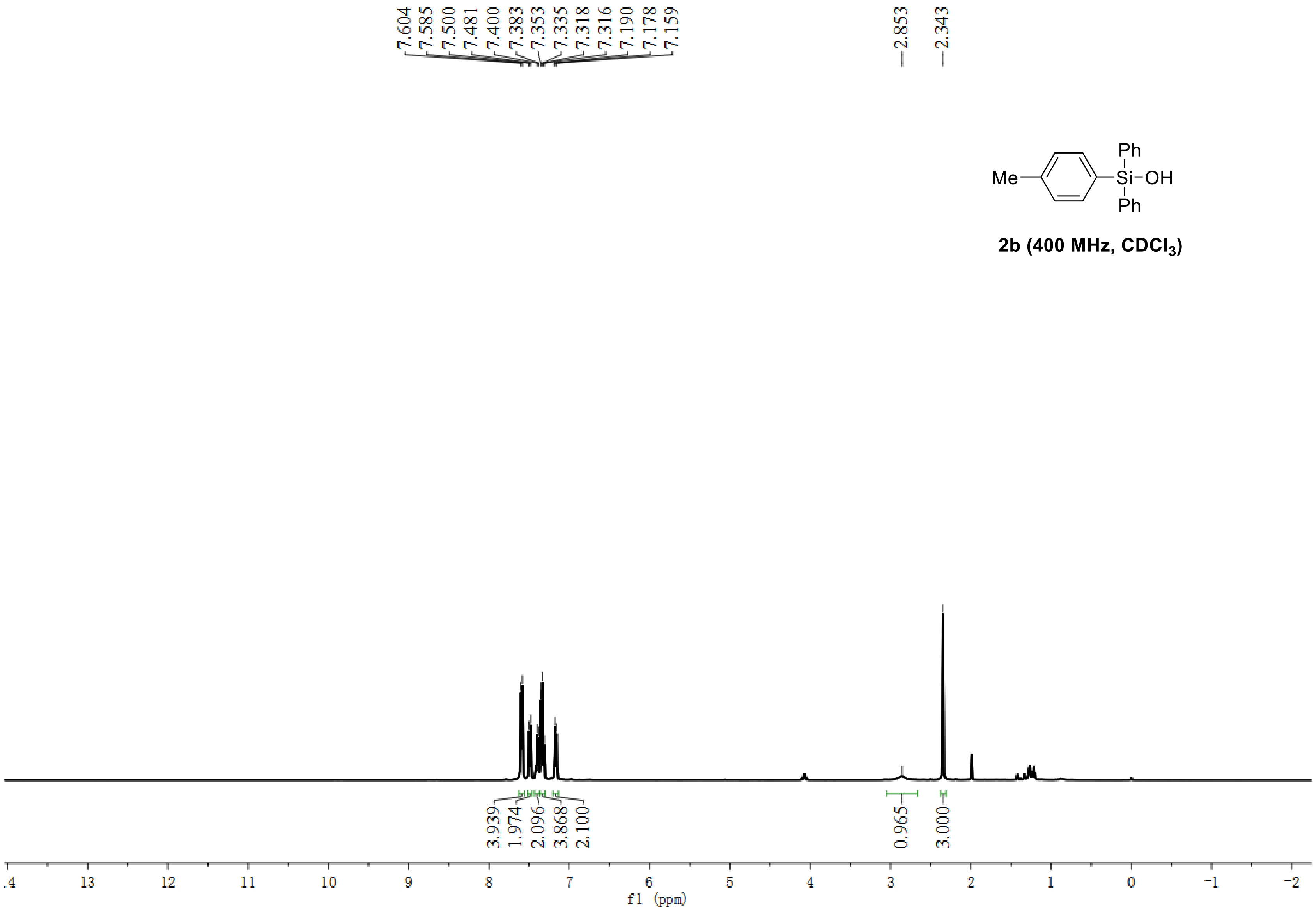
7.604
7.585
7.500
7.481
7.400
7.383
7.353
7.335
7.318
7.316
7.190
7.178
7.159
2.853
2.343
Me
Ph
Si
OH
Ph
2b (400 MHz, CDCl3)
3.939
1.974
2.096
3.868
2.100
0.965
3.000
f1 (ppm)

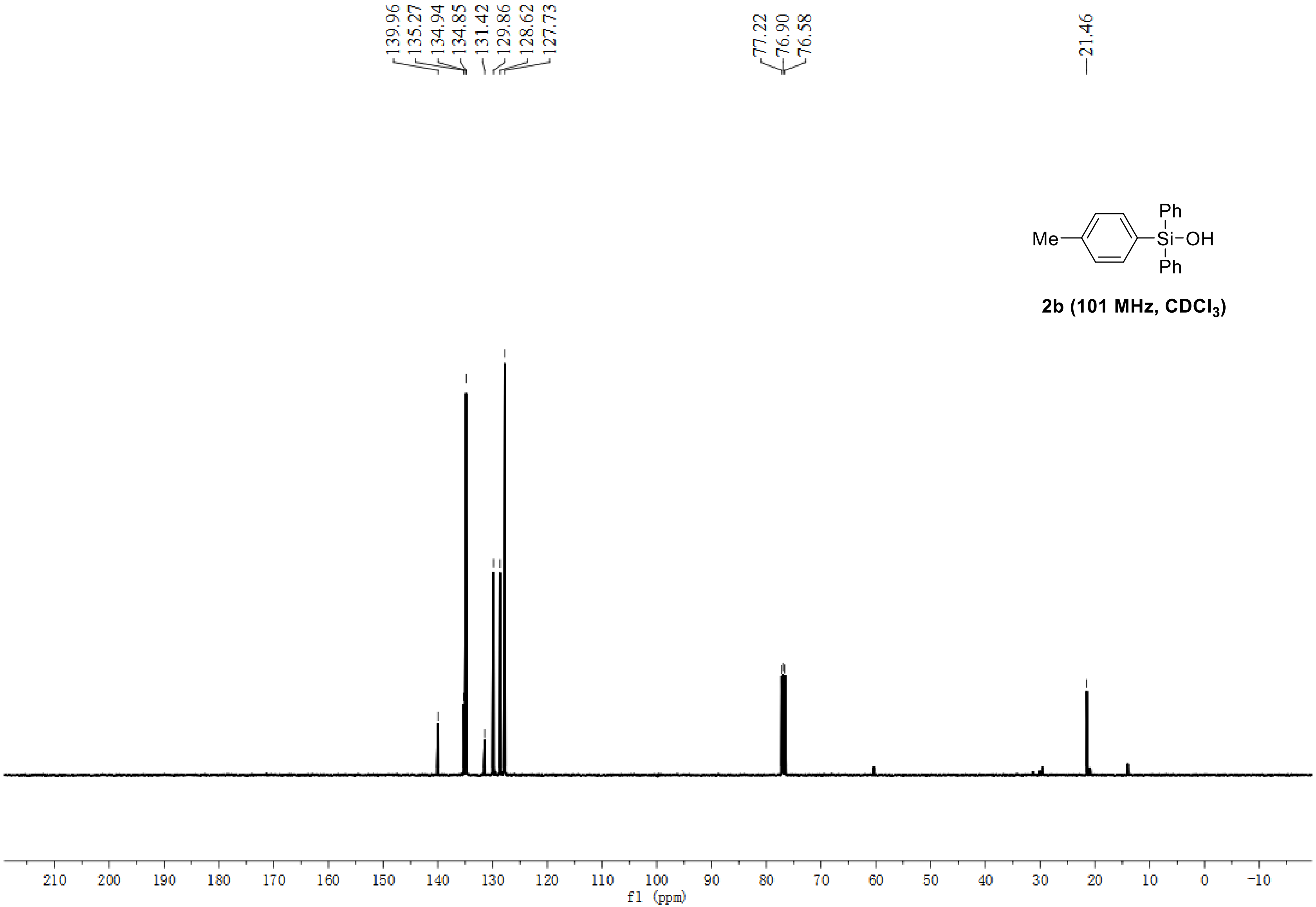

2b (101 MHz, $CDCl_3$)

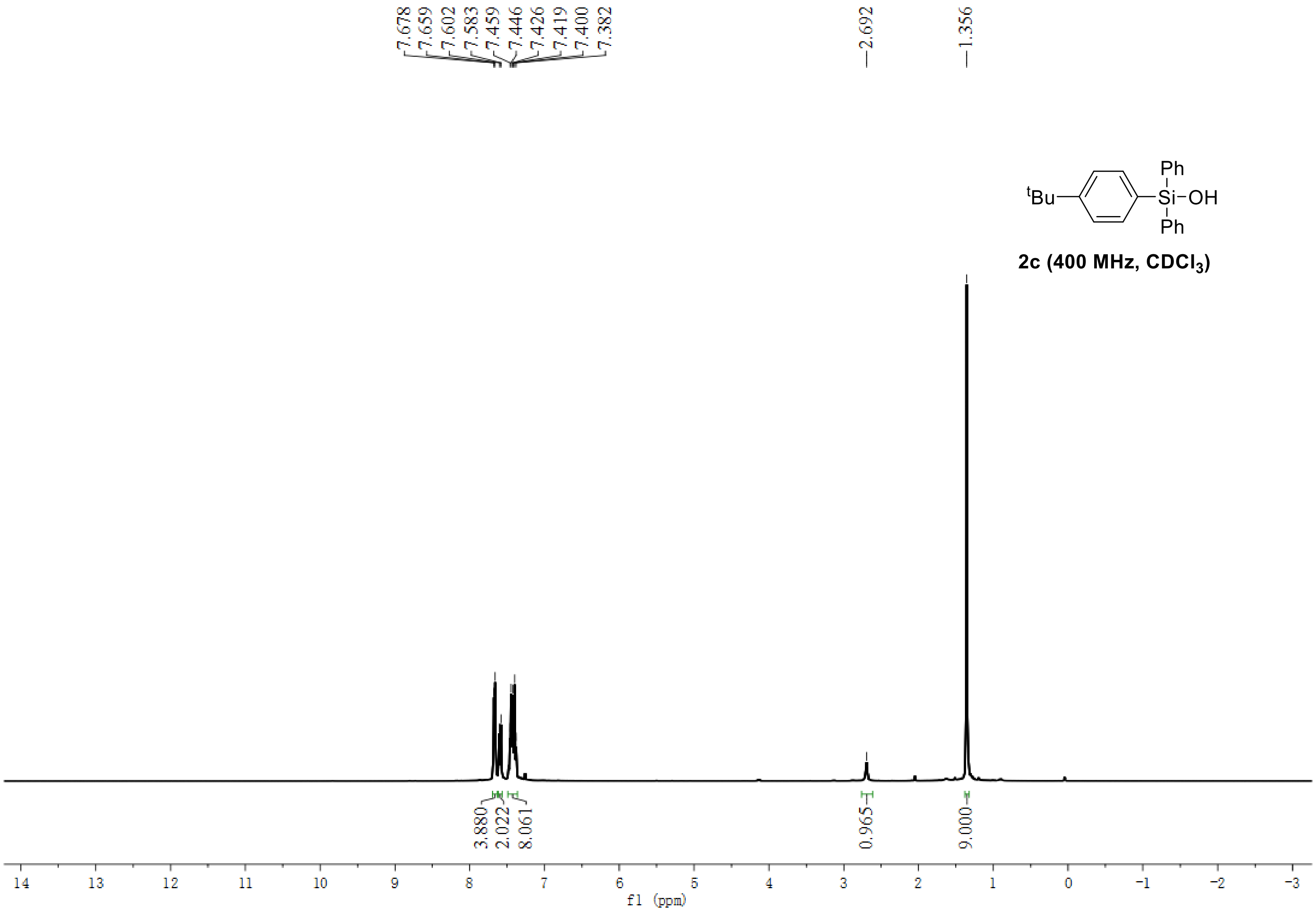

2c (400 MHz, $CDCl_3$)

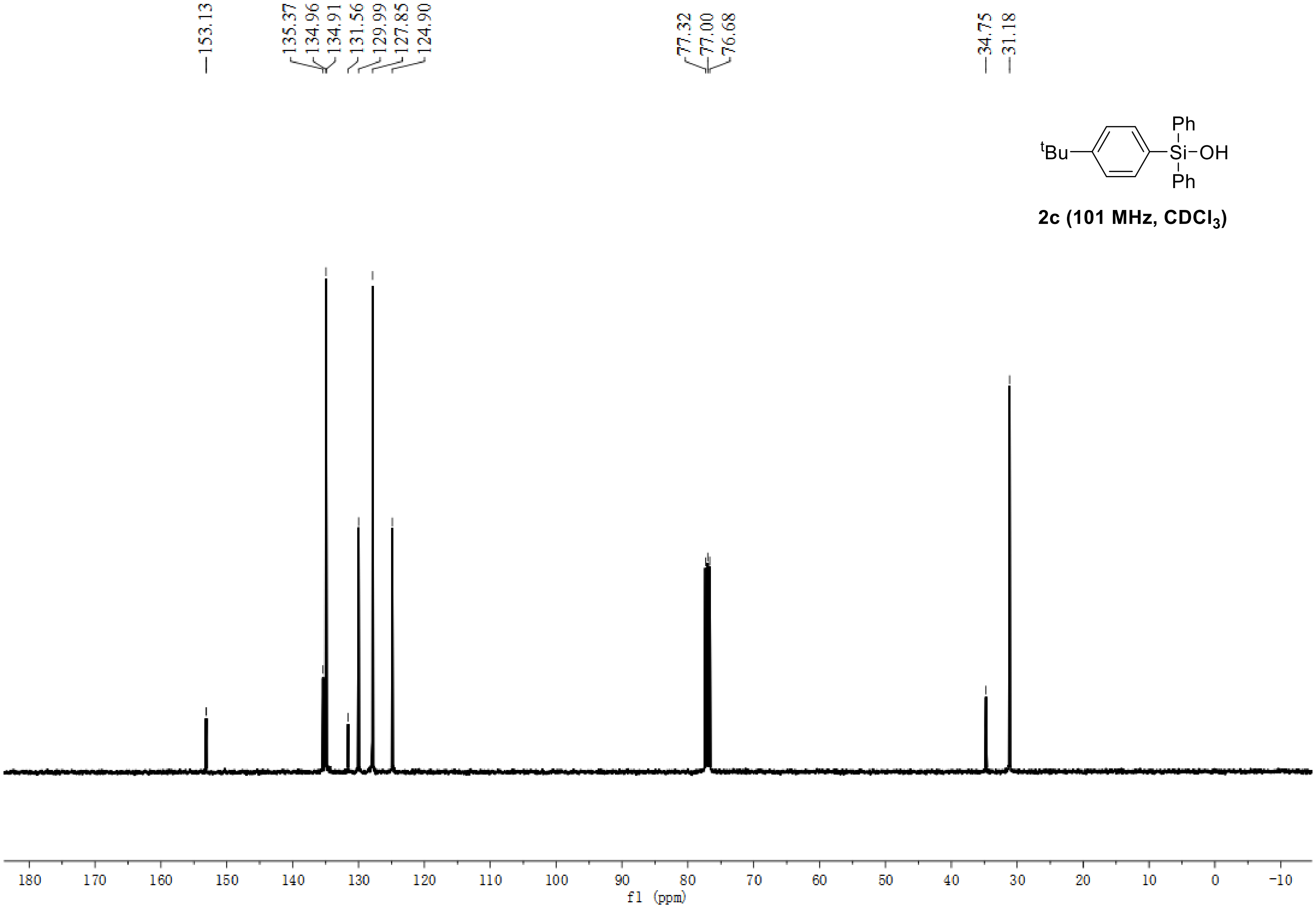
153.13
135.37
134.96
134.91
131.56
129.99
127.85
124.90
77.32
77.00
76.68
34.75
31.18
Ph
^{t}Bu
Si–OH
Ph
2c (101 MHz, $CDCl_3$)
180 170 160 150 140 130 120 110 100 90 80 70 60 50 40 30 20 10 0 −10
f1 (ppm)

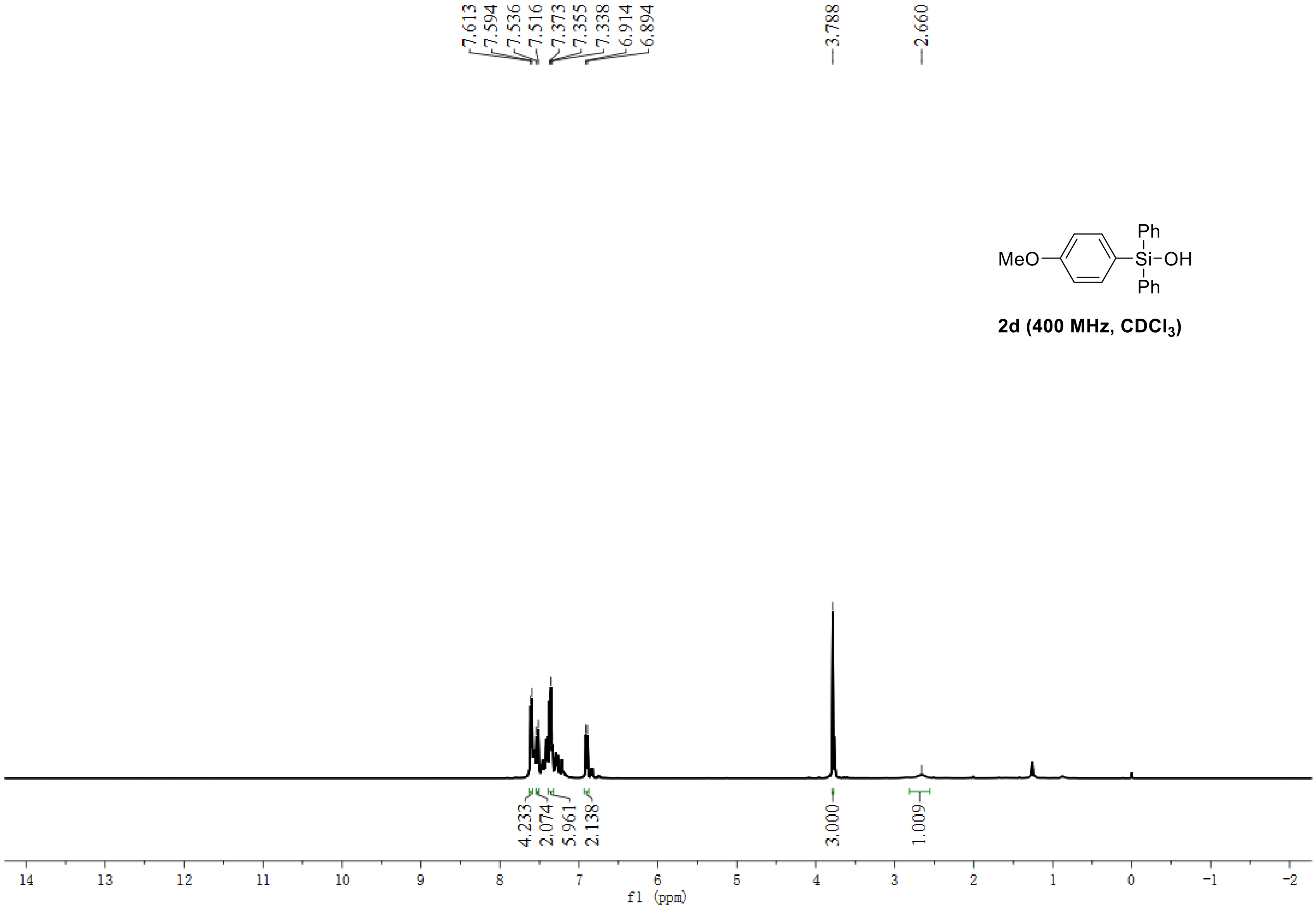

2d (400 MHz, $CDCl_3$)

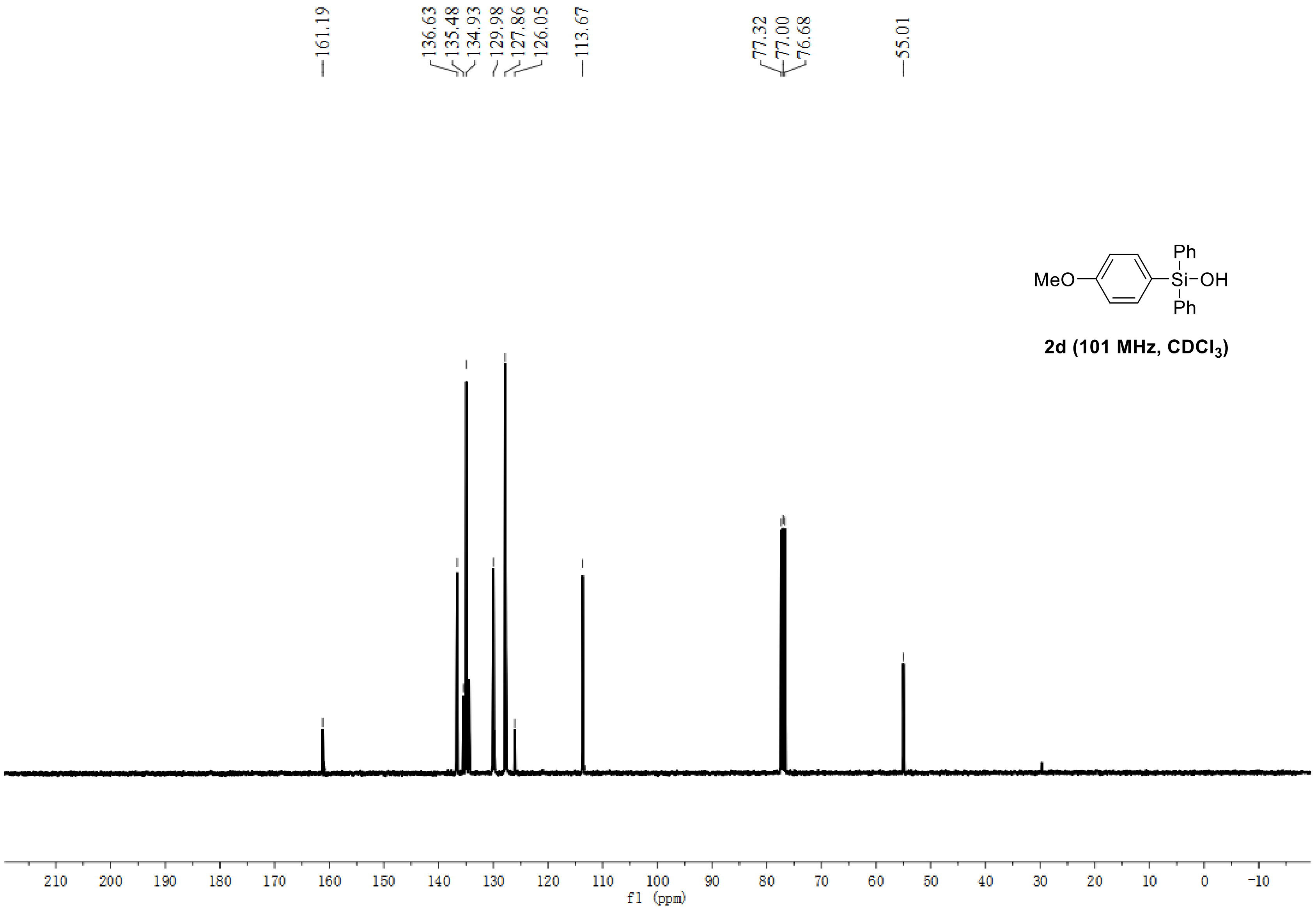
161.19
136.63
135.48
134.93
129.98
127.86
126.05
113.67
77.32
77.00
76.68
55.01
MeO
Ph
Si
OH
Ph
2d (101 MHz, CDCl3)
210 200 190 180 170 160 150 140 130 120 110 100 90 80 70 60 50 40 30 20 10 0 -10
f1 (ppm)

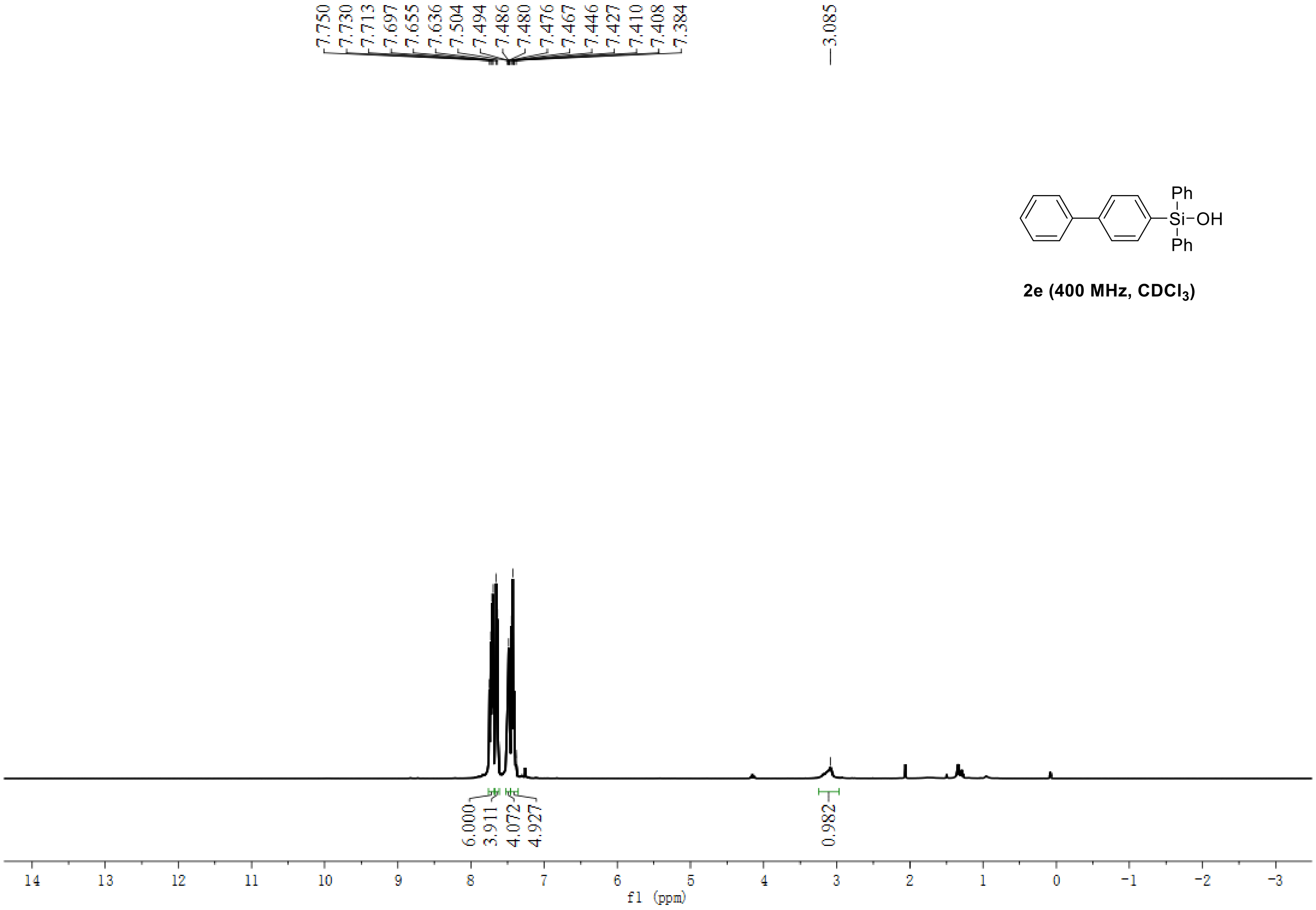

2e (400 MHz, $CDCl_3$)

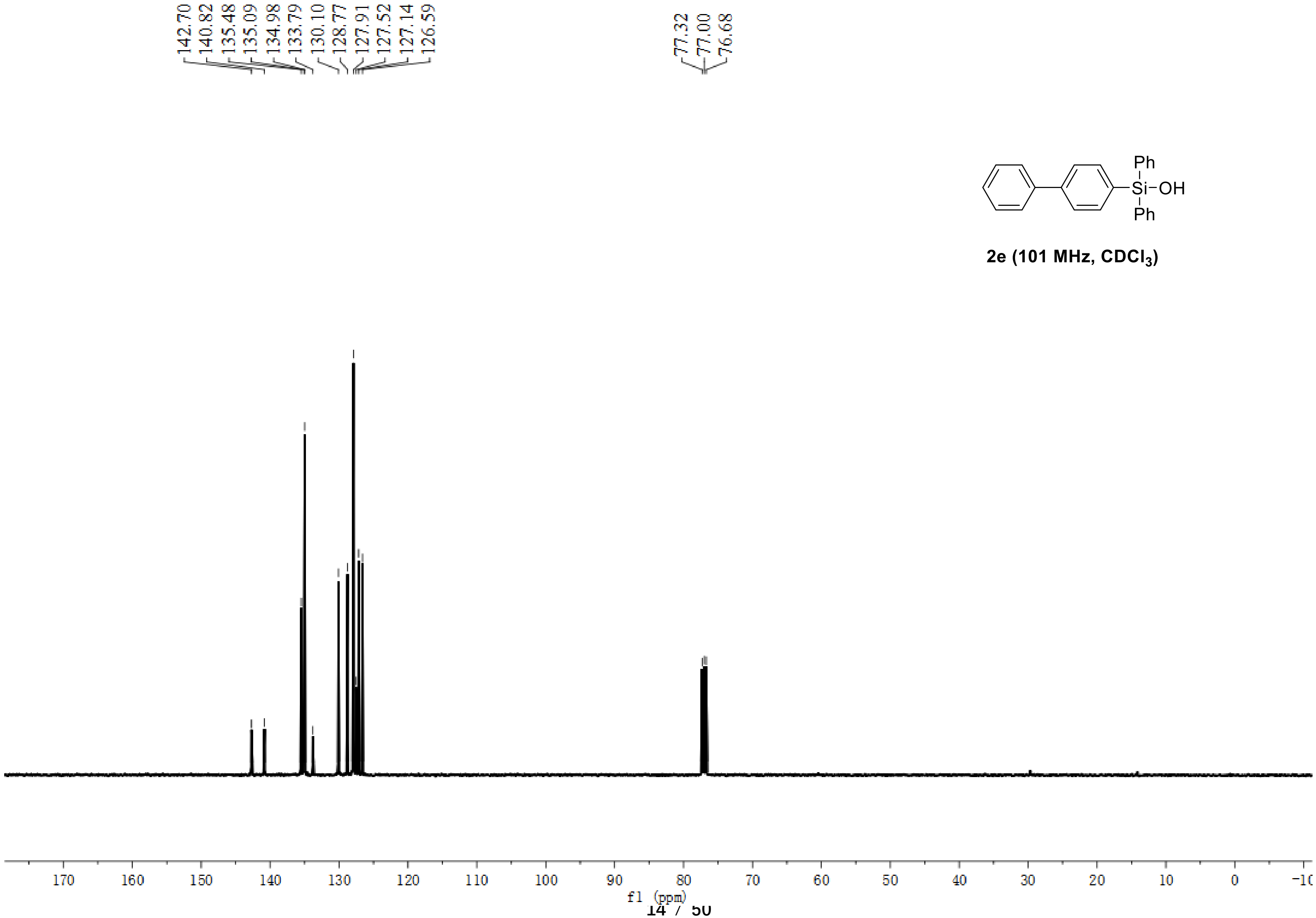
142.70
140.82
135.48
135.09
134.98
133.79
130.10
128.77
127.91
127.52
127.14
126.59
77.32
77.00
76.68
Ph
Si–OH
Ph
2e (101 MHz, CDCl3)
170
160
150
140
130
120
110
100
90
80
70
60
50
40
30
20
10
0
f1 (ppm)

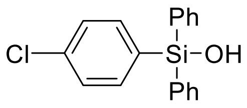

2f (400 MHz, $CDCl_3$)

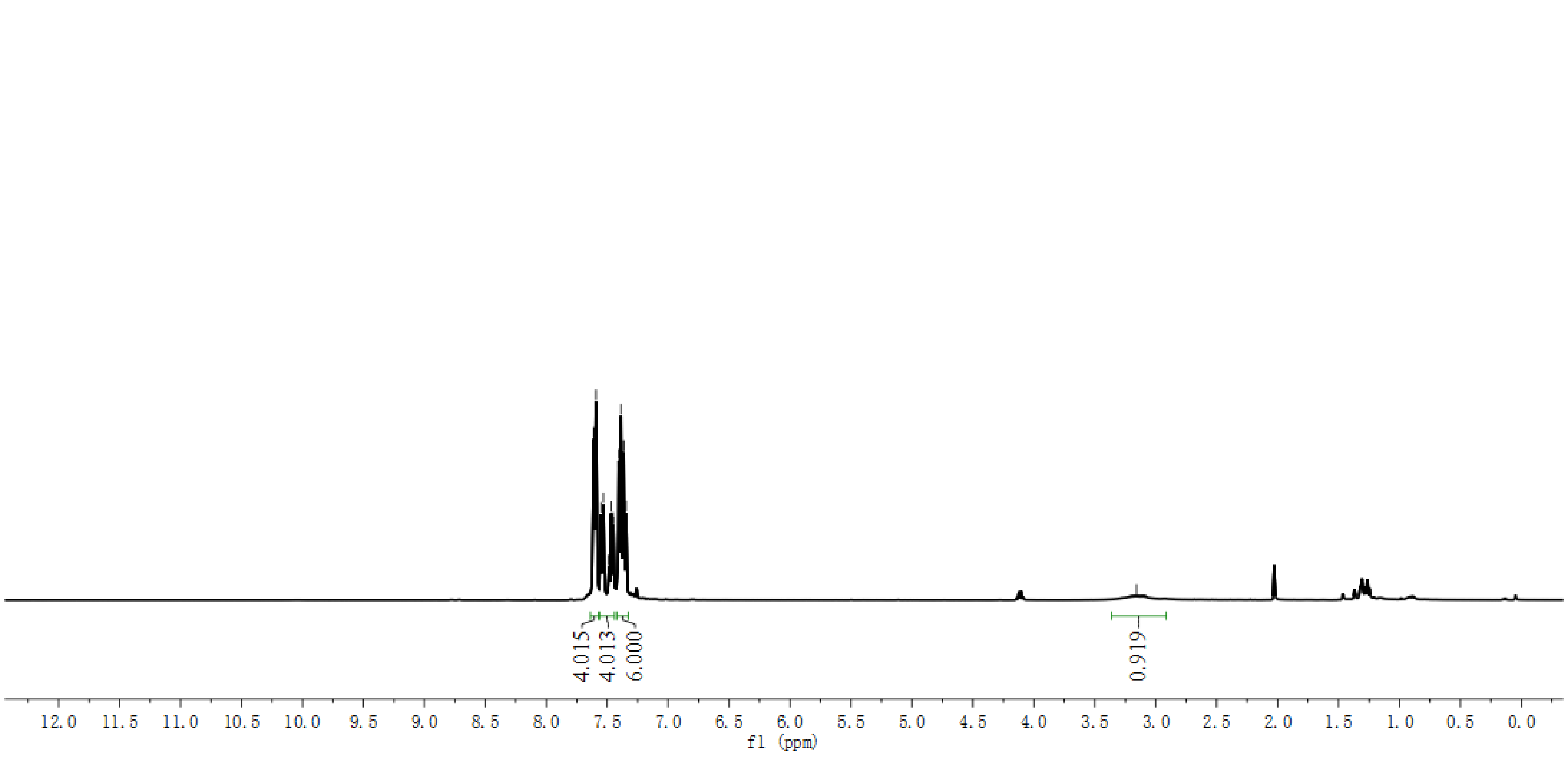

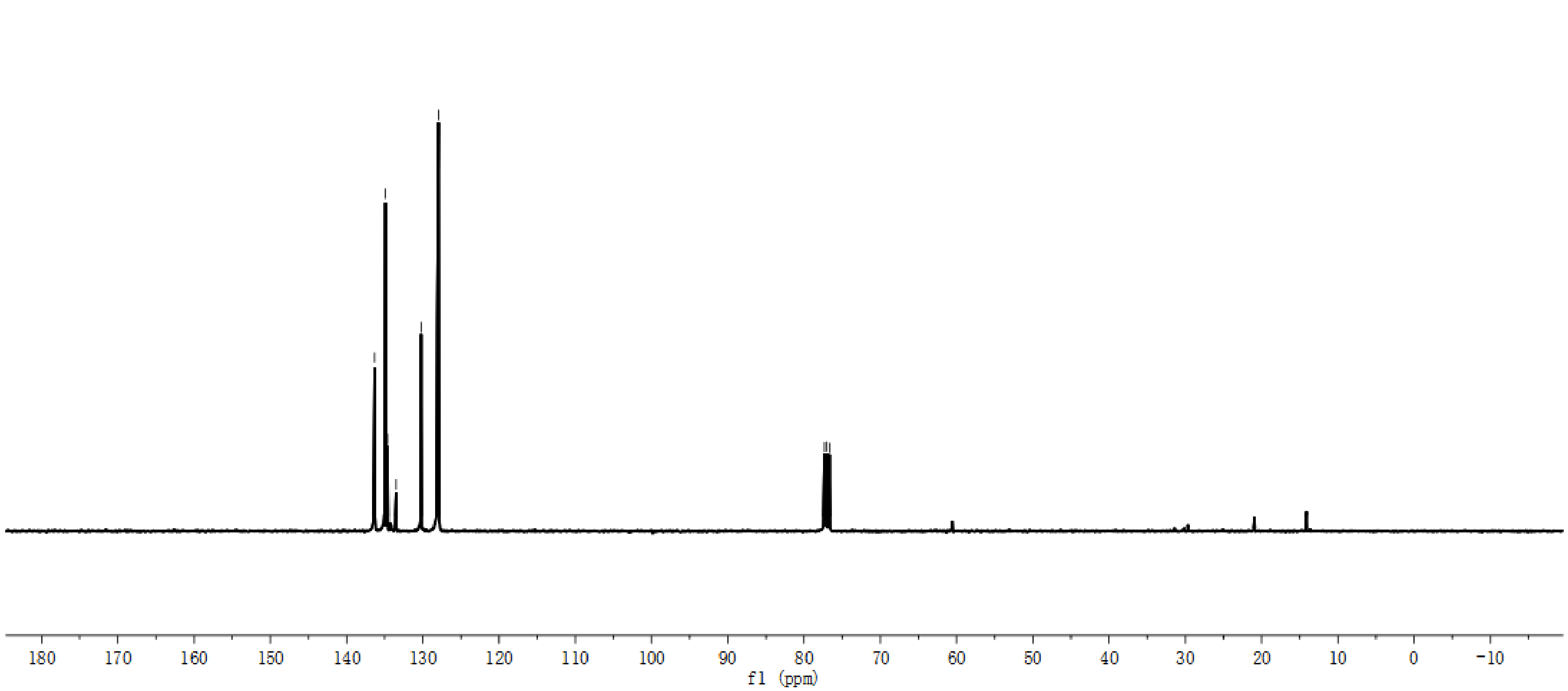

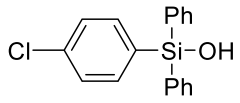

2f (101 MHz, $CDCl_3$)

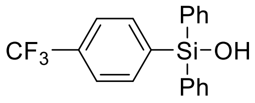

2g (400 MHz, $CDCl_3$)

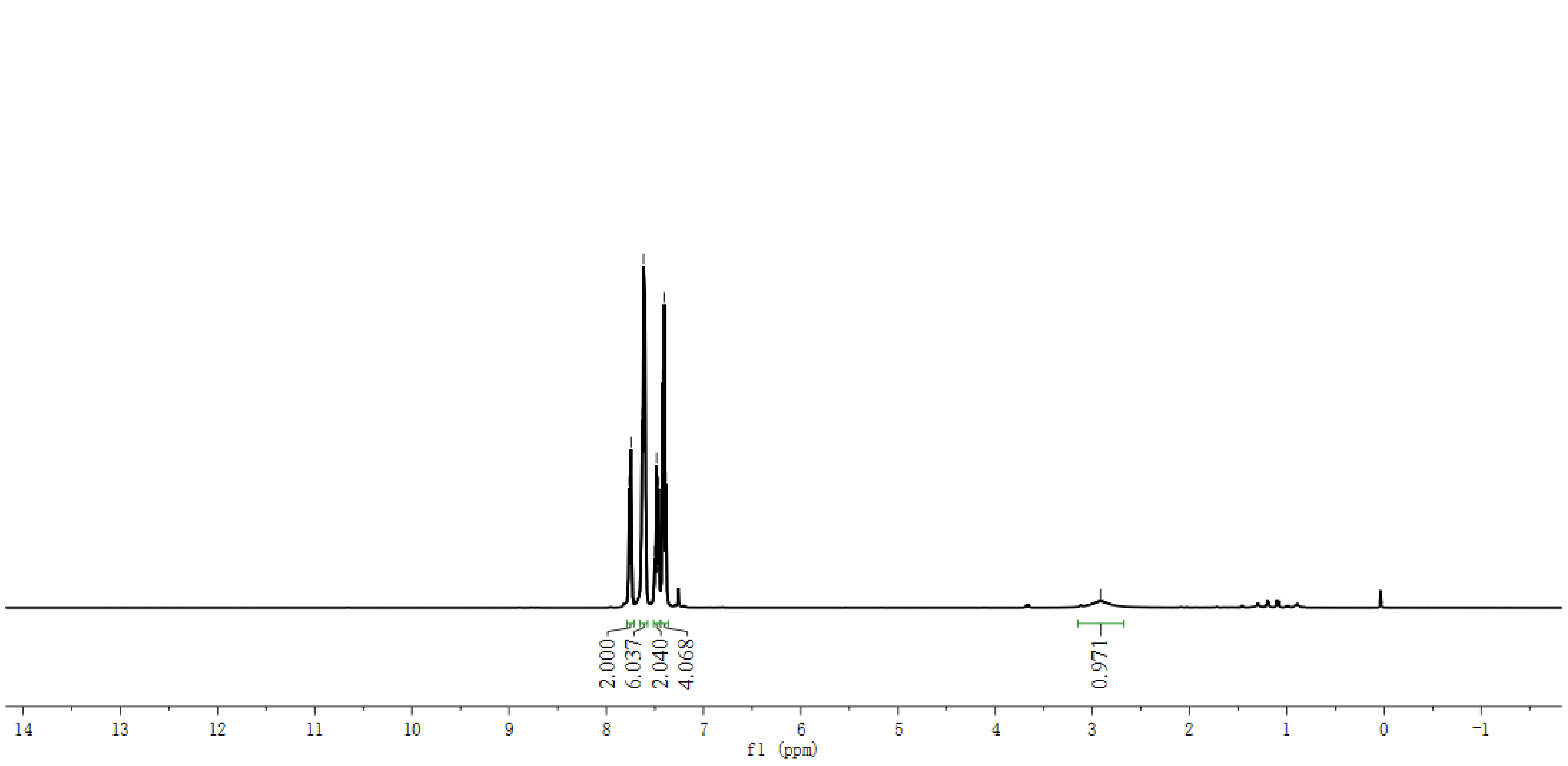

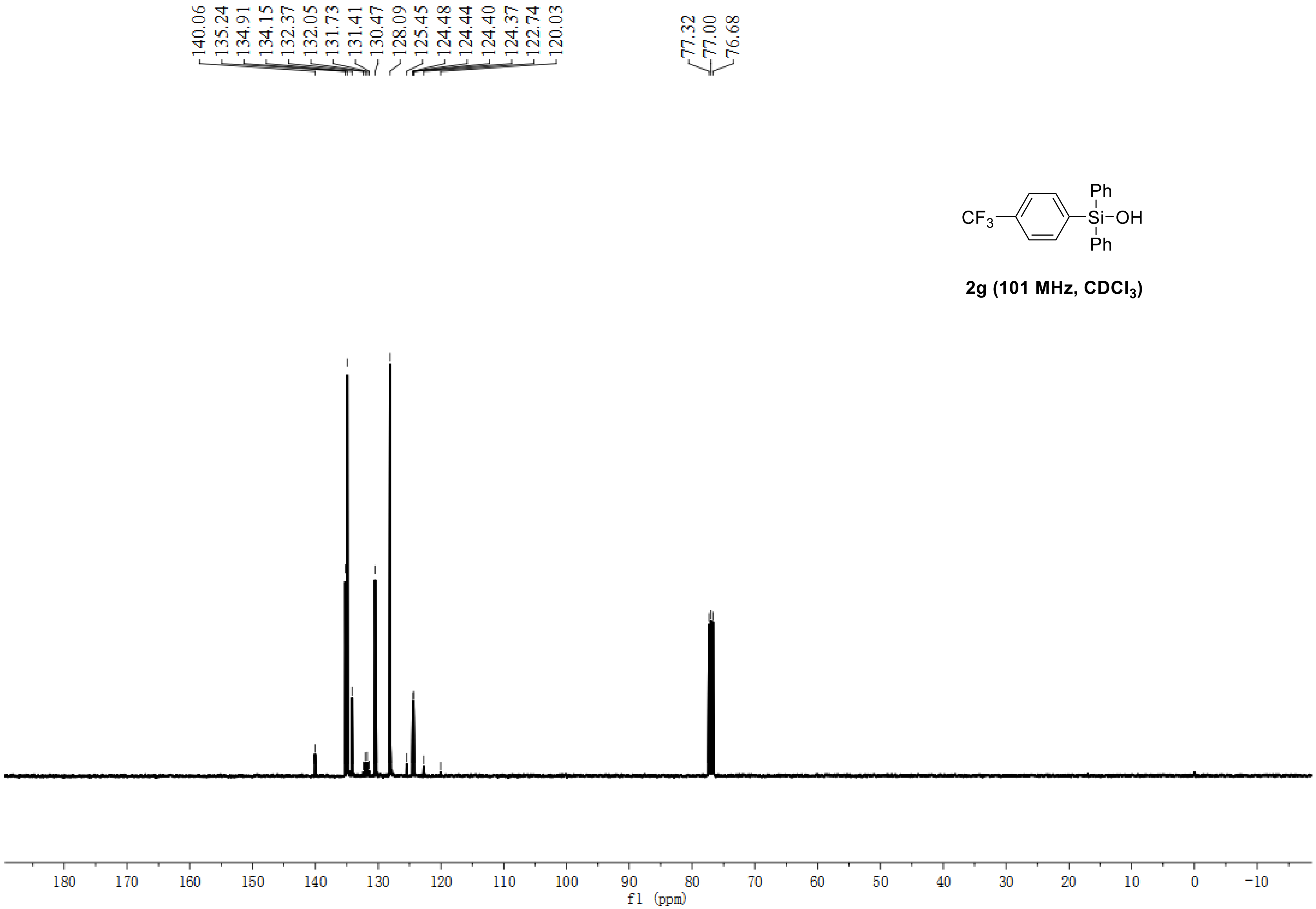

2g (101 MHz, $CDCl_3$)

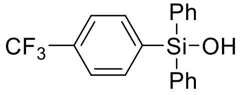

2g (376 MHz, $CDCl_3$)

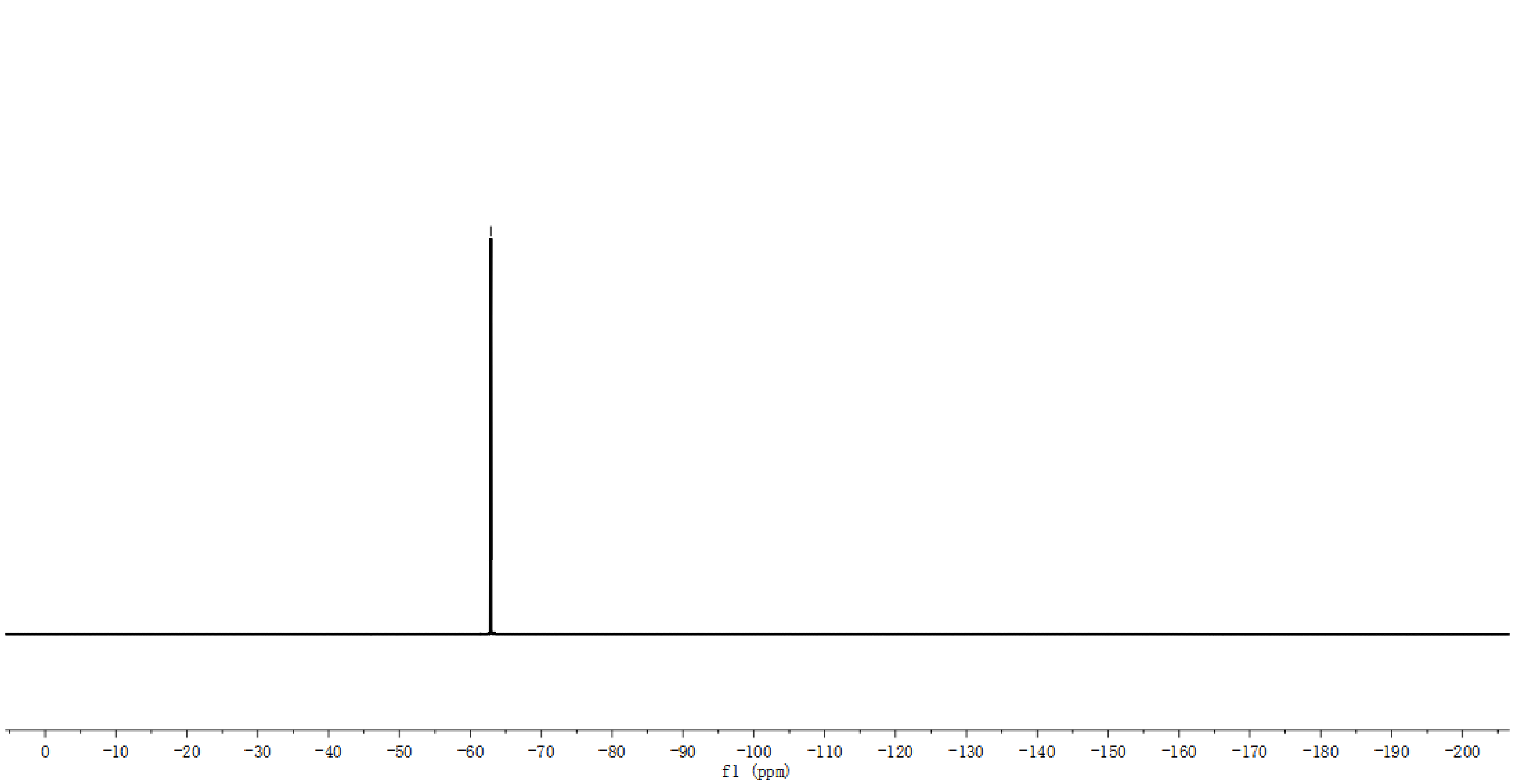

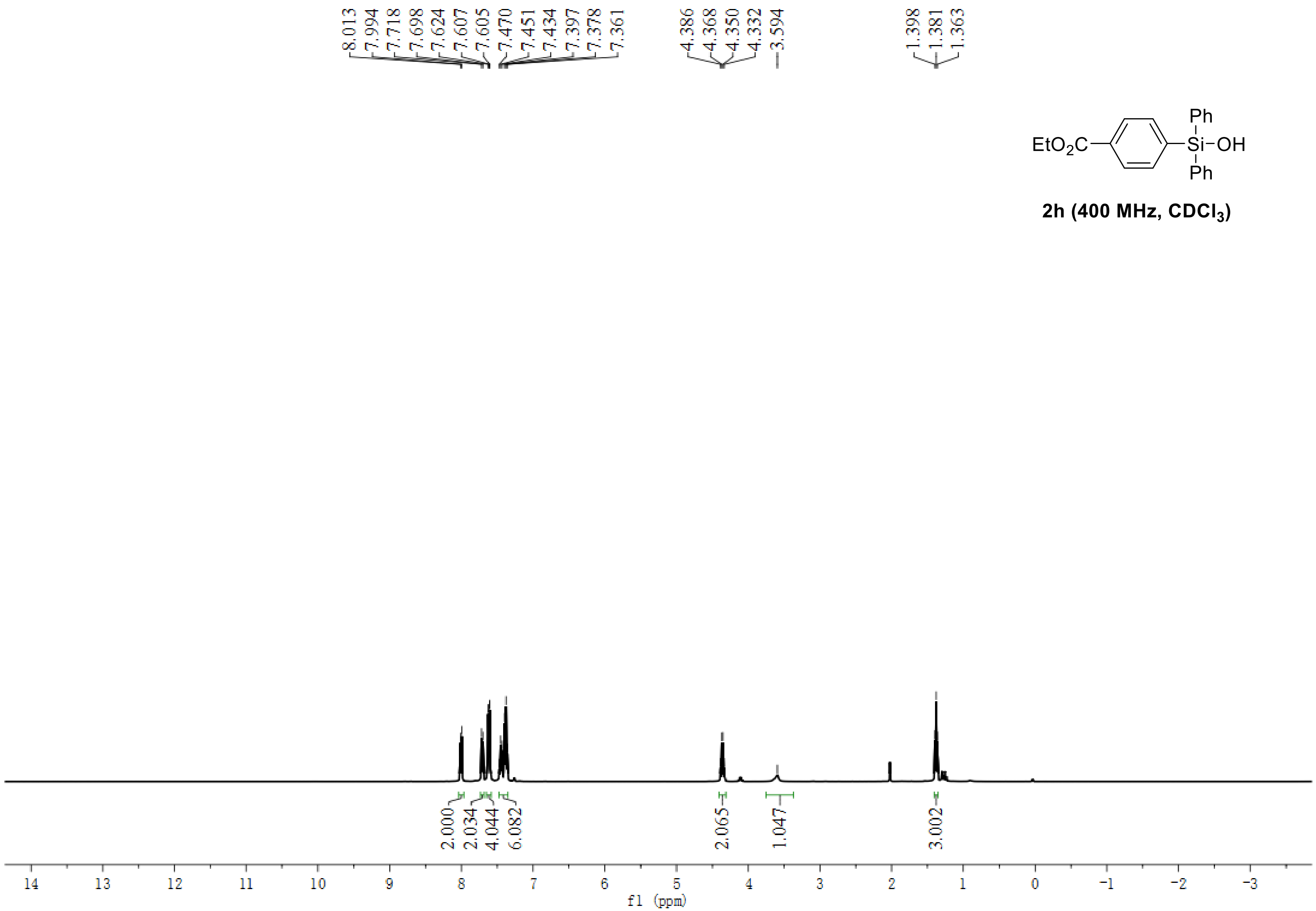

2h (400 MHz, $CDCl_3$)

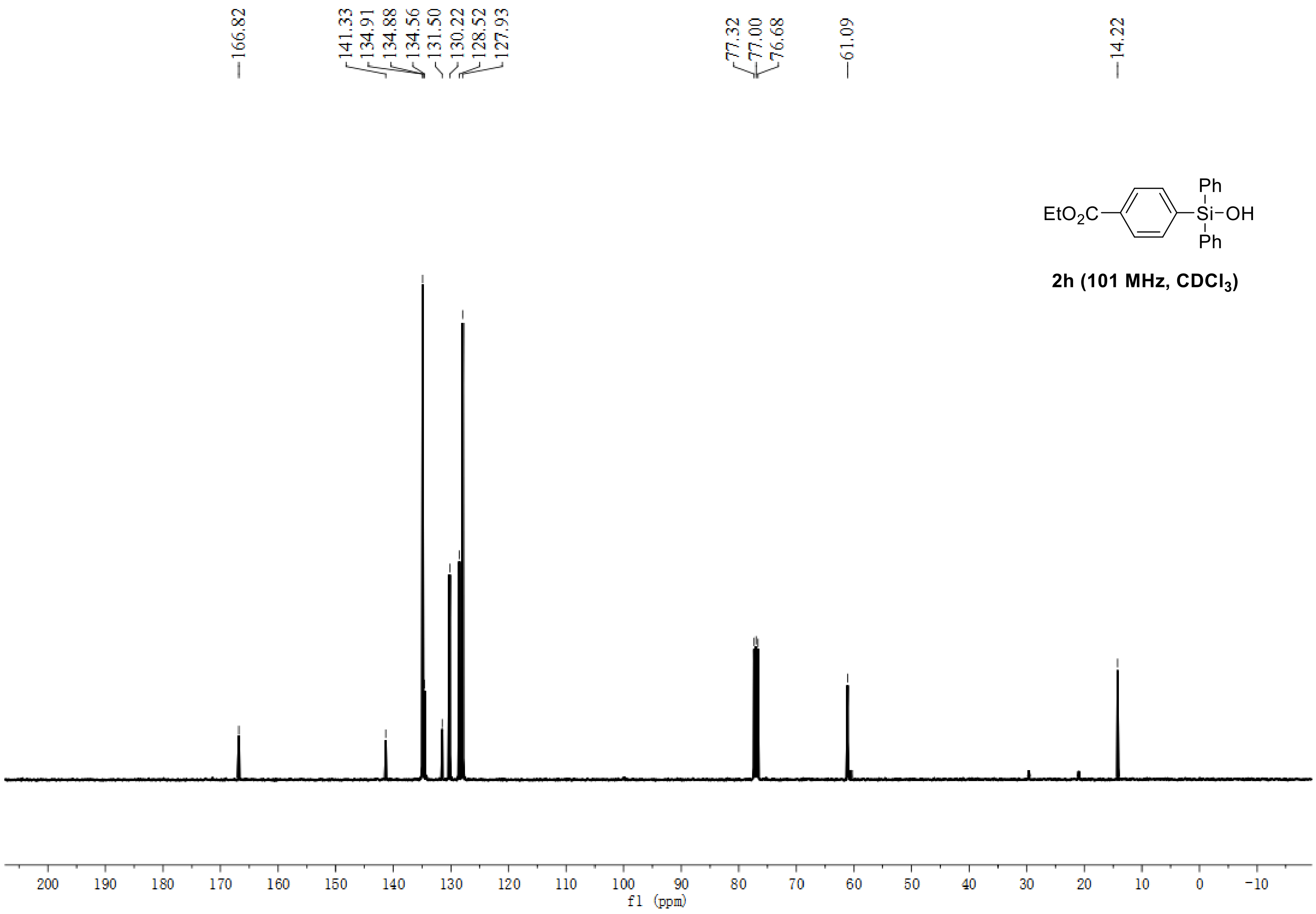

2h (101 MHz, $CDCl_3$)

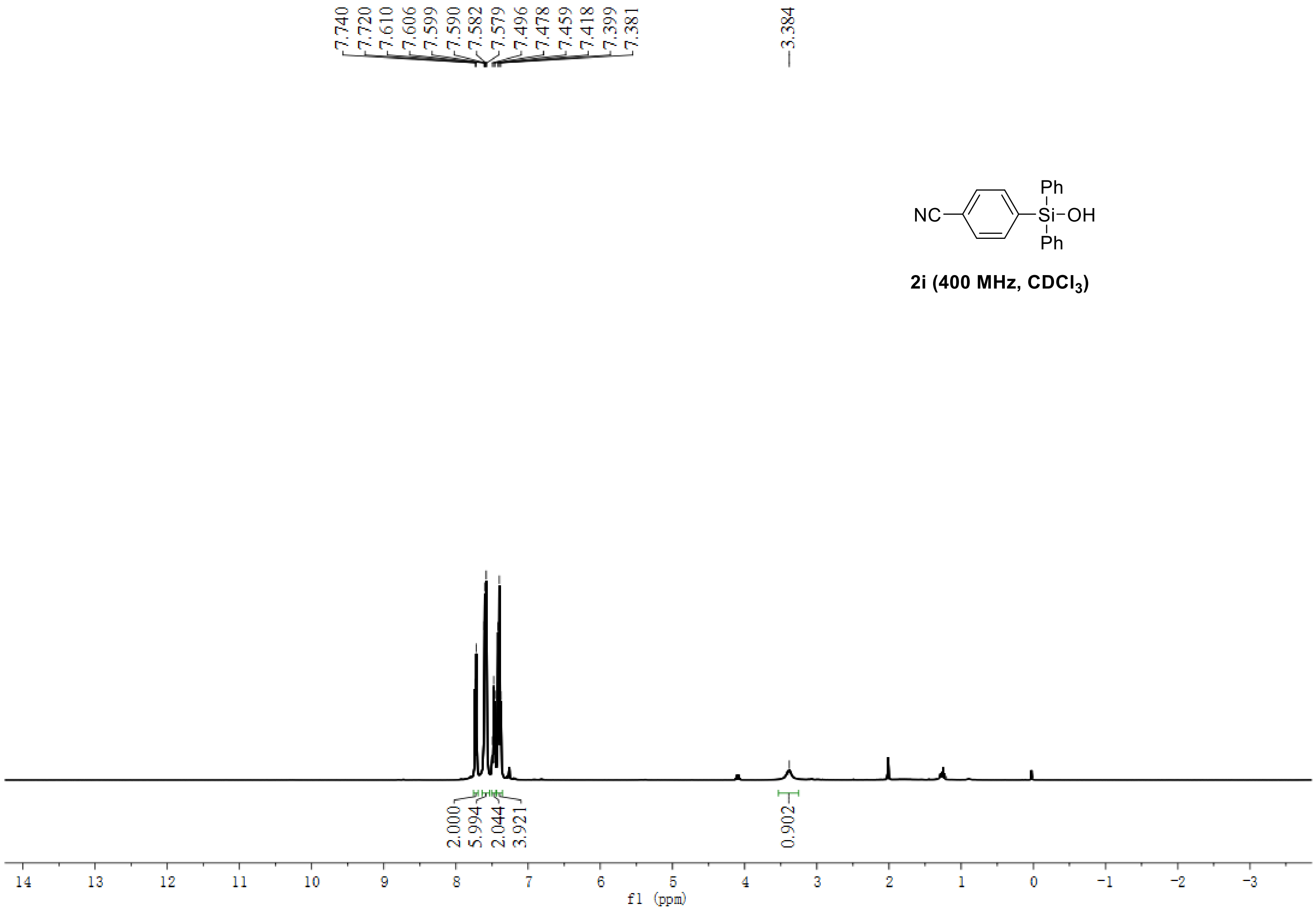

2i (400 MHz, $CDCl_3$)

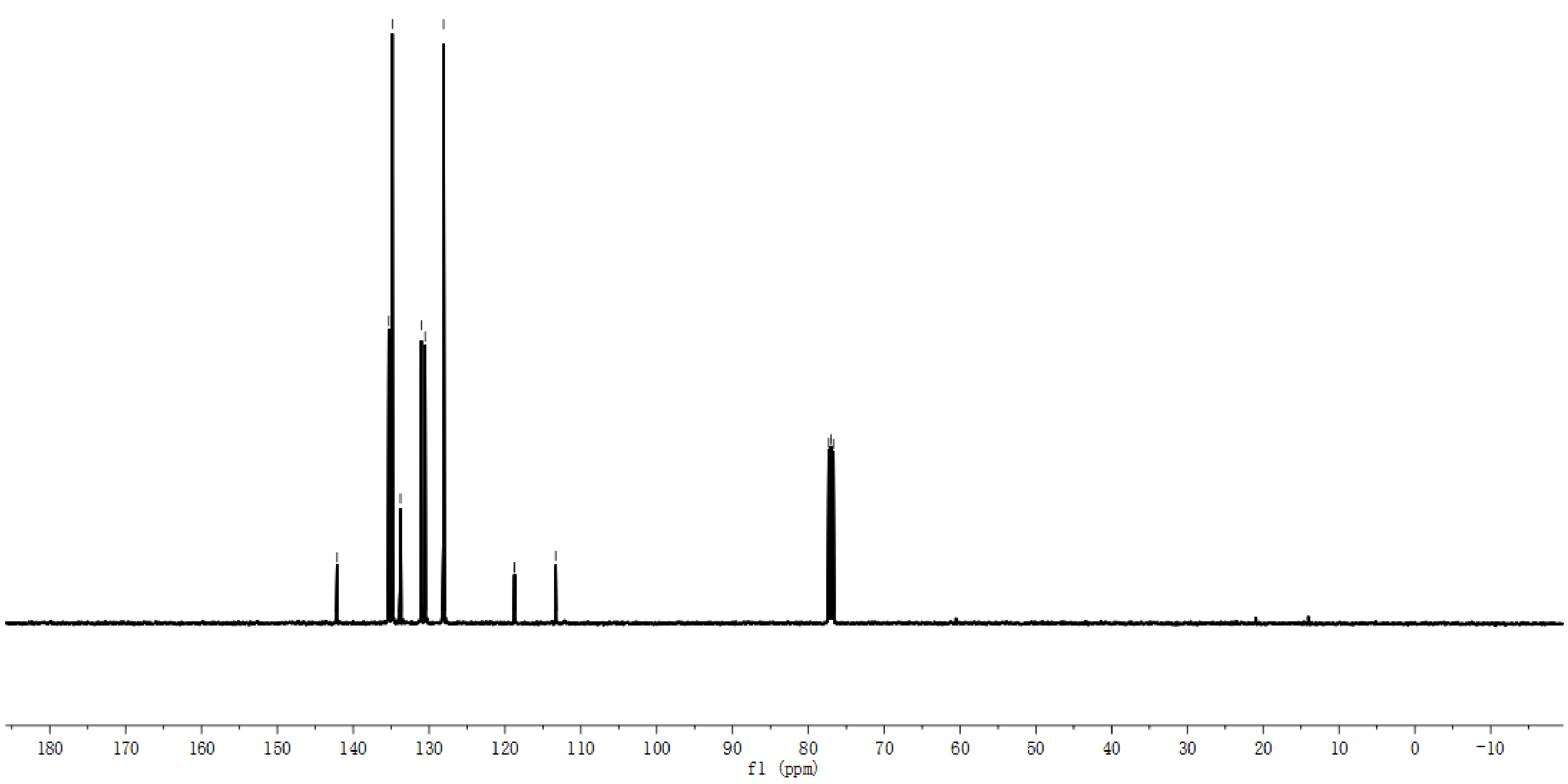

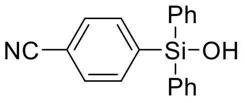

2i (101 MHz, $CDCl_3$)

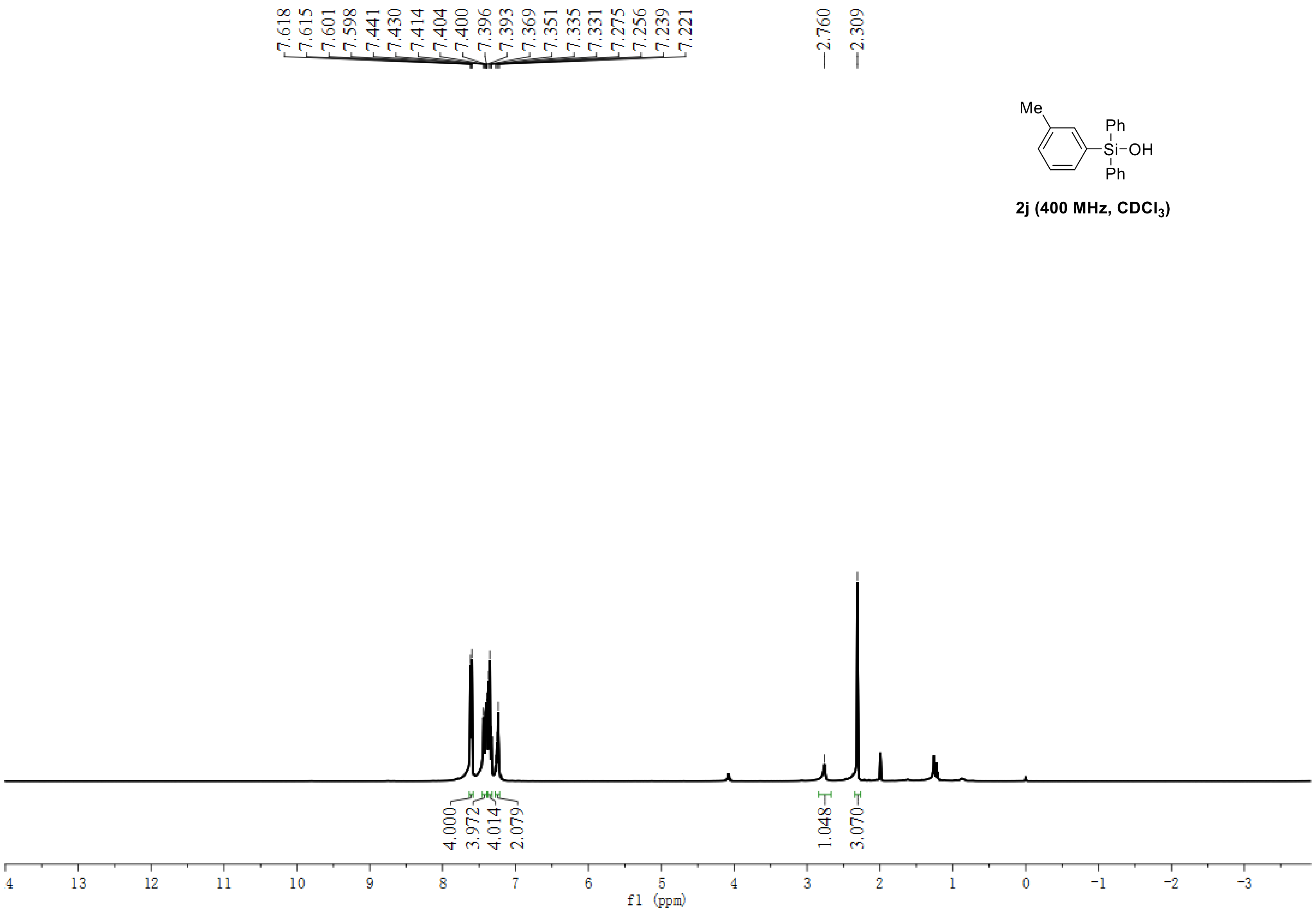

2j (400 MHz, $CDCl_3$)

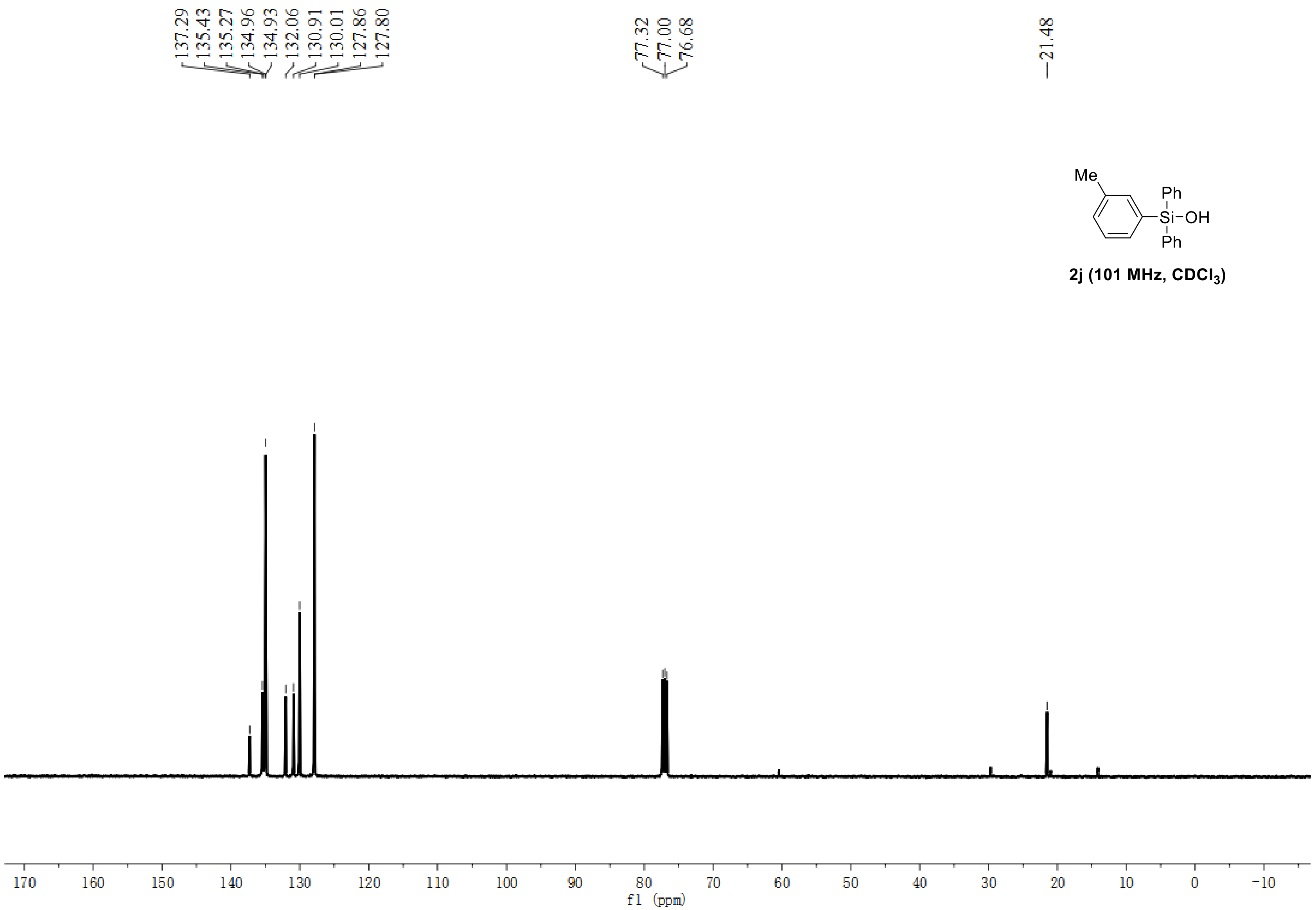
137.29
135.43
135.27
134.96
134.93
132.06
130.91
130.01
127.86
127.80
77.32
77.00
76.68
21.48
Me
Ph
Si
OH
Ph
2j (101 MHz, CDCl3)
170
160
150
140
130
120
110
100
90
80
70
60
50
40
30
20
10
0
-10
f1 (ppm)

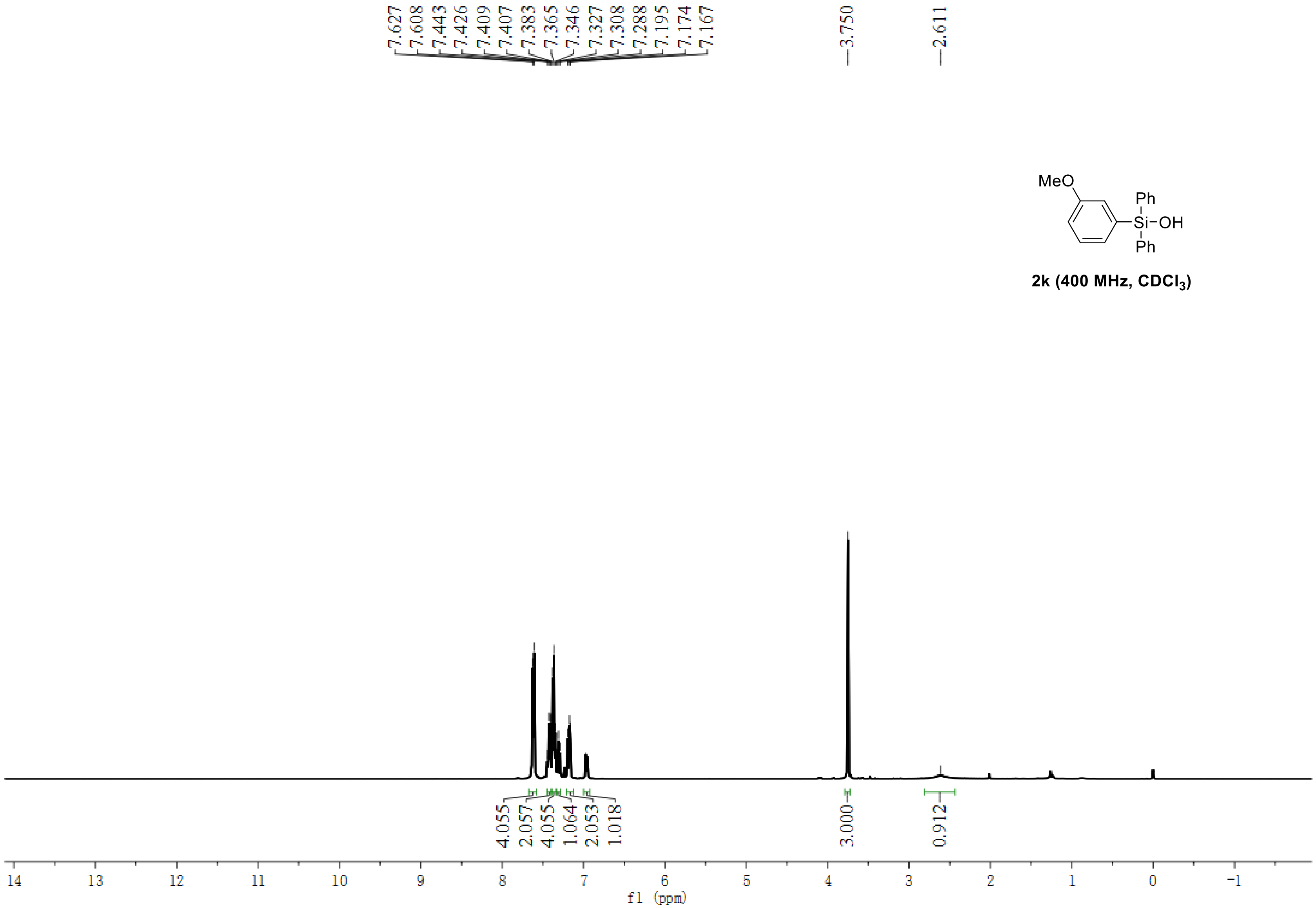

2k (400 MHz, $CDCl_3$)

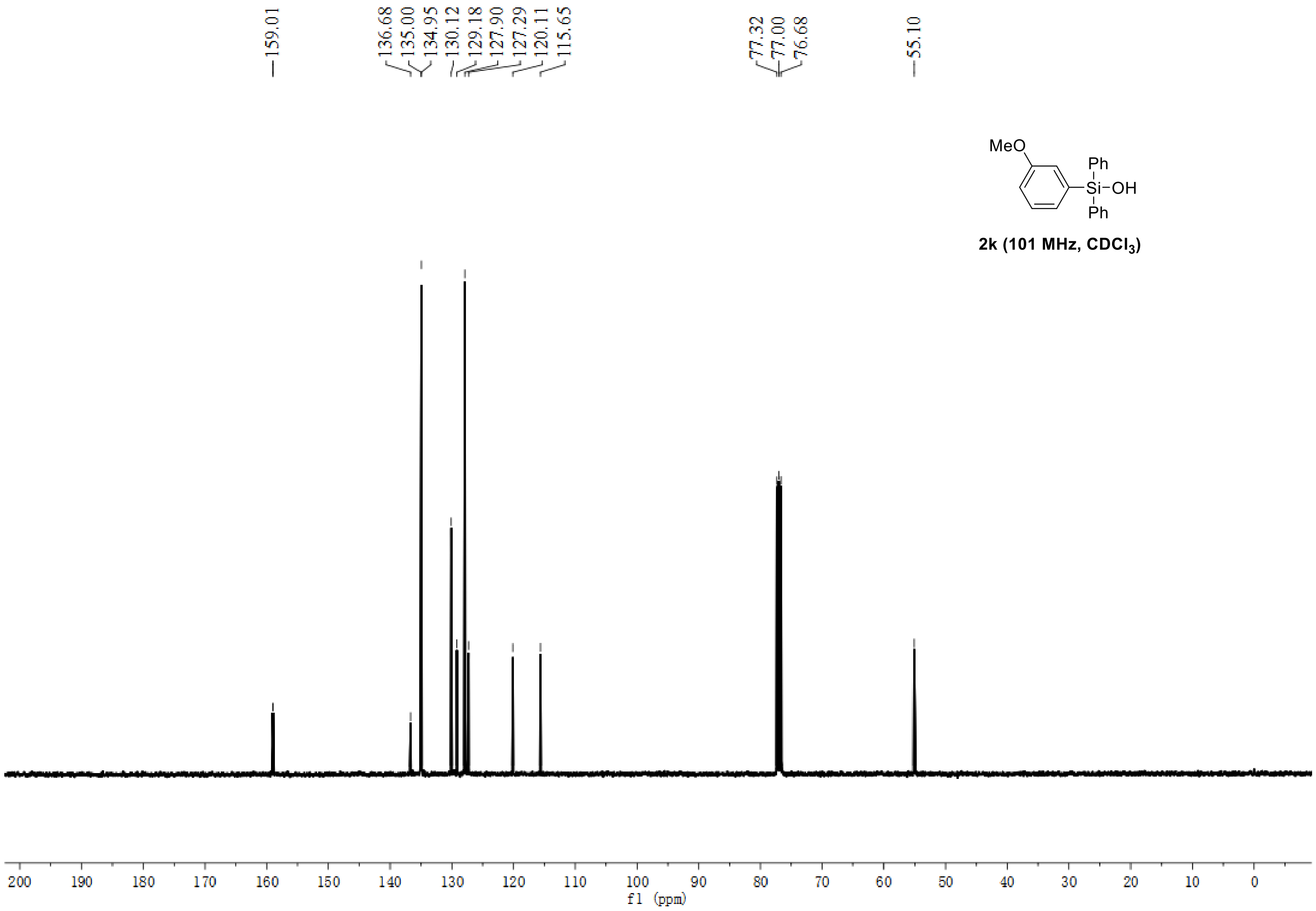
159.01
136.68
135.00
134.95
130.12
129.18
127.90
127.29
120.11
115.65
77.32
77.00
76.68
55.10
MeO
Ph
Si–OH
Ph
2k (101 MHz, CDCl3)
200
190
180
170
160
150
140
130
120
110
100
90
80
70
60
50
40
30
20
10
0
f1 (ppm)

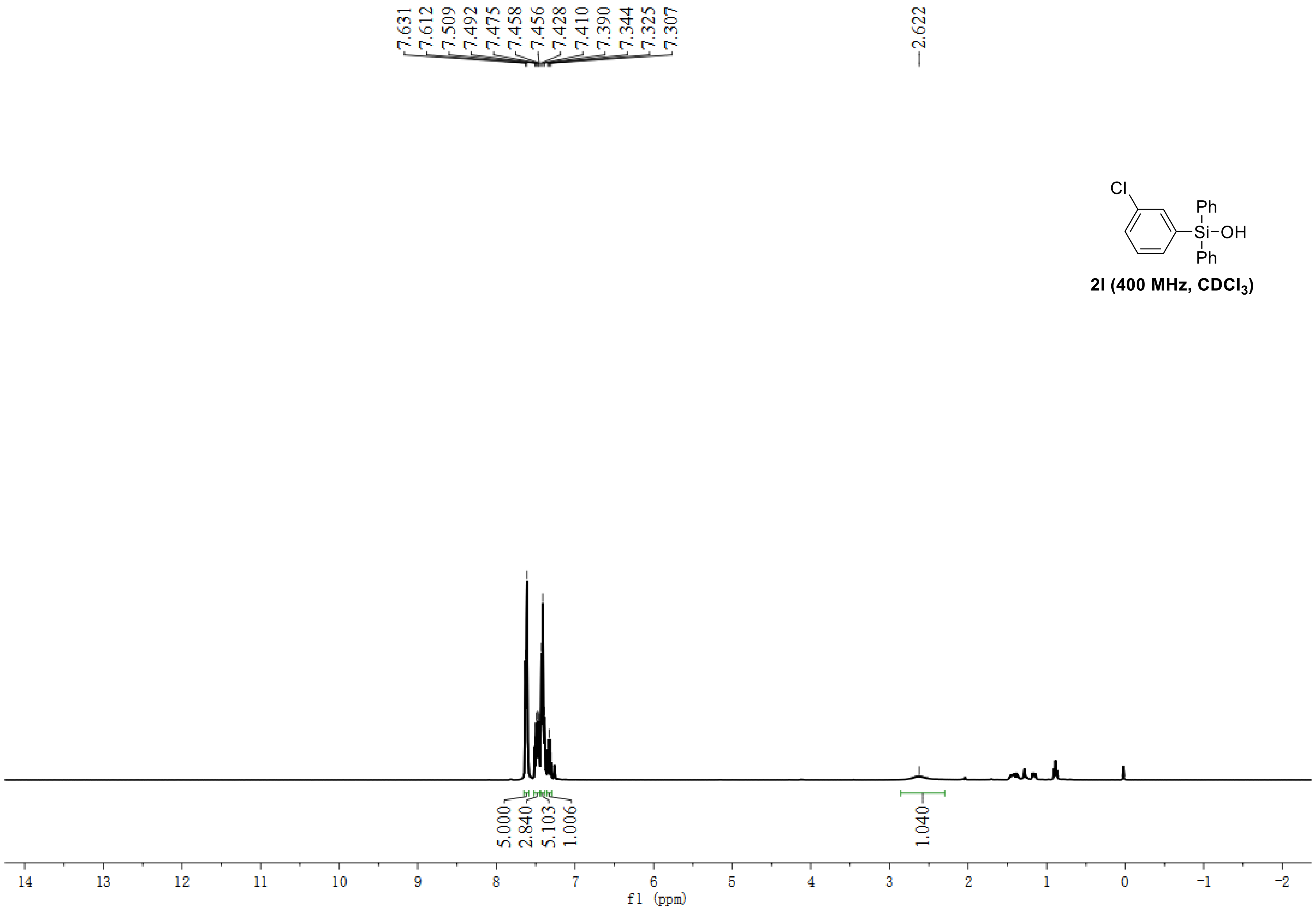

2l (400 MHz, CDCl$_3$)

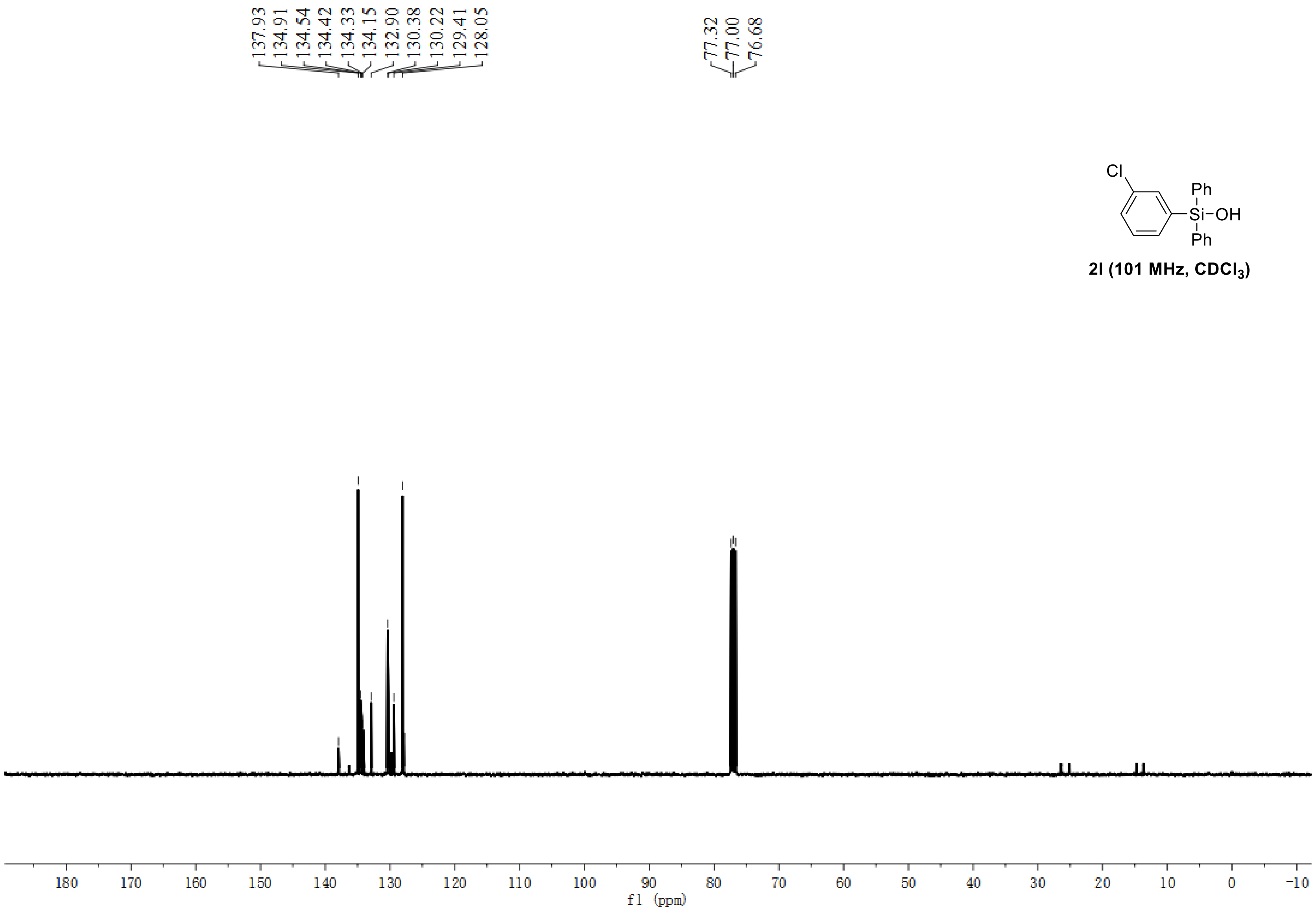
137.93
134.91
134.54
134.42
134.33
134.15
132.90
130.38
130.22
129.41
128.05
77.32
77.00
76.68
Cl
Ph
Si-OH
Ph
2l (101 MHz, CDCl3)
180 170 160 150 140 130 120 110 100 90 80 70 60 50 40 30 20 10 0 -10
f1 (ppm)

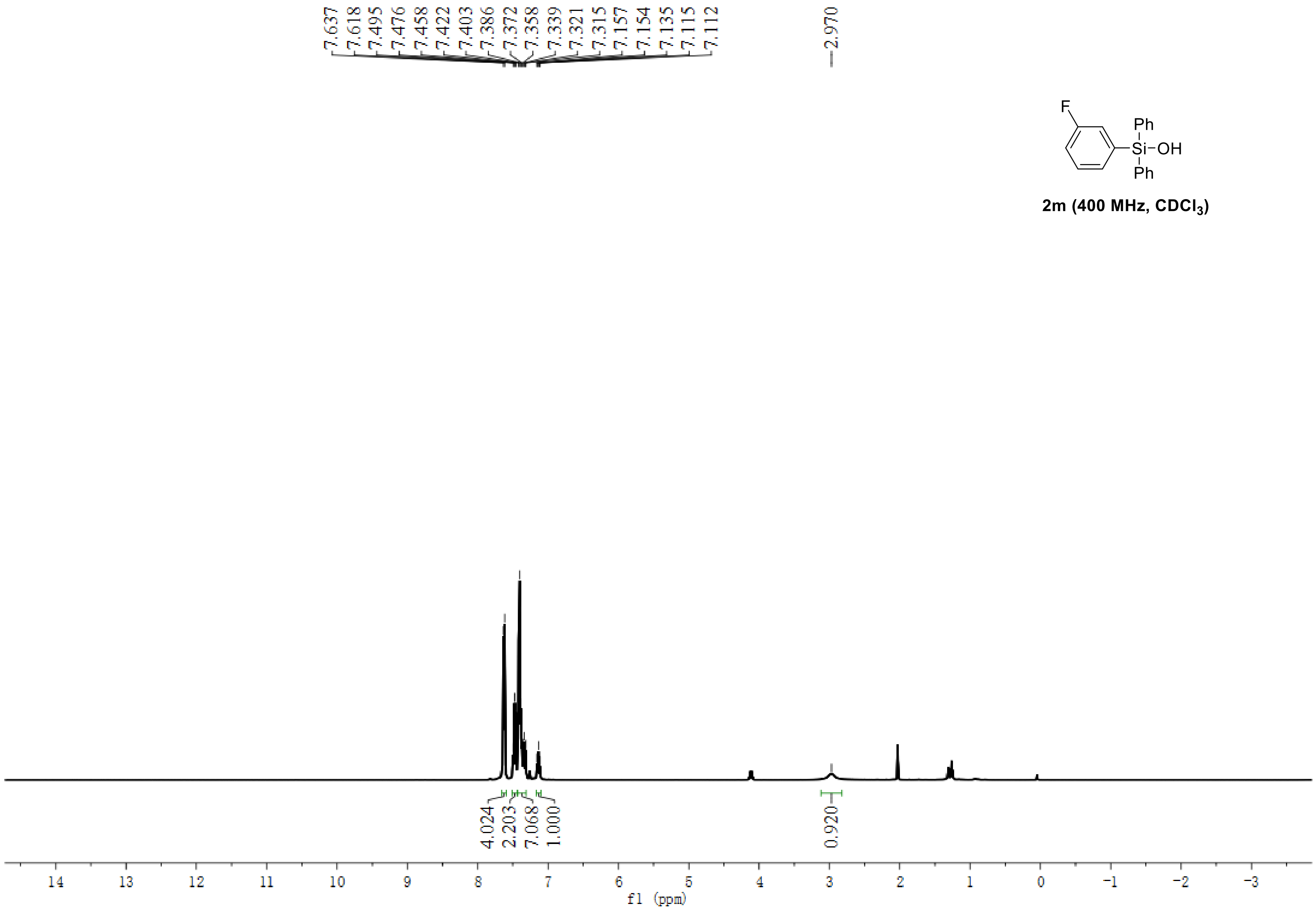

2m (400 MHz, $CDCl_3$)

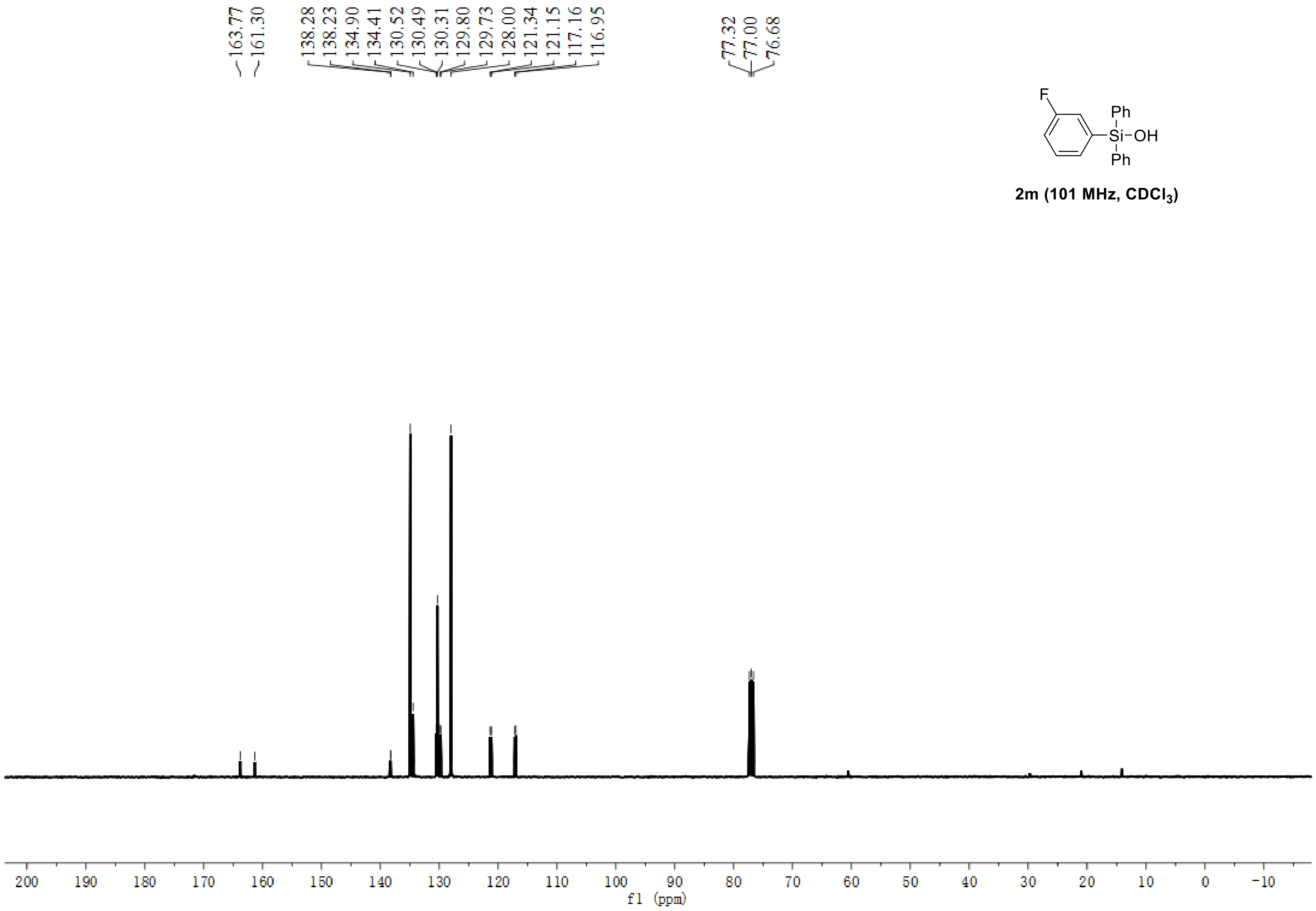

2m (101 MHz, $CDCl_3$)

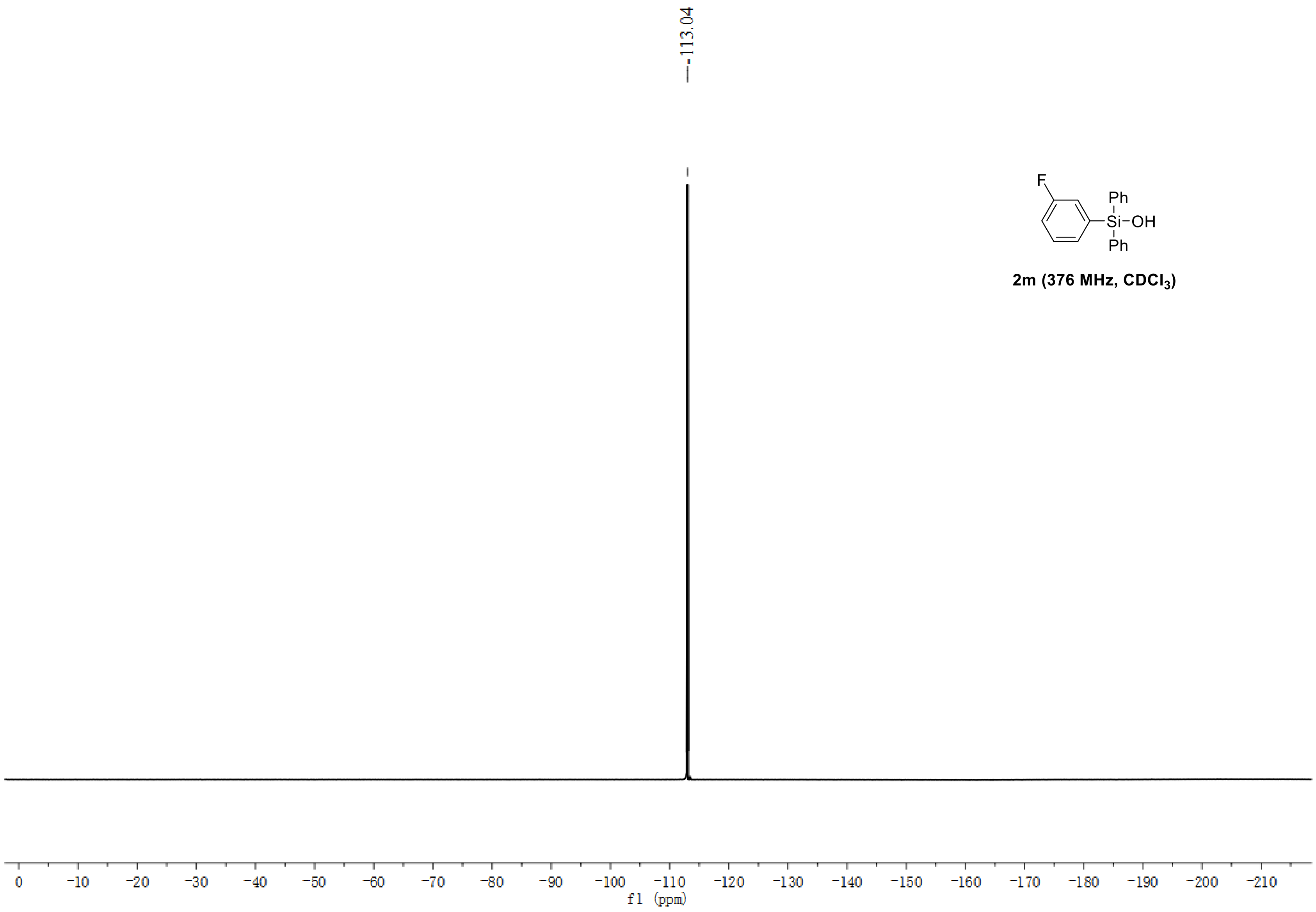

2m (376 MHz, $CDCl_3$)

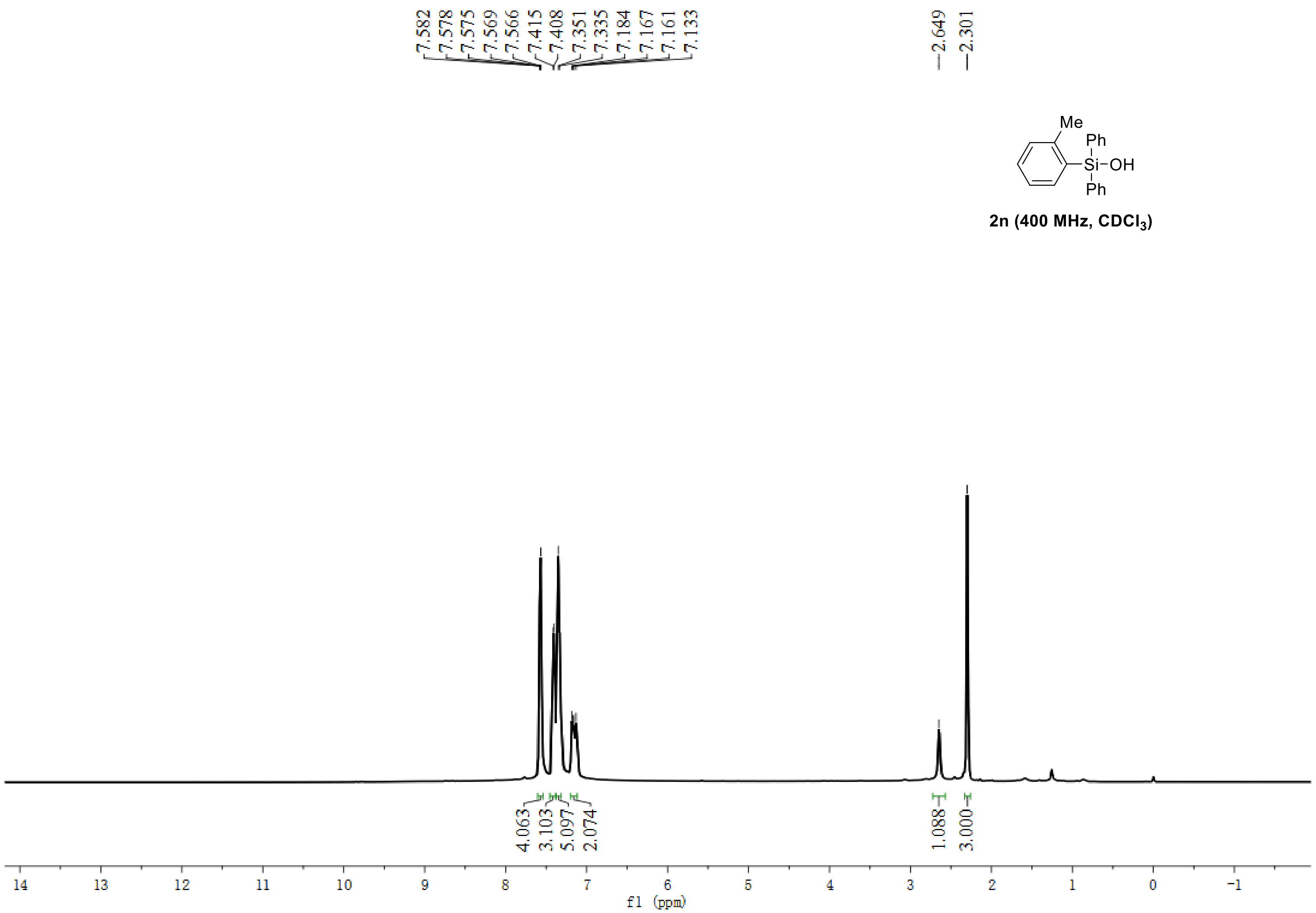

2n (400 MHz, $CDCl_3$)

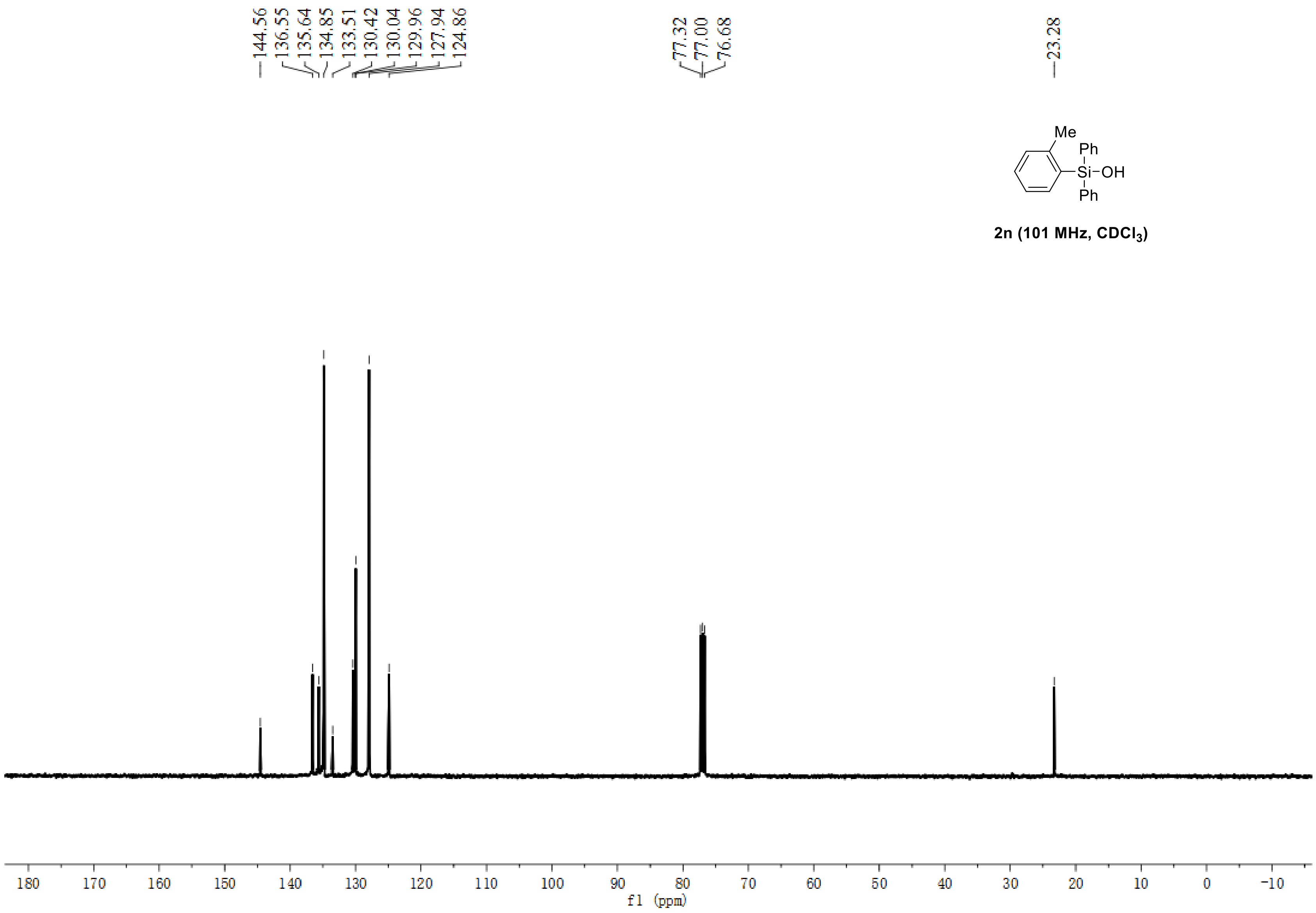

144.56
136.55
135.64
134.85
133.51
130.42
130.04
129.96
127.94
124.86
77.32
77.00
76.68
23.28
Me
Ph
Si
OH
Ph
2n (101 MHz, CDCl3)
180
170
160
150
140
130
120
110
100
90
80
70
60
50
40
30
20
10
0
-10
f1 (ppm)

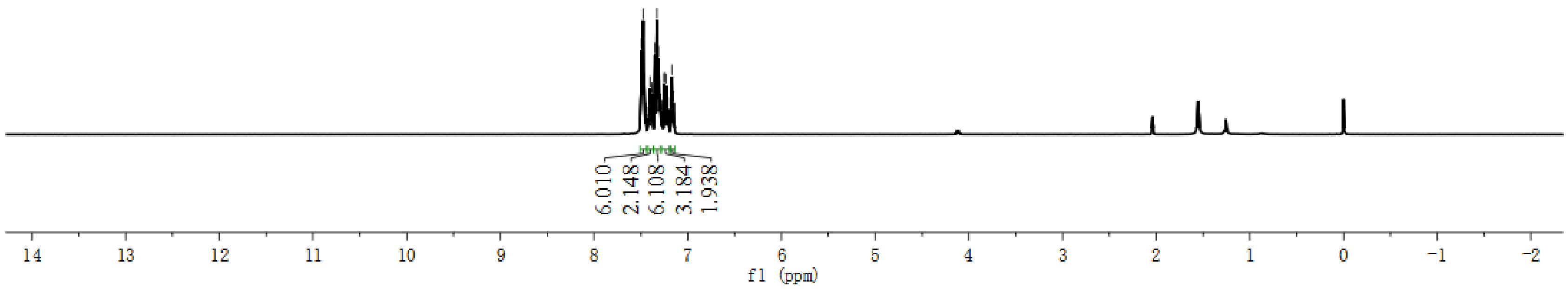

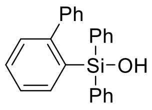

2o (400 MHz, $CDCl_3$)

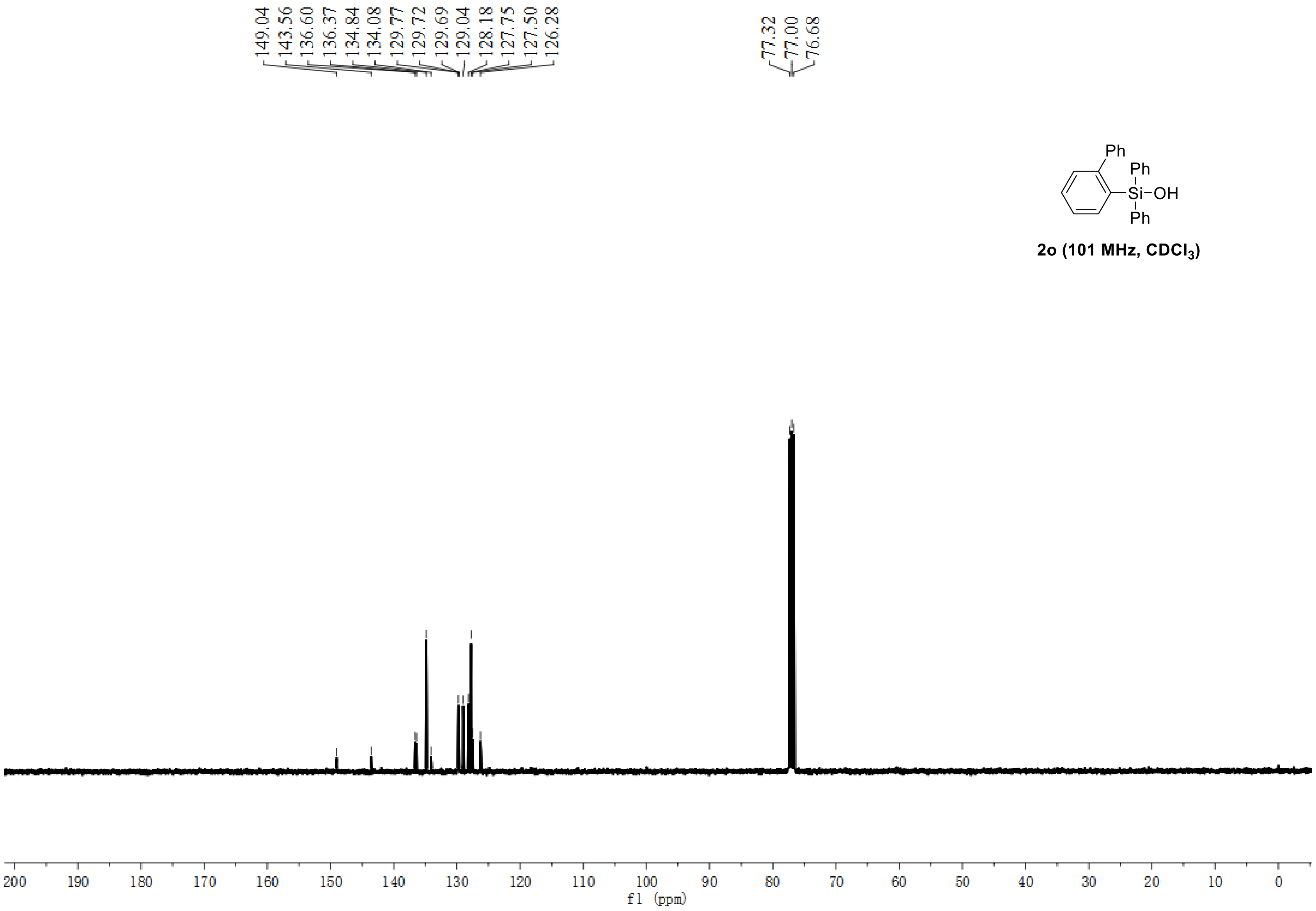

2o (101 MHz, $CDCl_3$)

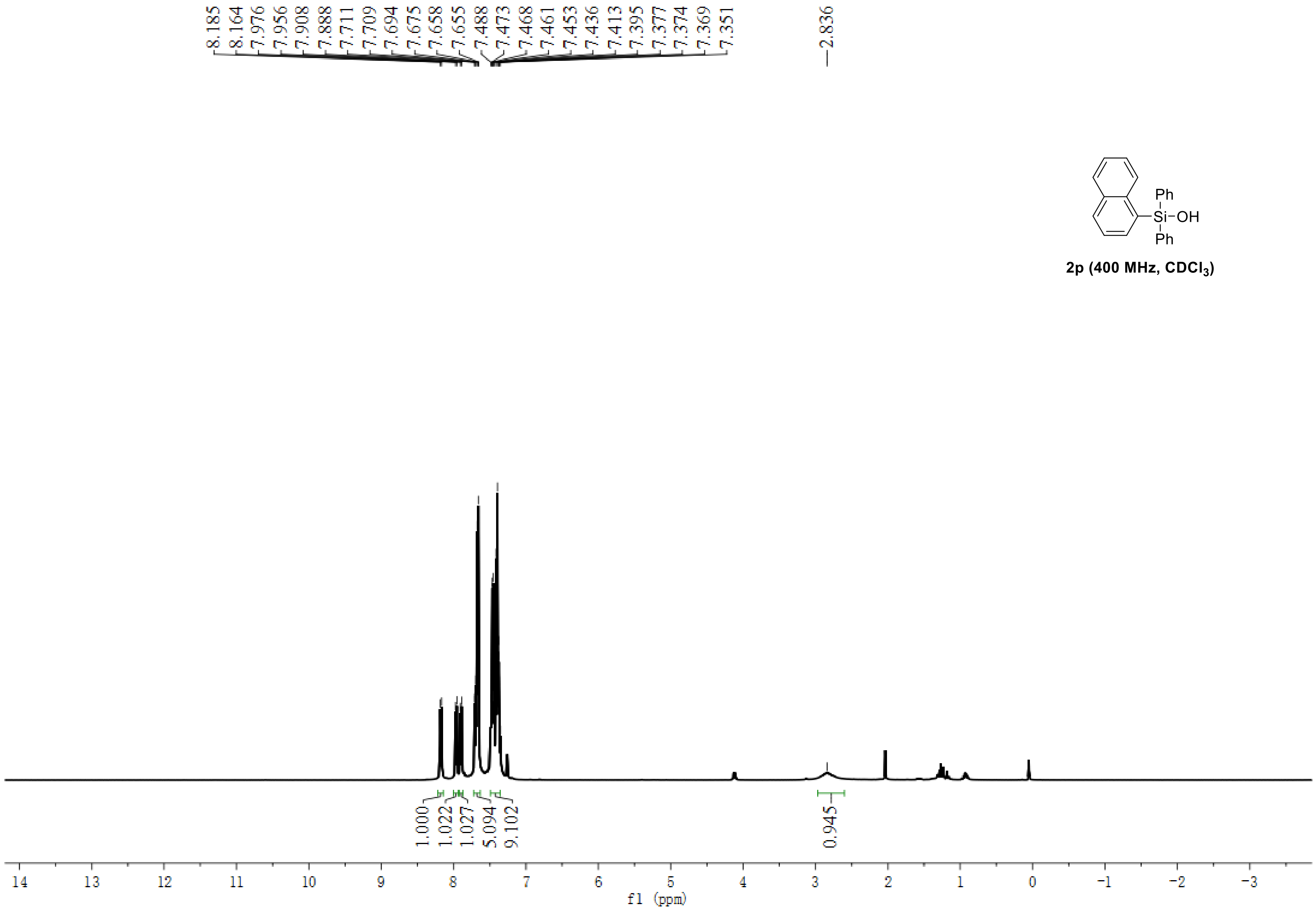

2p (400 MHz, $CDCl_3$)

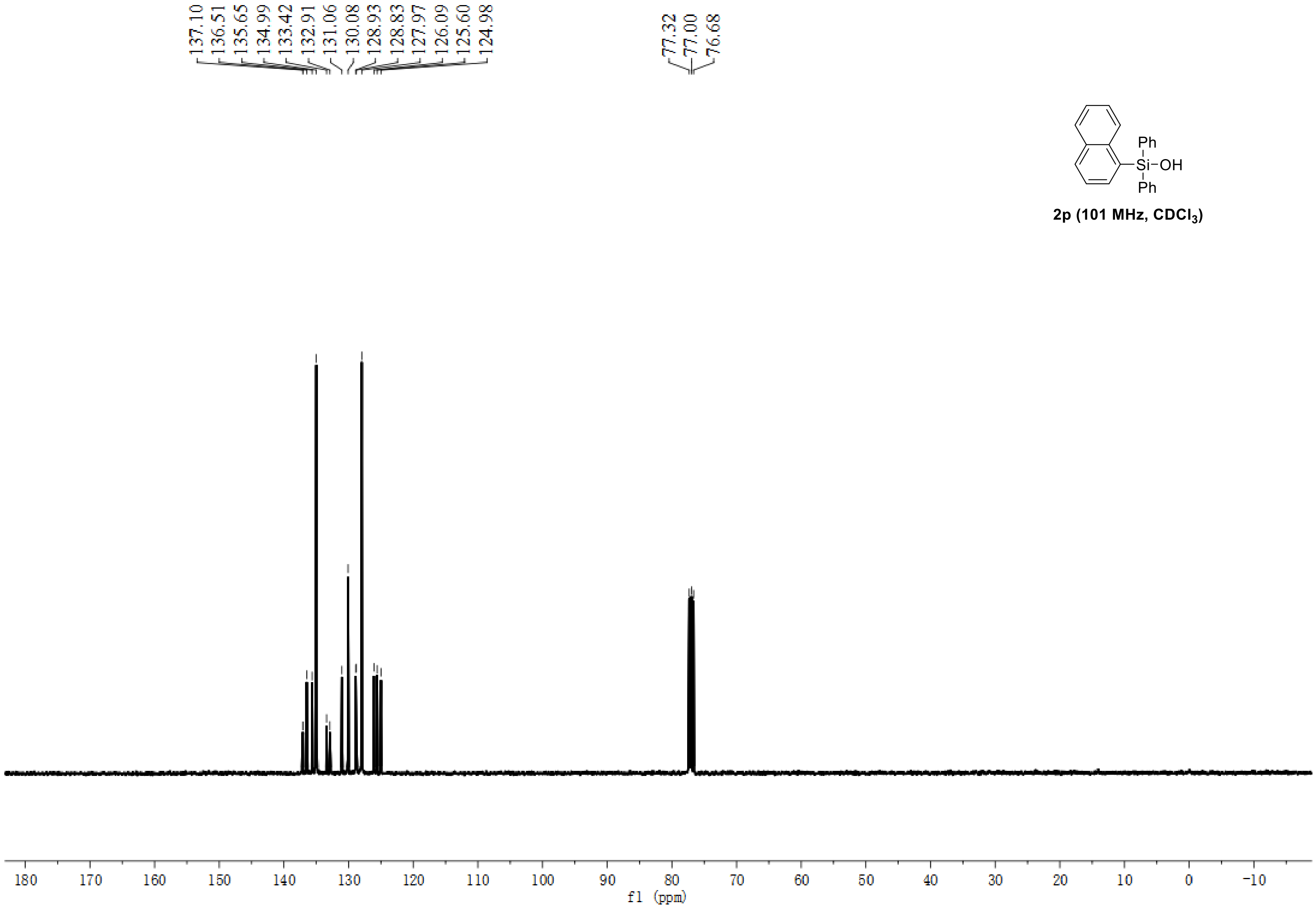

2p (101 MHz, $CDCl_3$)

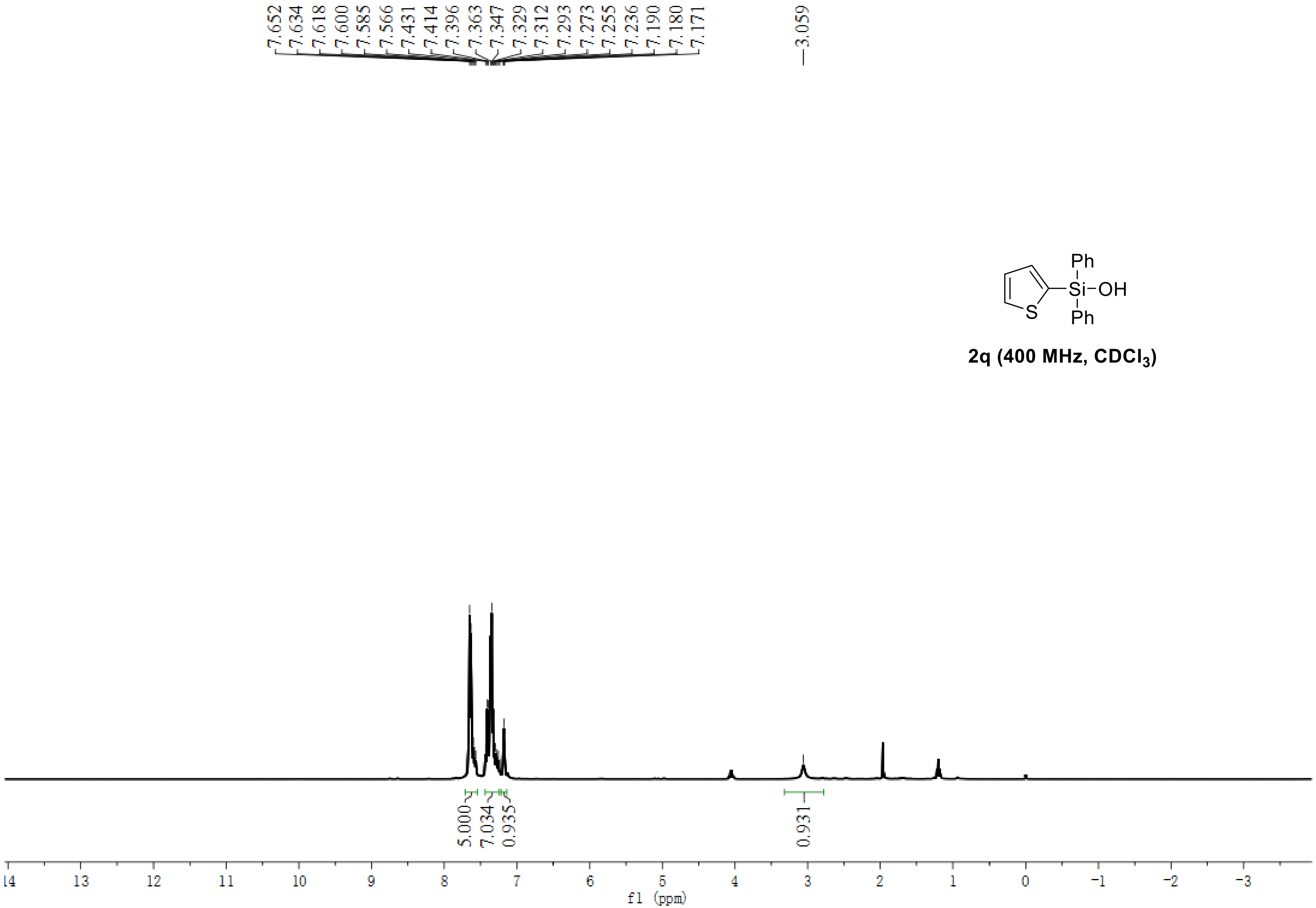

2q (400 MHz, $CDCl_3$)

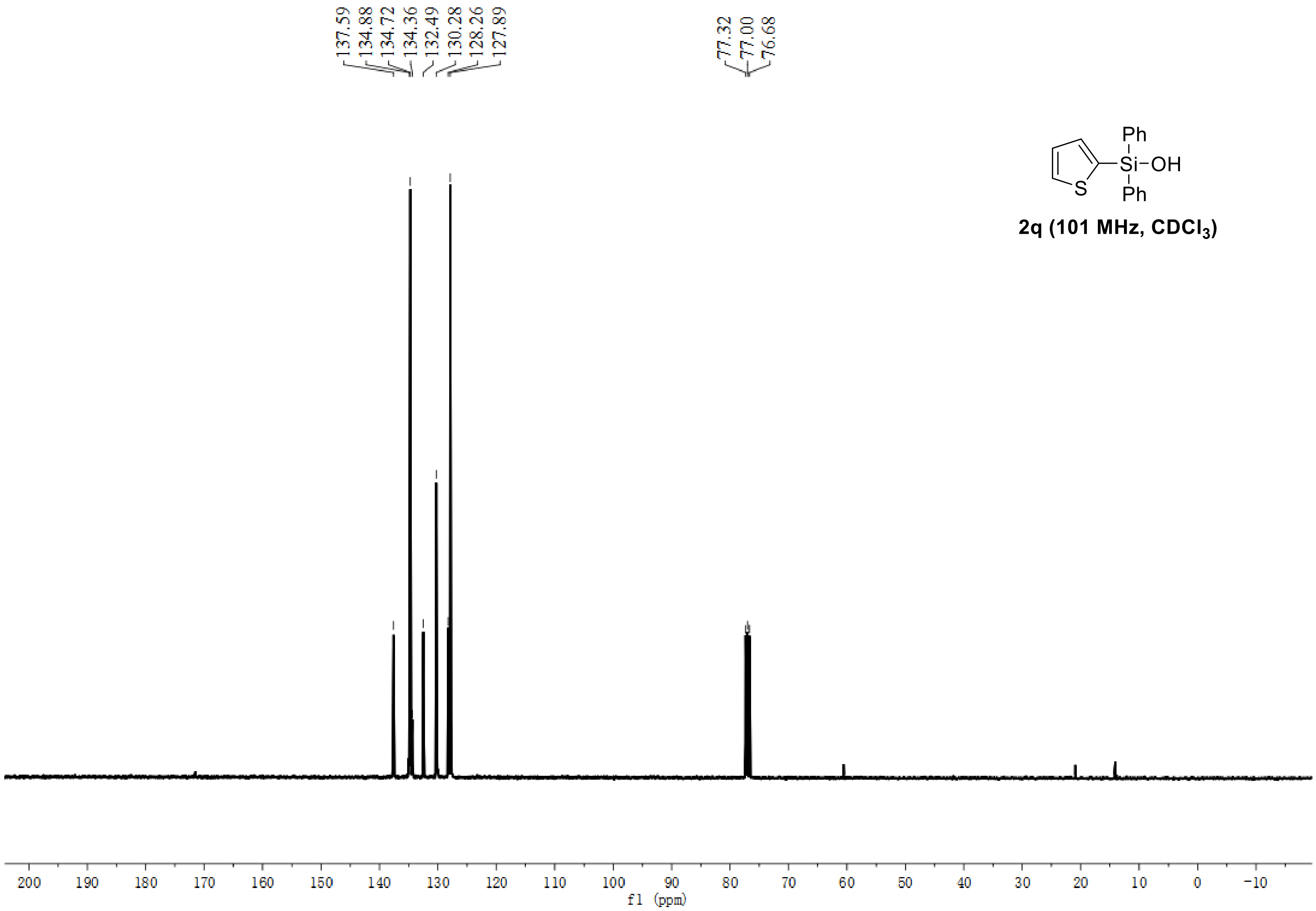

2q (101 MHz, $CDCl_3$)

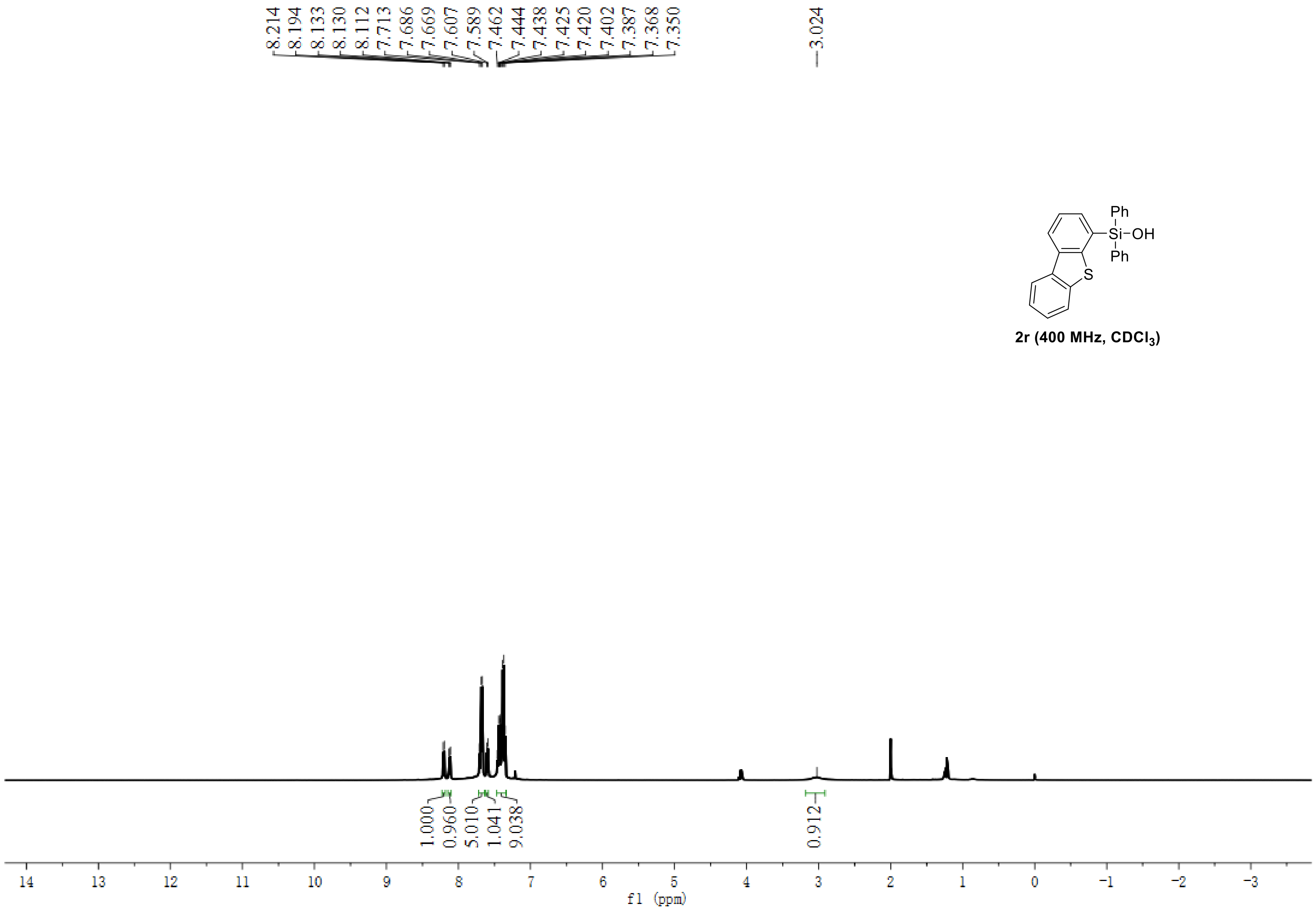

2r (400 MHz, $CDCl_3$)

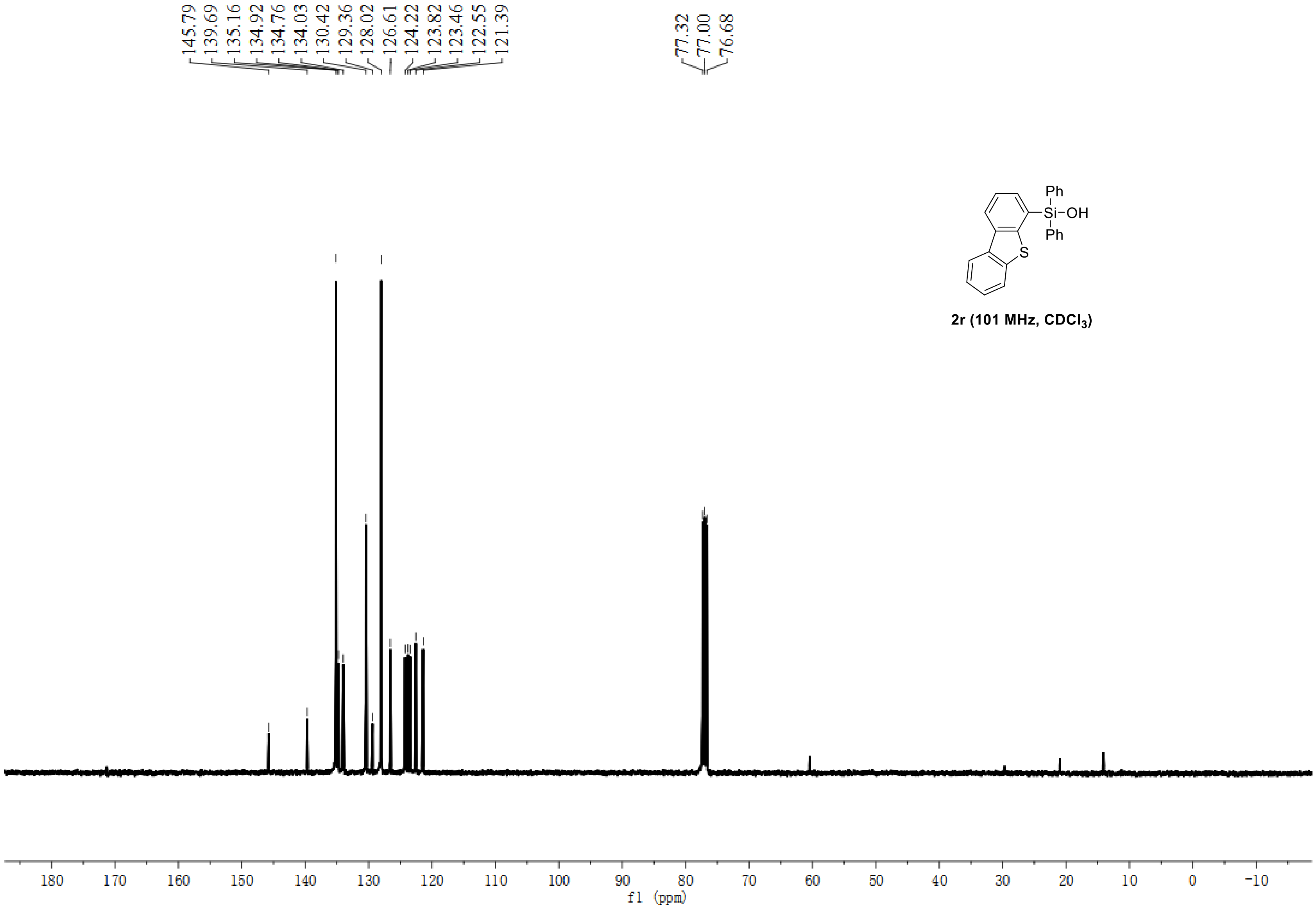

2r (101 MHz, $CDCl_3$)

2s (400 MHz, $CDCl_3$)

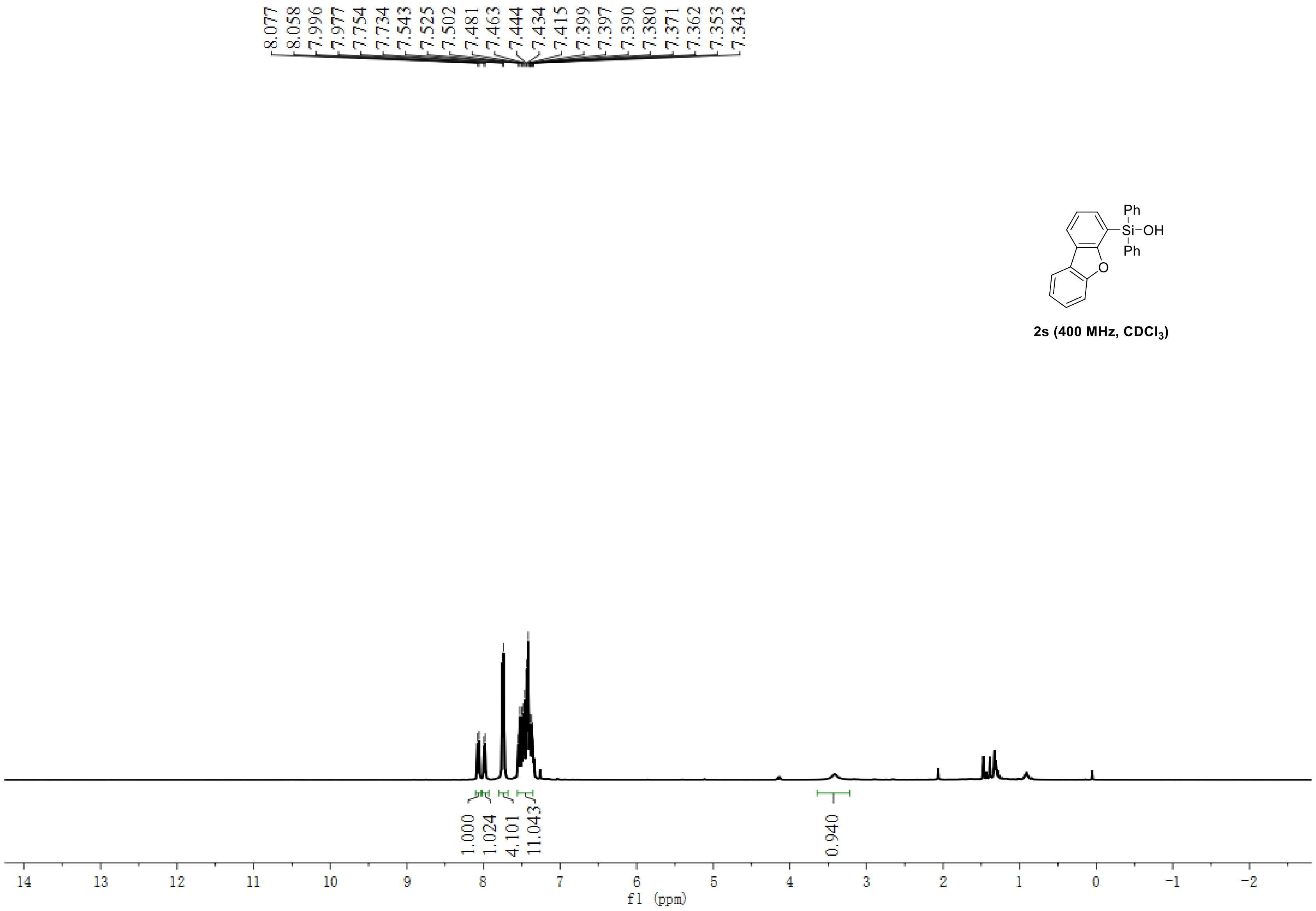

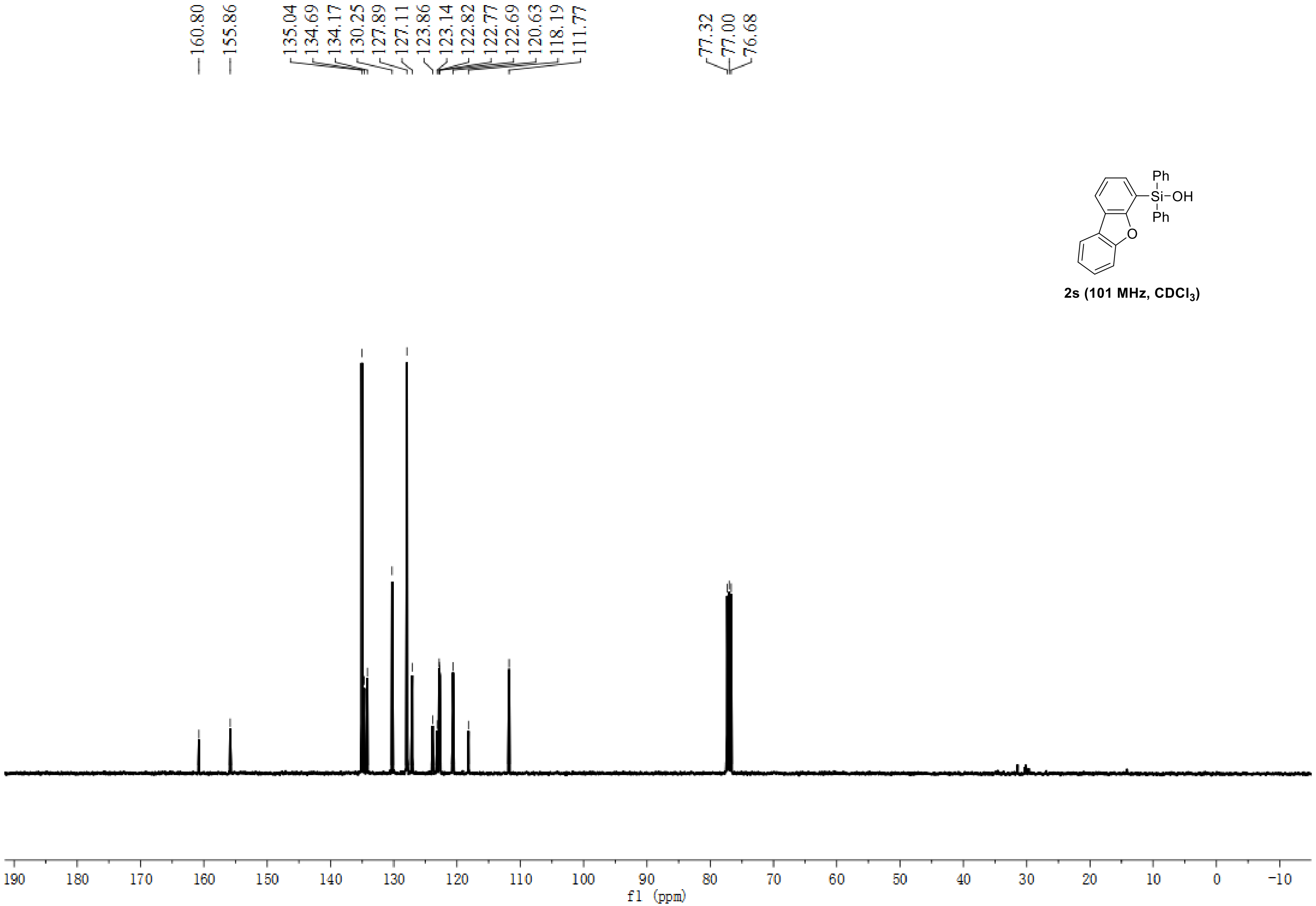

2s (101 MHz, $CDCl_3$)

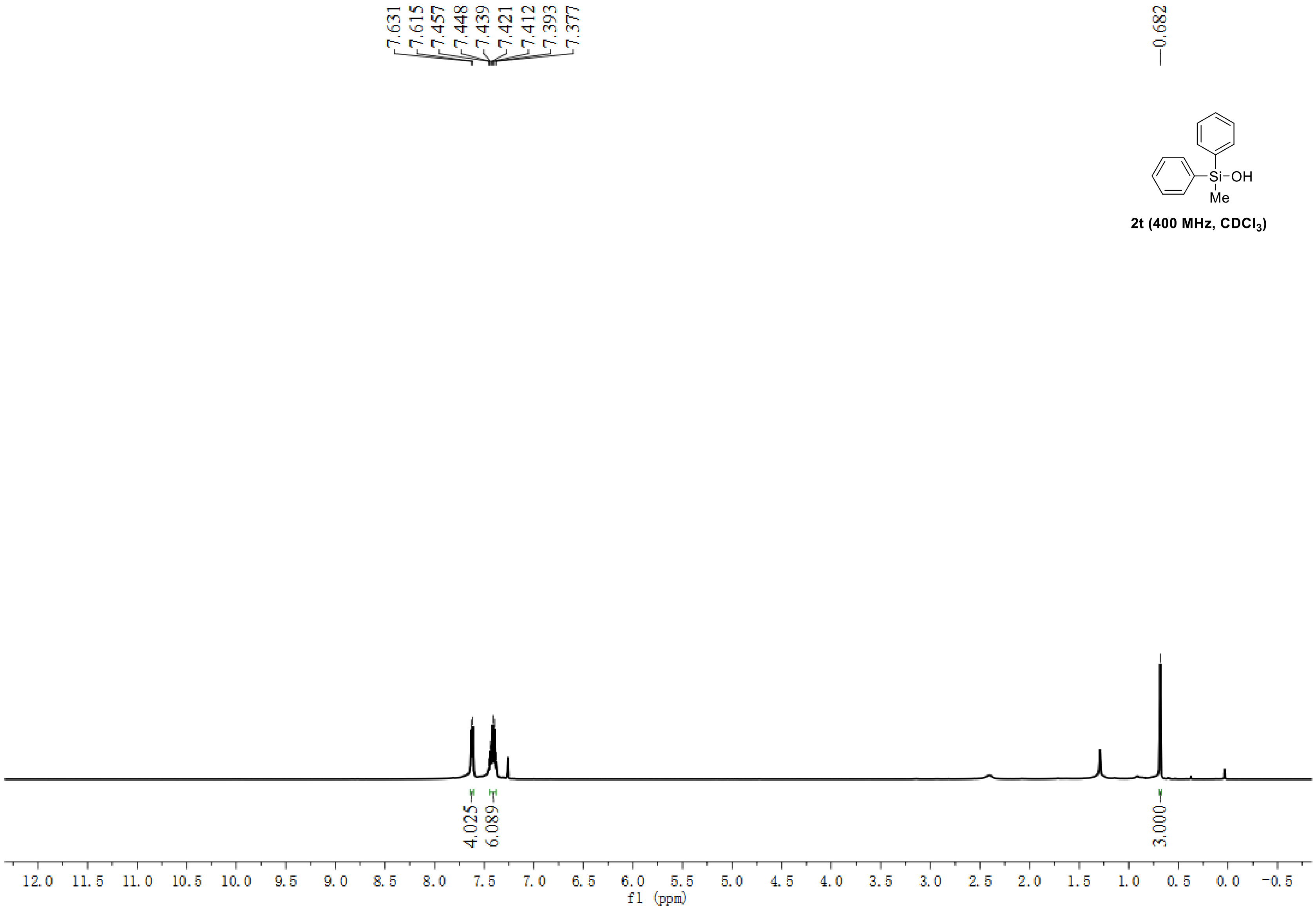

2t (400 MHz, $CDCl_3$)

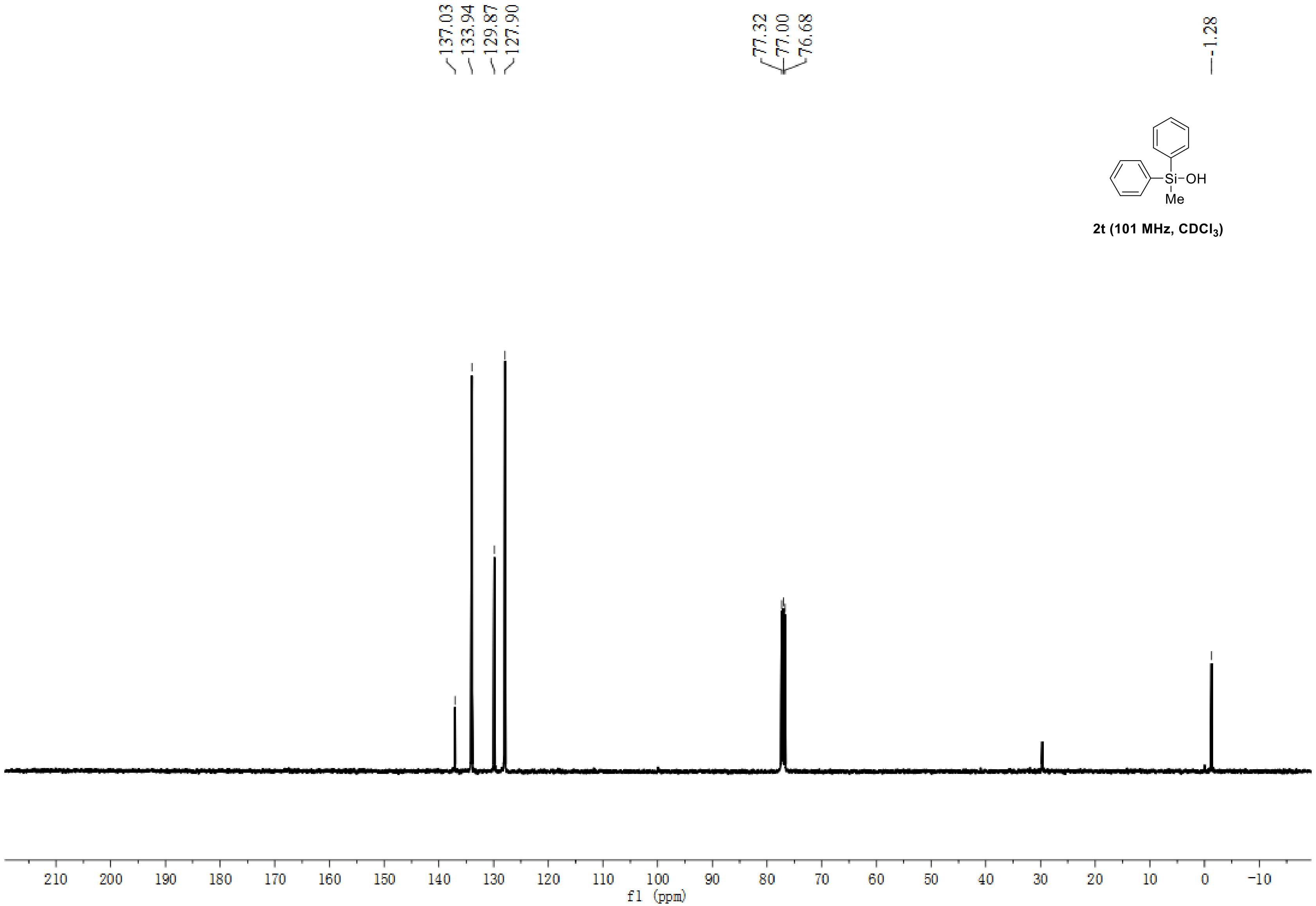
137.03
133.94
129.87
127.90
77.32
77.00
76.68
-1.28
Si
OH
Me
2t (101 MHz, CDCl3)
210
200
190
180
170
160
150
140
130
120
110
100
90
80
70
60
50
40
30
20
10
0
-10
f1 (ppm)

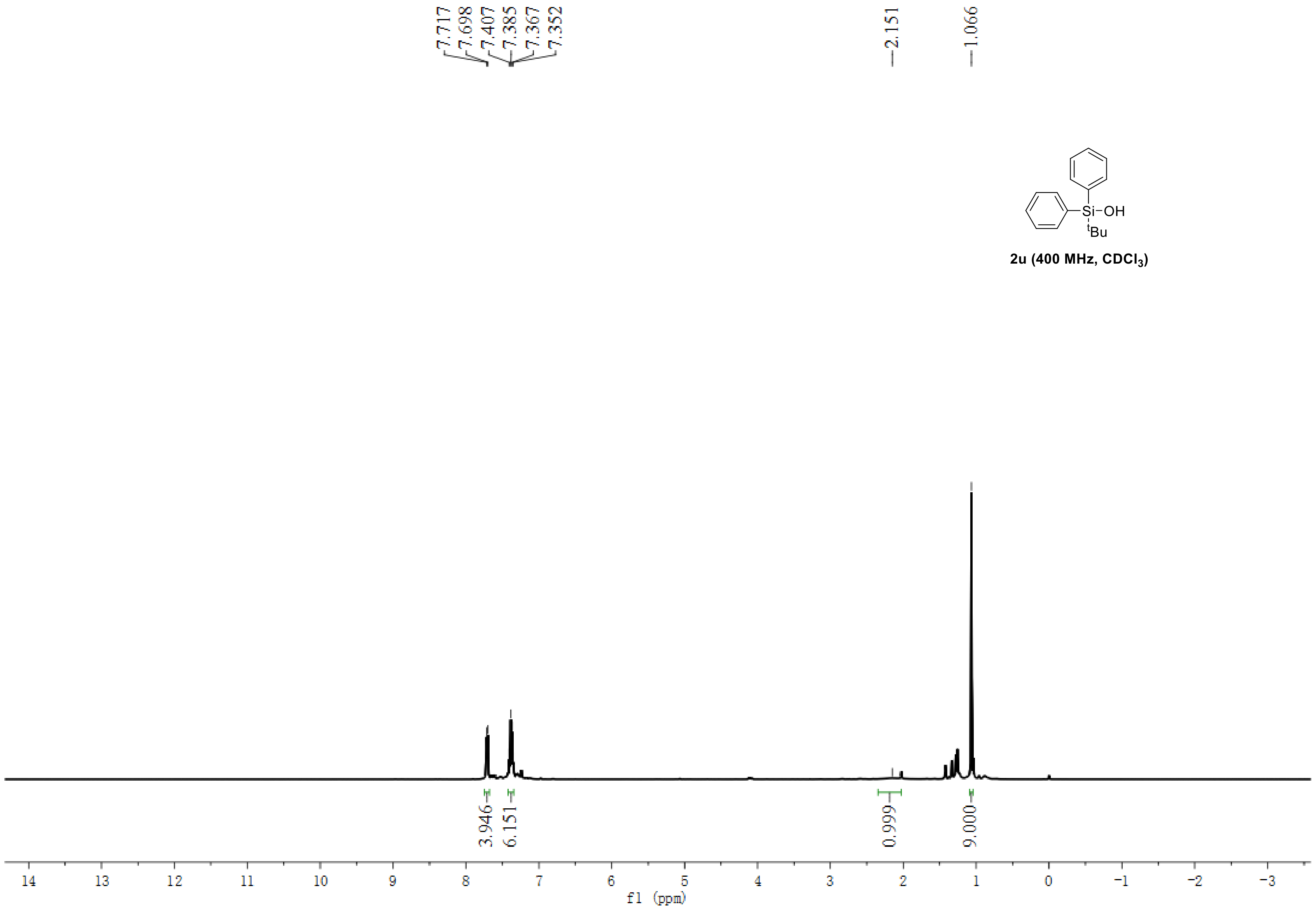

2u (400 MHz, $CDCl_3$)

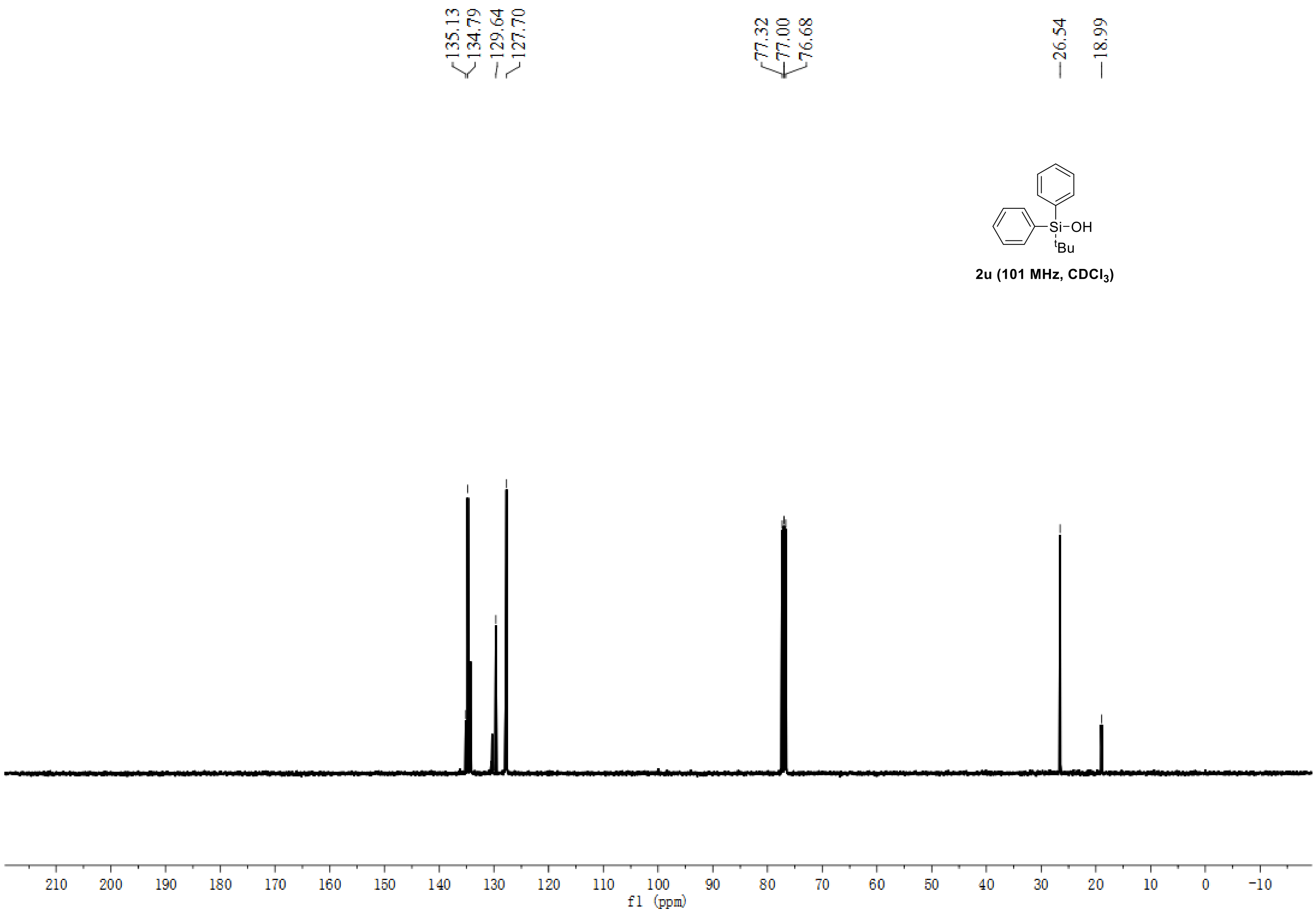
135.13
134.79
129.64
127.70
77.32
77.00
76.68
26.54
18.99
Si–OH
tBu
2u (101 MHz, CDCl3)
210
200
190
180
170
160
150
140
130
120
110
100
90
80
70
60
50
40
30
20
10
0
-10
f1 (ppm)

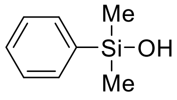

2v (400 MHz, CDCl$_3$)

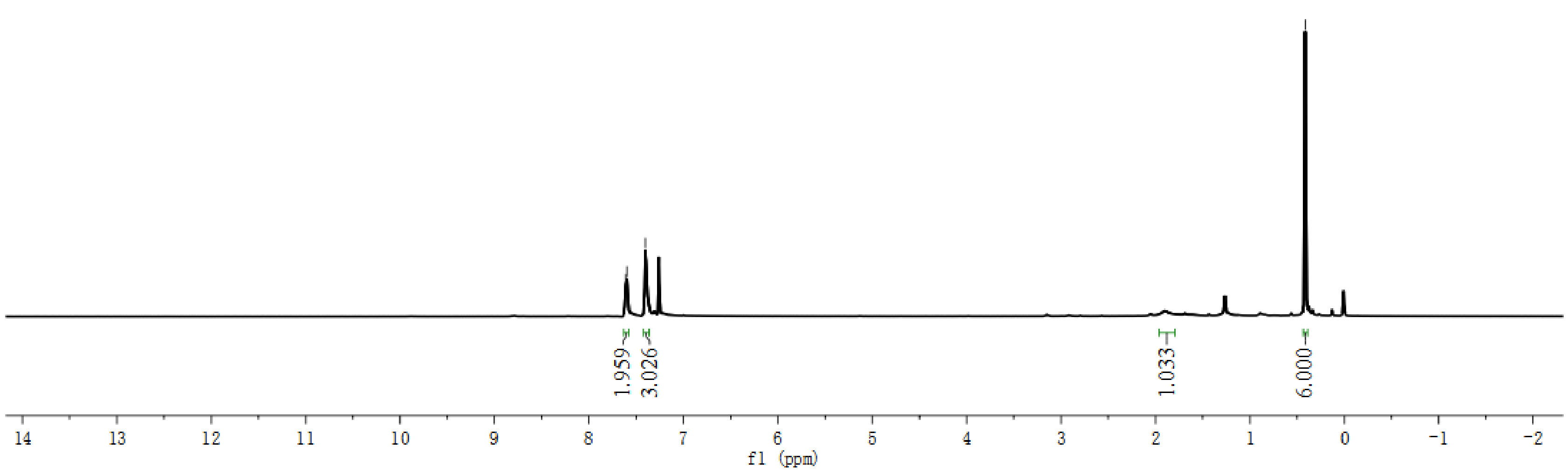

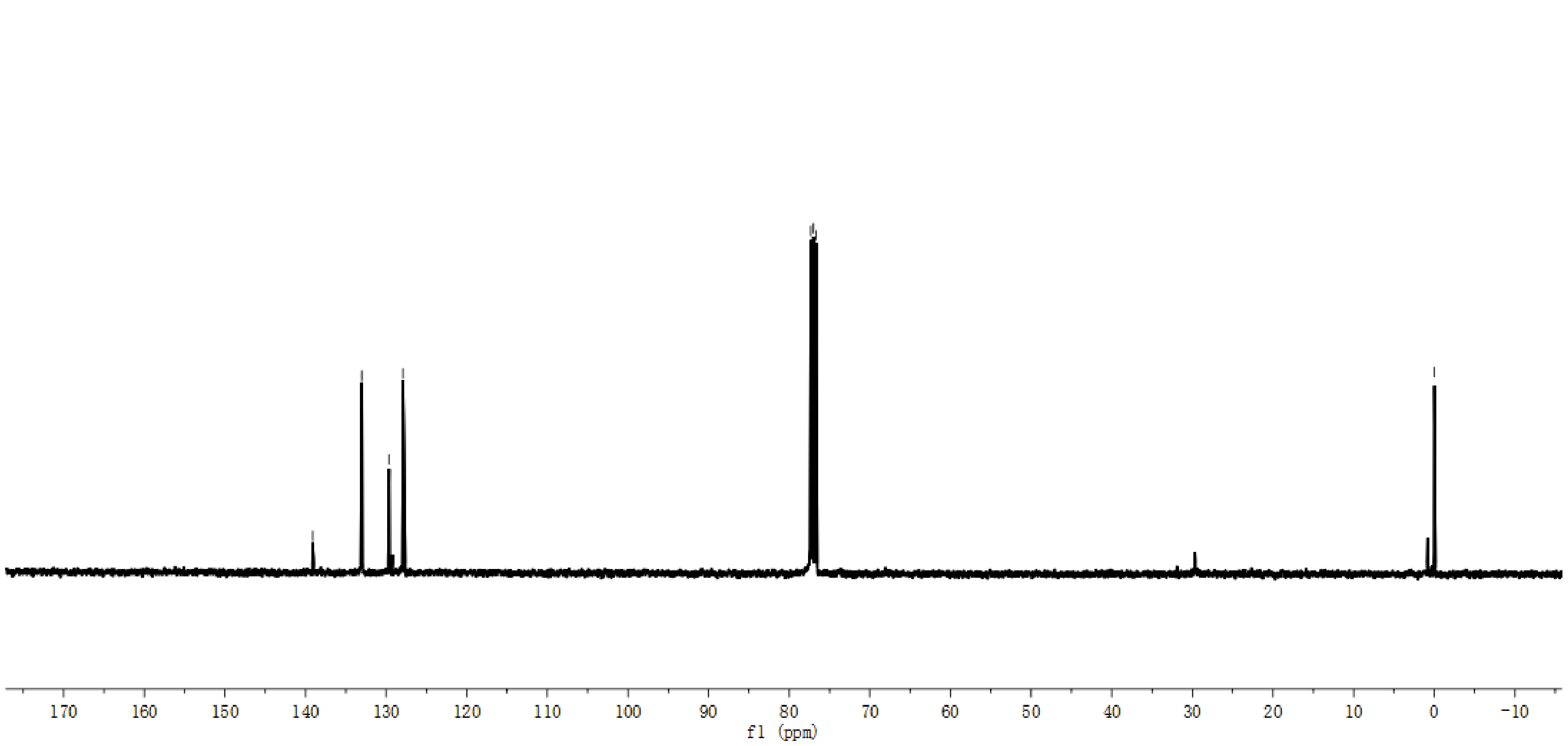

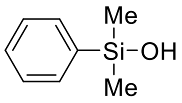

2v (101 MHz, $CDCl_3$)
